# Synthesis and Identification of 3‑Oxazolines in Cocoa

**DOI:** 10.1021/acs.jafc.5c00898

**Published:** 2025-06-05

**Authors:** Heather G. Spooner, Dimitris P. Balagiannis, Andreas Czepa, Barbara Suess, Martine Trotin, Paul O’Nion, Jane K. Parker

**Affiliations:** † Department of Food and Nutritional Sciences, 6816 University of Reading, Whiteknights, Reading RG6 6AP, U.K.; ‡ 3545 Mondele̅z International, Whiteknights Campus, Pepper Lane, Reading RG6 6LA, U.K.; § Reading Scientific Services Ltd, Whiteknights Campus, Pepper Lane, Reading RG6 6LA, U.K.

**Keywords:** strecker aldehydes, precursors, intermediates, chocolate

## Abstract

Adding water to chocolate is known to cause a large increase in the concentration of Strecker aldehydes, which are key aroma compounds in cocoa. 3-Oxazolines may be precursors responsible for this; however, only a low concentration of 2-isobutyl-5-methyl-3-oxazoline was previously identified in chocolate. This study investigates the possibility that other types of 3-oxazolines are the relevant precursors present in cocoa. A range of novel 3-oxazolines were synthesized and characterized by gas chromatography–mass spectrometry (GC–MS) and nuclear magnetic resonance spectroscopy. Using the synthesized compounds as references, four of these were identified by GC–MS for the first time in aroma extracts of cacao nibs, cocoa liquor, and chocolate, obtained by solvent-assisted flavor evaporation. This study may reveal a new focus for enhancing cocoa aroma and potentially other roasted food products as well.

## Graphical abstract

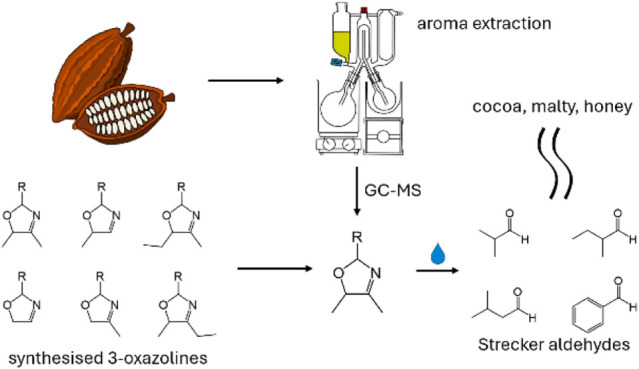

## Introduction

3-Oxazolines have been proposed as precursors, present in cocoa, which hydrolyze to form Strecker aldehydes. Granvogl et al.^1^ previously synthesized a range of 2-substituted-5-methyl-3-oxazolines and indeed showed their hydrolysis to Strecker aldehydes. However, upon searching for 2-isobutyl-5-methyl-3-oxazoline in dark chocolate, only a very low concentration was found.

Strecker aldehydes are key aroma compounds in roasted food products, such as chocolate, coffee, malt, and bread.^2,3^ They are formed during the Maillard reaction by a variety of routes but most commonly via the Strecker degradation, which is the reaction of amino acids with α-dicarbonyl compounds.^4,5^ Methionine, valine, leucine, isoleucine, phenylalanine, and alanine are six amino acids known to give rise to odor-active aldehydes. The α-dicarbonyl compounds, including methylglyoxal, 2,3-butanedione, or glucosone, arise by carbohydrate degradation, part of the Maillard reaction, or fermentation.^6^ In addition, Amadori rearrangement products are formed in the early stages of the Maillard reaction by the condensation between an amino acid and a reducing sugar, and can act as key intermediates in the formation of α-dicarbonyls, as well as being direct precursors of Strecker aldehydes themselves.^7–10^ There have also been further alternative routes to the classical Strecker degradation pathway identified, such as those that form “Strecker acids” and “Strecker amines”.^4,11^ Thus, the 3-oxazolines are formed as part of the Maillard reaction, which is a highly complex web of reactions that gives rise to the compounds that color and flavor our food, of which Strecker degradation is just a part.

Several studies have reported that the addition of water to many dry food products, such as chocolate, cornflakes and malt, results in the release of a large amount of Strecker aldehydes.^2,12,13^ Steam distillation of chocolate was found to increase the concentration of Strecker aldehydes, phenylacetaldehyde and 3-methylbutanal, by factors of ∼120 and ∼13, respectively.^14^ Although Strecker aldehydes are generally thought to be thermally formed, Ullrich et al. found that chocolate made from unroasted cocoa beans also showed a high release of Strecker aldehydes upon water treatment, postulating that an alternative water-induced reaction pathway might exist to produce these compounds from “odorless precursors already present in the unroasted cocoa beans”.^15^

The first occurrence of 3-oxazolines in the literature was reported by Rizzi who identified 2-isopropyl-4,5-dimethyl-3-oxazoline during the Strecker degradation of valine with 2,3-butanedione under nonaqueous conditions.^16^ More than 50 years later, Granvogl et al. investigated the role of 2-substituted-5-methyl-3-oxazolines as precursors of Strecker aldehydes, postulating their formation during Strecker degradation in the absence of water and remaining stable until the addition of water stimulates their hydrolysis, releasing the Strecker aldehyde.^1^

We propose that other 3-oxazolines may also be present in cocoa that are responsible for the release of Strecker aldehydes. The aim of this study was to synthesize a range of novel 3-oxazolines that we predict may be formed from known/relevant precursors and Maillard intermediates (Table 1), and search for their presence in cocoa.

**Table 1:**
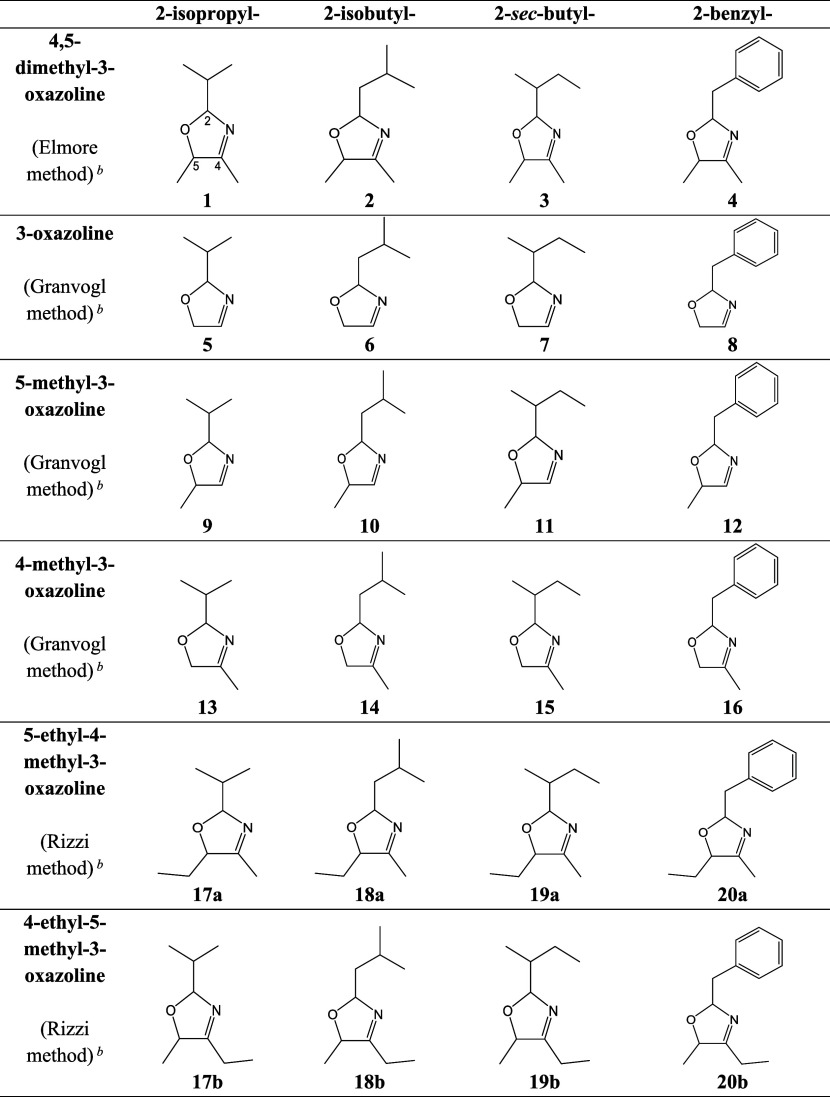
3-Oxazolines Predicted to Form in Cocoa That Were Synthesized in This Study

a The 3-oxazolines were selected on the basis of the substituent of the carbon atom at position 2 (denoted C2, labeled in compound **1**), giving rise to the Strecker aldehydes predominant in cocoa (2-methylpropanal, 3-methylbutanal, 2-methylbutanal, and phenylacetaldehyde)^22^ and the C4 and C5 substituents arising from α-dicarbonyl compounds commonly found in food (see mechanism in Figure S1 ).

b The preparation method of each 3-oxazoline depended on the class of oxazoline, defined by the C4 and C5 substituents. For details of each synthetic method, see Figure 1.

## Materials and Methods

### Materials

#### Food Samples

Cacao nibs, dark chocolate (70% cocoa), and milk chocolate (20% cocoa) were purchased from a local supermarket. Cocoa liquor was supplied by Mondele̅z International.

#### Chemicals

Ethanolamine (99%), phenylacetaldehyde (98%), 2,3-pentanedione (97%), dl-isoleucine (99%), dl-phenylalanine (99%), ethanol (99%), and diethyl ether (AR) were purchased from Thermo Scientific (Leicestershire, UK). Dichloromethane (99%), chloroform (99%), and ammonium acetate (97%) were purchased from Fisher Scientific (Leicestershire, UK). dl-1-Amino-2-propanol (93%), dl-2-amino-1-propanol (98%), Dess–Martin periodinane (95%), l-valine (98%), and l-leucine (99%) were purchased from Tokyo Chemical Industry (Oxford, UK). 2-Methylpropanal (99%), 2,3-butanedione (97%), 3-hydroxy-2-butanone (96%), pentane (98%), and deuterated chloroform (99.8%) were purchased from Sigma-Aldrich (Dorset, UK). 3-Methylbutanal (98%) and 2-methylbutanal (96%) were purchased from Alfa Aesar (Lancashire, UK). Medium-chain triglyceride was purchased from Cremer (Hamburg, Germany).

### Synthetic Methods and Characterization of 3-Oxazolines

#### Synthesis of 4,5-Dimethyl-3-oxazolines (**1**–**4**) (See Table 1)

This synthesis was adapted from a 3-thiazoline synthetic method, reported by Elmore and Mottram,^17^ replacing ammonium sulfide with ammonium acetate and removing the use of water, due to the known hydrolysis of 3-oxazolines.^1^ Ammonium acetate (10 mmol) was dissolved in ethanol (100 mL). 3-Hydroxy-2-butanone (10 mmol) and either 2-methylpropanal, 3-methylbutanal, 2-methylbutanal, or phenylacetaldehyde (to generate **1**–**4**, respectively) (5 mmol) were added and stirred at room temperature for 4 h to give an unpurified sample of the target compound in solution.

For all four compounds, ethanol was removed from the solution by distillation under reduced pressure by using a rotary evaporator (Büchi R-134 Rotavapor; Büchi Vacuum Pump V-700). A dark orange/brown liquid remained (∼0.5 mL) and was loaded onto a diol column, pre-equilibrated with redistilled pentane (SiliaSep OT Flash Cartridges, Silica-Based Diol nec, 50 g, 150 mL, 40–63 μm, 60 A).

#### 2-Isopropyl-4,5-dimethyl-3-oxazoline (**1**)

The elution was performed as follows: 50 mL of pentane/CDCl_3_ (95:5; v/v), collected in 25 mL fractions; 50 mL of pentane/CDCl_3_ (90:10; v/v), followed by 25 mL of pentane/CDCl_3_ (85:15; v/v), collected in 5 mL fractions. Fraction 9 contained both diastereomers in the best purity, with peak percentage areas (GC–MS) of 45.5% and 51.6%, respectively (excluding solvent). ^1^H NMR (400 MHz; CDCl_3_) δ 0.91 [d, *J* = 6.8 Hz, 3H, H–C­(9 or 9′′ or 10 or 10′)], 0.95 [d, *J* = 6.8 Hz, 6H, H–C­(9 or 9′ or 10 or 10′)], 0.96 [d, *J* = 6.8 Hz, 3H, H–C­(9 or 9′ or 10 or 10′)], 1.29 [d, *J* = 6.7 Hz, 3H, H–C­(7 or 7′)], 1.34 [d, *J* = 6.7 Hz, 3H, H–C­(7 or 7′)], 1.83–1.96 [m, 2H, H–C­(8, 8′)], 2.00 [d, *J* = 1.9 Hz, 3H, H–C­(6 or 6′)], 2.02 [d, *J* = 1.7 Hz, 3H, H–C­(6 or 6′)], 4.58–4.63 [m, *J* = 3.3 Hz, 1H, H–C­(5 or 5′)], 4.64–4.70 [m, *J* = 6.3 Hz, 1H, H–C­(5 or 5′)], 5.27–5.30 [m, *J* = 2.1 Hz, 1H, H–C­(2 or 2′)], 5.44–5.48 [m, *J* = 1.9 Hz, 1H, H–C­(2 or 2′)]; ^13^C NMR (100 MHz; CDCl_3_) δ 14.05 [C­(6 or 6′)], 15.17 [C­(6 or 6′)], 16.62 [C­(9 or 9′ or 10 or 10′)], 17.04 [C­(9 or 9′ or 10 or 10′)], 17.42 [C­(9 or 9′ or 10 or 10′)], 17.44 [C­(9 or 9′ or 10 or 10′)], 18.87 [C­(7 or 7′)], 18.56 [C­(7 or 7′)], 33.15 [C­(8 or 8′)], 33.66 [C­(8 or 8′)], 82.31 [C­(5 or 5′)], 82.76 [C­(5 or 5′)], 108.55 [C­(2 or 2′)], 108.63 [C­(2 or 2′)], 172.03 [C­(4, 4′)].

#### 2-Isobutyl-4,5-dimethyl-3-oxazoline (**2**)

The elution was performed as follows: 50 mL of pentane/CDCl_3_ (95:5; *v*/*v*), collected in 25 mL fractions; 80 mL of pentane/CDCl_3_ (90:10; *v*/*v*), followed by 20 mL of pentane/CDCl_3_ (85:15; *v*/*v*), collected in 5 mL fractions. Fraction 9 contained both diastereomers, visible as one peak with a peak percentage area (GC–MS) of 94.1% (excluding solvent). ^1^H NMR (400 MHz; CDCl_3_) δ 0.96 [d, *J* = 6.8 Hz, 6H, H–C­(10 or 10′ or 11 or 11′)], 0.97 [d, *J* = 6.7 Hz, 6H, H–C­(10 or 10′ or 11 or 11′)], 1.29 [d, *J* = 6.7 Hz, 3H, H–C­(7 or 7′)], 1.33 [d, *J* = 6.7 Hz, 3H, H–C­(7 or 7′)], 1.39–1.52 [m, *J* = 4.9 Hz, 2H, H–C­(8a, 8a′)], 1.54–1.64 [m, *J* = 4.4 Hz, 2H, H–C­(8b, 8b′)], 1.81–1.95 [m, *J* = 4.0 Hz, 2H, H–C­(9, 9′)], 1.99–2.00 [m, *J* = 1.7 Hz, 6H, H–C­(6, 6′)], 4.56–4.62 [m, *J* = 3.3 Hz, 1H, H–C­(5 or 5′)], 4.66–4.72 [m, *J* = 6.3 Hz, 1H, H–C­(5 or 5′)], 5.47–5.53 [m, *J* = 1.8 Hz, 1H, H–C­(2 or 2′)], 5.64–5.69 [m, *J* = 1.7 Hz, 1H, H–C­(2 or 2′)]; ^13^C NMR (100 MHz; CDCl_3_) δ 15.26 [C­(6 or 6′)], 15.28 [C­(6 or 6′)], 18.29 [C­(7 or 7′)], 19.69 [C­(7 or 7′)], 22.62 [C­(10 or 10′ or 11 or 11′)], 22.67 [C­(10 or 10′ or 11 or 11′)], 23.05 [C­(10 or 10′ or 11 or 11′)], 23.10 [C­(10 or 10′ or 11 or 11′)], 24.75 [C­(9 or 9′)], 45.07 [C­(8 or 8′)], 46.36 [C­(8 or 8′)], 81.83 [C­(5 or 5′)], 82.25 [C­(5 or 5′)], 103.17 [C­(2 or 2′)], 103.21 [C­(2 or 2′)], 171.25 [C­(4 or 4′)], 171.34 [C­(4 or 4′)].

#### 2-*sec*-Butyl-4,5-dimethyl-3-oxazoline (**3**)

The elution was performed as described above for **2**. Fraction 7 contained both diastereomers as separate peaks, with peak percentage areas (GC–MS) of 57.0% and 38.8%, respectively (excluding solvent). ^1^H NMR (400 MHz; CDCl_3_) δ 0.83–0.89 [m, *J* = 4.3 Hz, 3H, H–C­(11, 11′)], 0.92–0.96 [m, *J* = 4.2 Hz, 3H, H–C­(10, 10′)], 1.14–1.25 [m, *J* = 2.7 Hz, 2H, H–C­(9a, 9a′)], 1.29 [dd, *J* = 1.5 Hz, 6.6 Hz, 2H, H–C­(7 or 7′)], 1.33 [dd, *J* = 0.9 Hz, 6.6 Hz, 1H, H–C­(7 or 7′)], 1.50–1.58 [m, *J* = 2.9 Hz, 1H, H–C­(9b, 9b′)], 1.64–1.75 [m, *J* = 2.3 Hz, 1H, H–C­(8, 8′)], 1.99–1.99 [m, *J* = 1.0 Hz, 1H, H–C­(6 or 6′)], 2.02 [d, *J* = 1.8 Hz, 2H, H–C­(6 or 6′)], 4.56–4.63 [m, *J* = 3.5 Hz, 1H, H–C­(5 or 5′)], 4.63–4.70 [m, *J* = 3.2 Hz, 1H, H–C­(5 or 5′)], 5.33–5.40 [m, *J* = 2.1 Hz, 1H, H–C­(2 or 2′)], 5.52–5.58 [m, *J* = 2.0 Hz, 1H, H–C­(2 or 2′)]; ^13^C NMR (100 MHz; CDCl_3_) δ 11.71 [C­(10 or 10′)], 11.78 [C­(10 or 10′)], 13.36 [C­(11 or 11′)], 13.91 [C­(11 or 11′)], 15.19 [C­(6, 6′)], 18.70 [C­(7 or 7′)], 18.83 [C­(7 or 7′)], 24.56 [C­(9 or 9′)], 24.97 [C­(9 or 9′)], 40.25 [C­(8 or 8′)], 40.46 [C­(8 or 8′)], 82.27 [C­(5 or 5′)], 82.58 [C­(5 or 5′)], 107.63 [C­(2 or 2′)], 107.94 [C­(2 or 2′)], 171.68 [C­(4 or 4′)], 171.94 [C­(4 or 4′)].

#### 2-Benzyl-4,5-dimethyl-3-oxazoline (**4**)

The elution was performed as follows: 50 mL pentane/CDCl_3_ (90:10; *v*/*v*), collected in 25 mL fractions; 50 mL pentane/CDCl_3_ (90:10; *v*/*v*), followed by 50 mL pentane/CDCl_3_ (85:15; *v*/*v*), followed by 20 mL pentane/CDCl_3_ (80:20; *v*/*v*) collected in 5 mL fractions. Fraction 8 contained both diastereomers in the highest purity, with peak percentage areas (GC–MS) of 25.6% and 19.5% (excluding solvent). ^1^H NMR (400 MHz; CDCl_3_) δ 1.09 [d, *J* = 6.7 Hz, 1H, H–C­(7 or 7′)], 1.23 [d, *J* = 6.7 Hz, 2H, H–C­(7 or 7′)], 1.92 [d, *J* = 1.3 Hz, 2H, H–C­(6 or 6′)], 1.94 [d, *J* = 1.4 Hz, 1H, H–C­(6 or 6′)], 2.56–2.74 [m, *J* = 5.9 Hz, 2H, H–C­(8a, 8b or 8a′, 8b′)], 2.88–3.09 [m, *J* = 4.3 Hz, 4H, H–C­(8a, 8b or 8a′, 8b′)], 4.36–4.42 [m, *J* = 6.3 Hz, 1H, H–C­(5 or 5′)], 4.54–4.61 [m, *J* = 4.2 Hz, 1H, H–C­(5 or 5′)], 5.72–5.7 [m, *J* = 1.8 Hz, 1H, H–C­(2 or 2′)], 5.88–5.93 [m, *J* = 1.7 Hz, 1H, H–C­(2 or 2′)], 7.16–7.20 [m, *J* = 3.0 Hz, 5H, H–C­(10, 11, 12, 13, 14, 10′, 11′, 12′, 13′, 14′)]; ^13^C NMR (100 MHz; CDCl_3_) δ 15.06 [C­(6 or 6′)], 15.12 [C­(6 or 6′)], 18.40 [C­(7 or 7′)], 19.11 [C­(7 or 7′)], 42.20 [C­(8 or 8′)], 42.99 [C­(8 or 8′)], 82.60 [C­(5 or 5′)], 82.94 [C­(5 or 5′)], 104.33 [C­(2 or 2′)], 104.55 [C­(2 or 2′)], 126.31 [C­(12 or 12′)], 126.38 [C­(12 or 12′)], 128.00 [C­(10 or 10′ or 14 or 14′)], 128.02 [C­(10 or 10′ or 14 or 14′)], 130.06 [C­(11 or 11′ or 13 or 13′)], 130.13 [C­(11 or 11′ or 13 or 13′)], 136.64 [C­(9 or 9′)], 136.67 [C­(9 or 9′)], 172.26 [C­(4 or 4′)], 172.48 [C­(4 or 4′)].

#### Synthesis of 3-Oxazolines, 5-Methyl-3-oxazolines, and 4-Methyl-3-oxazolines (**5**–**16**)

This synthesis was based on a method for 2-substituted-5-methyl-3-oxazolines, reported by Granvogl et al.,^1^ but the amino alcohol reagent was varied to generate the desired substituents at positions 4 and 5. For the 4,5-unsubstituted-3-oxazolines, ethanolamine (5 mmol) was reacted with either 2-methylpropanal, 3-methylbutanal, 2-methylbutanal, or phenylacetaldehyde (to generate **5**–**8**, respectively) (5 mmol) in dichloromethane (20 mL). For the 5-methyl-3-oxazolines, 1-amino-2-propanol (5 mmol) was reacted with either 2-methylpropanal, 3-methylbutanal, 2-methylbutanal, or phenylacetaldehyde (to generate **9**–**12**, respectively) (5 mmol) in dichloromethane (20 mL). Finally, for the 4-methyl-3-oxazolines, 2-amino-1-propanol (5 mmol) was reacted with either 2-methylpropanal, 3-methylbutanal, 2-methylbutanal, or phenylacetaldehyde (to generate **13**–**16**, respectively) (5 mmol) in dichloromethane (20 mL). Each reaction mixture was stirred for approximately 15 h in order to obtain the respective oxazolidine. The mixture was diluted with dichloromethane (60 mL), Dess–Martin periodinane (3.5 mmol) was added to oxidize the oxazolidine to the corresponding 3-oxazoline, and the mixture was stirred for 30 min. Redistilled pentane (25 mL) was added, and the reaction mixture was filtered and concentrated to ∼5 mL using a Vigreux column in a 40 °C water bath to give an unpurified sample of the target compound in solution.

#### Synthesis of 4/5-Ethyl–4/5-Methyl-3-oxazolines (**17**–**20**)

This method was based on a synthesis for 2-isopropyl-4,5-dimethyl-3-oxazoline, reported by Rizzi,^16^ replacing valine with a range of amino acids and 2,3-butanedione with 2,3-pentanedione. 2,3-Pentanedione (75 mmol) was mixed with either l-valine, l-leucine, dl-isoleucine, or dl-phenylalanine (to generate **17**–**20**, respectively) (75 mmol) in 25 mL of medium-chain triglyceride (MCT) and refluxed for approximately 45 min to generate the Maillard products formed in the absence of water. Redistilled diethyl ether (25 mL) was added, and the reaction mixture was filtered and submitted to solvent-assisted flavor evaporation (SAFE) distillation at ∼50 °C to remove MCT. The mixture was concentrated to ∼5 mL by using a Vigreux column in a 40 °C water bath to give an unpurified sample of the target compound in solution.

Purification and NMR analysis of 2-isopropyl-5-ethyl-4-methyl-3-oxazoline (**17a**) and 2-isopropyl-4-ethyl-5-methyl-3-oxazoline (**17b**) were carried out. The concentrated sample (∼5 mL) was loaded onto a diol column (SiliaSep OT Flash Cartridges, Silica-Based Diol nec, 50 g, 150 mL, 40–63 μm, 60 A), which was pre-equilibrated with redistilled pentane. The elution was performed as follows: 200 mL of pentane, collected in 25 mL fractions; 200 mL of pentane/chloroform (95:5; *v*/*v*), followed by 100 mL of pentane/chloroform (92.5:7.5; *v*/*v*), collected in 10 mL fractions. Fraction 3 contained both **17a** and **17b** in the highest purity, although impurities were present, with peak percentage areas (GC–MS) of 8.5% and 27.2% for the diastereomers of **17a**, and 1.7% and 12.6% for the diastereomers of **17b** (excluding solvent).

### Extraction and Identification of 3-Oxazolines in Cacao Nibs, Cocoa Liquor, and Chocolate

#### SAFE Extraction^18^

Roasted cacao nibs (200 g), roughly ground in a coffee grinder (De’Longhi KG210); dark or milk chocolate (200 g), roughly chopped by hand; or cocoa liquor (250 g), roughly chopped, frozen in liquid nitrogen, and ground to a powder using a coffee grinder, were stirred with dichloromethane (600 mL) for ∼15 h at room temperature. The mixture was vacuum-filtered and combined with the dichloromethane washes (2 × 20 mL for cacao nibs and chocolate; 400 mL for cocoa liquor) before being subjected to SAFE distillation at 40 °C. The thawed SAFE distillate was concentrated to ∼0.5 mL in a 40 °C water bath using Vigreux columns and stored at −80 °C.

#### Identification by GC–MS

All samples of synthesized 3-oxazolines and SAFE extracts were analyzed by direct liquid injection (1 μL) by GC–MS. Due to the desire to run samples on columns of different polarities and to obtain accurate masses, many different GC–MS systems were used. For the nonpolar column, samples were manually injected in split mode 2:1, injector temperature 250 °C, flow 2 mL/min onto an HP-5 UI column (30 m × 0.25 mm × 1 μm; Agilent) with an Agilent 7890*B*/7693 GC-QToF system (Agilent, Santa Clara, CA). The oven temperature was held at 40 °C for 2 min, increased to 320 °C at a ramp of 4 °C/min, and held for 5 min. The carrier gas was helium at a flow rate of 1 mL/min. The mass spectrometer was used in both electron ionization (EI) mode and chemical ionization (CI) mode. In EI mode, the source temperature was 230 °C, ionization voltage 70 eV and scan range *m*/*z* 40 to *m*/*z* 210. In CI mode, the source temperature was 300 °C, ionization voltage 175 eV and scan range *m*/*z* 80 to *m*/*z* 220. The data were processed by using Agilent Masshunter software.

For the polar column, samples were manually injected (1 μL) in the splitless mode, with the injector at 250 °C, onto a ZB-Wax column (30 m × 0.25 mm × 1 μm; Phenomenex) with an Agilent 7890*A*/5975C GC–MS system as well as a DB-Wax UI column (30 m × 0.25 mm × 0.25 μm; Agilent) with an Agilent 6890/5975 GC–MS system. The oven temperature was increased from 40 to 250 °C at a ramp of 4 °C/min, and held for 10 min. The carrier gas was helium at a flow of 1 mL/min. Both mass spectrometers were operated in EI mode with a source temperature of 230 °C and ionization voltage of 70 eV. The scan range was from *m*/*z* 29 to *m*/*z* 400 (7890*A*/5975C system) or *m*/*z* 20 to *m*/*z* 400 (6890/5975 system). Selected ion monitoring (SIM) was applied for *m/z* 70, 84, 98, and 112 with a dwell time of 50 ms each. The data were processed by using Agilent MSD ChemStation.

In order to separate enantiomers, the synthesized 4,5-dimethyl-3-oxazolines (**1**–**4**) were run on a chiral column. Samples were injected in the splitless mode, with an injector temperature of 250 °C, onto a CP-Chirasil-Dex CB column (25 m × 0.25 mm × 0.25 μm; Agilent) with an Agilent 6890/5975 GC–MS system. The oven temperature was increased from 40 to 200 °C at a ramp of 4 °C/min and held for 20 min. The carrier gas was helium at a flow of 1 mL/min. The mass spectrometer was operated in EI mode with a source temperature of 230 °C, ionization voltage of 70 eV, and scan range of *m*/*z* 10 to *m*/*z* 400.

A series of *n*-alkanes (C_5_–C_30_) was also run under the same conditions in order to calculate the linear retention index (LRI) of each compound on each column.

#### Two-Dimensional Gas Chromatography–Mass Spectrometry (2D-GC–MS)

To obtain better separation of the compounds, a GC/GC–MS system was employed. The system consisted of a nonpolar HP-5MS 5% diphenyl column (30 m × 0.25 mm × 0.25 μm; Agilent) and a polar VF17 ms 17% diphenyl column (2 m × 0.1 mm × 0.2 μm; Agilent) with an Agilent 8890/7250 GC-QToF system and ZX2 thermal modulator (Zoex, Houston, TX). The oven temperature was increased from 50 to 300 °C at a ramp of 5 °C/min and held for 10 min. Samples were injected by an automatic liquid sampler in the split mode 5:1, injector temperature of 250 °C, helium flow of 4 mL/min onto the nonpolar column, and transferred to the polar column via a modulator column of deactivated silica (1 m × 0.1 mm; Zoex), formed into a double loop, which was periodically chilled at −90 °C for 5 s and heated at 450 °C for 300 ms. The mass spectrometer was used in EI mode with a source temperature of 200 °C, a scan range of *m*/*z* 33 to *m*/*z* 600, and an acquisition rate of 50 spectra/s. The data were processed by using a GC Image 2024 GCxGC.

#### Gas Chromatography–Olfactometry (GC-O)

Synthesized compounds **1**–**4** were analyzed for odor characteristics by GC-O. Samples were manually injected (1 μL) in splitless mode, with the injector at 250 °C, onto an HP-5MS UI column (30 m × 0.25 mm × 0.25 μm; Agilent) with an Agilent 7890B GC equipped with a flame ionization detector (Hewlett-Packard, Waldbronn, BaWü, Germany) and an ODO II odor port (SGE, Ringwood, Victoria, Australia). The oven temperature was increased from 40 to 200 °C at a ramp of 4 °C/min, then increased to 300 °C at a ramp of 8 °C/min, and held for 8 min. The carrier gas was helium at a flow of 2 mL/min. The column effluent was split equally between the FID and odor port, where the odors of the eluting compounds were evaluated on the basis of matching LRI by 5 assessors.

A series of *n*-alkanes (C_5_–C_30_) was also run under the same conditions in order to calculate the linear retention index (LRI) of each compound.

#### NMR Spectroscopy

^1^H, ^13^C, COSY, HSQC, and HMBC NMR spectra were recorded by using a Bruker Nanobay 400 MHz spectrometer (Bruker, Rheinstetten, Germany). For preparation, samples (1 mL) were evaporated to dryness under a gentle stream of nitrogen, and CDCl_3_ (0.7 mL) was added. The data were processed using Bruker TopSpin 4.4.0, and chemical shifts were determined using the proton signal (7.26 ppm; ^1^H NMR) or carbon signal (77.0 ppm; ^13^C NMR) of CDCl_3_.

## Results and Discussion

### Synthesis and Characterization of 4,5-Dimethyl-3-oxazolines (**1**–**4**)

We hypothesized that many different types of 3-oxazolines could be formed based on the availability of precursors and Maillard intermediates present in cocoa, with the potential to release Strecker aldehydes upon the addition of water. Of those that were successfully synthesized and identified in reaction mixtures (∼20), the 4,5-dimethyl-3-oxazolines were consistently identified in the cocoa samples analyzed (Section 3.2). In order to confirm the identity of these 4,5-dimethyl-3-oxazolines (**1**–**4**) in cocoa, the reaction mixtures were purified, and the compounds were fully characterized (Figure 1). In food, they are theoretically generated from the reaction of 2,3-butanedione, an α-dicarbonyl compound that is generated in the early stages of the Maillard reaction, with one of the four amino acids abundant in cocoa: valine, leucine, isoleucine, and phenylalanine.^16^

**Figure 1:**
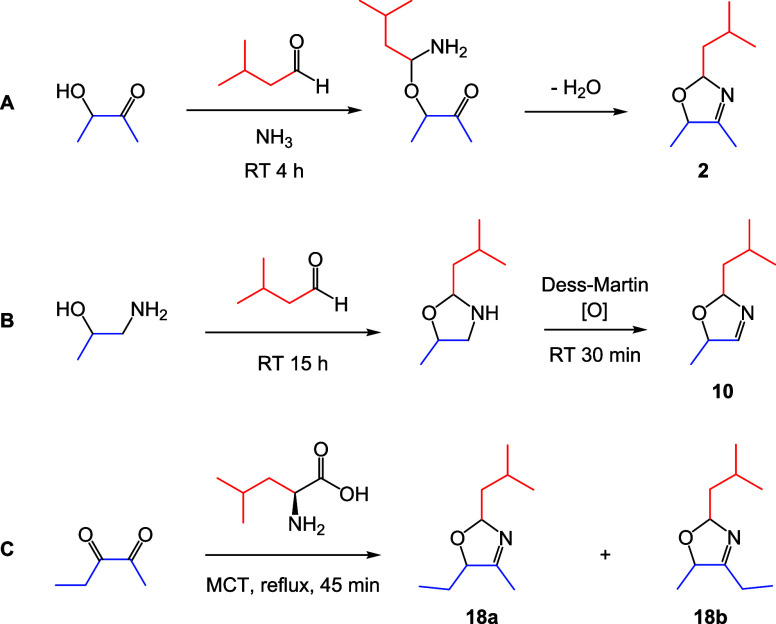
Examples of the three synthetic routes used to generate 3-oxazolines. (A) The Elmore method, used for the preparation of **1**–**4**, adapted from 3-thiazoline synthesis, reported by Elmore and Mottram.^17^ (B) The Granvogl method, used for the preparation of **5**–**16**, adapted from the synthesis for 2-substituted-5-methyl-3-oxazolines, reported by Granvogl et al.^1^ The amino alcohol reagent was varied in order to control the substituents at C4 and C5. (C) The Rizzi method, used for the preparation of **17**–**20**, adapted from the synthesis for 2-isopropyl-4,5-dimethyl-3-oxazoline, reported by Rizzi.^16^

Depending on the number of chiral centers, multiple stereoisomers of each oxazoline were expected. Compounds **1**, **2**, and **4**, possessing two chiral centers, formed four stereoisomers, existing as two pairs of enantiomers that are diastereomers of each other (Figure 2A). This was confirmed by 1D-GC–MS analysis on both an achiral (HP-5 and ZB-Wax) and chiral (CP-Chirasil-Dex CB) column, which displayed two peaks (diastereomeric separation) and four peaks (enantiomeric separation), respectively. Compound **3**, possessing three chiral centers due to the *sec*-butyl group, formed 8 stereoisomers, which were observed with the chiral column.

**Figure 2:**
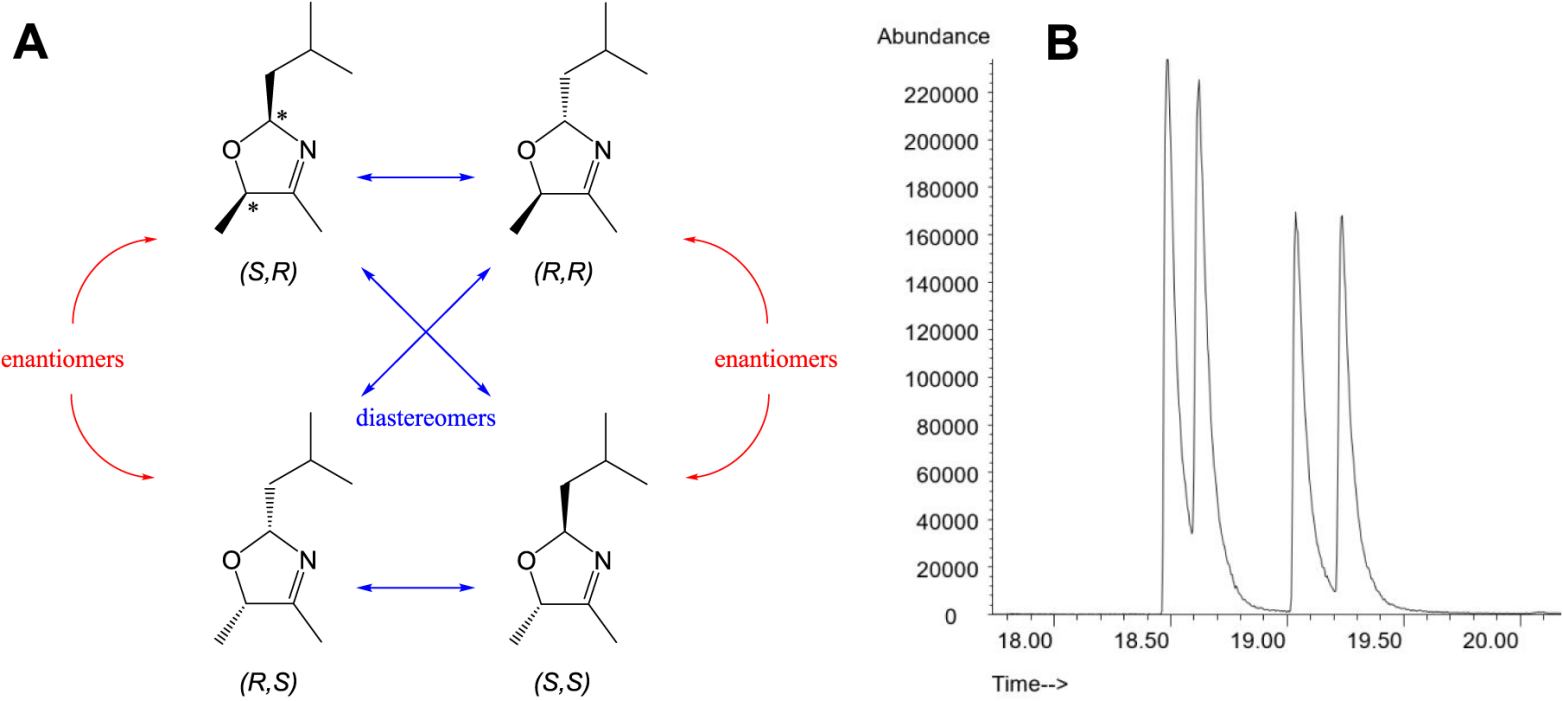
Stereoisomers of 2-isobutyl-4,5-dimethyl-3-oxazoline (**2**). (A) Two chiral centers, indicated by asterisks, gives rise to four stereoisomers, existing as two pairs of enantiomers that are diastereomers of each other, assigned as (*S*) or (*R*) by the Cahn–Ingold–Prelog rules. (B) All four stereoisomers were observed on a chiral GC–MS column (CP-Chirasil-Dex CB).

The four synthesized 4,5-dimethyl-3-oxazolines were purified and characterized by GC–MS (Table 2), and their structures were confirmed by NMR spectroscopy. Figure 3 shows the HMBC spectrum of 2-isobutyl-4,5-dimethyl-3-oxazoline (**2**). The complete annotated set of ^1^H, ^13^C, COSY, HSQC, and HMBC NMR data for **1**–**4** is available in Figure S2. In both the ^1^H and ^13^C spectra, duplicate signals were observed for each unique environment due to diastereomeric separation. It was not possible to assign the diastereomers since they were not separated by column chromatography; therefore the diastereomeric proton signals were integrated together. However, some diastereomeric signals in the ^1^H NMR spectrum of **3** were clearly separated, suggesting a 3:2 ratio (see Figure S2.11). The intensity of the blobs shown in the 2D-GC–MS image suggested this. In Figure 3A, the key signals have been highlighted in colored boxes, and their corresponding relationships within **2** are indicated in Figure 3B. The relationships of C4, C5, and H5 (highlighted in blue and pink) confirm the 3-oxazoline ring structure, while the relationships of C2 and C9 (highlighted in green and orange) confirm the isobutyl group attached to C2. These NMR data show a good similarity to those previously reported by Granvogl et al.^1^ for 5-methyl-3-oxazolines and by Rizzi^16^ for 2-isopropyl-4,5-dimethyl-3-oxazoline, with the multiplet signals for H2 and H5 between 4 and 6 ppm being characteristic of 3-oxazolines.

**Table 2:** GC–MS Characterization of All Synthesized 3-Oxazolines^a^ ^b^

		LRI		MS fragmentation (EI)^a^	[M + H] exact mass (CI)^b^	
	**Compound**	HP-5	**ZB-Wax**	*m*/*z* (% relative intensity cf. base peak)	**theoretical**	**observed**
**1**	2-isopropyl-4,5-dimethyl-3-oxazoline	954, 964	1244, 1253	98, 43 (44), 71 (39), 97 (26), 56 (22), 41 (16), 42 (13), 82 (9), 39 (8), 140 (tr), **141** (tr)	142.1226	142.1230, 142.1231
**2**	2-isobutyl-4,5-dimethyl-3-oxazoline	1054, 1057	1354, 1356	98, 71 (39), 43 (31), 99 (24), 42 (13), 68 (13), 41 (11), 114 (7), 39 (7), 154 (tr), **155** (tr)	156.1383	156.1386, 156.1387
**3**	2-*sec*-butyl-4,5-dimethyl-3-oxazoline	1057, 1067	1354, 1363	98, 71 (33), 43 (29), 97 (14), 70 (13), 42 (12), 41 (11), 55 (10), 99 (8), 154 (tr), **155** (tr)	156.1383	156.1384, 156.1386
**4**	2-benzyl-4,5-dimethyl-3-oxazoline	1416, 1442	2019, 2052	98, 71 (37), 43 (37), 91 (26), 97 (26), 65 (9), 77 (8), 103 (7), 99 (6), 187 (tr), **189** (tr)	190.1226	190.1237, 190.1236
**5**	2-isopropyl-3-oxazoline	859	1245	70, 43 (60), 42 (58), 41 (47), 71 (34), 69 (24), 39 (22), 56 (21), 98 (15), 112 (2), **113** (2)	114.0913	114.0913
**6**	2-isobutyl-3-oxazoline	958	1352	70, 42 (34), 41 (26), 69 (25), 71 (24), 43 (22), 85 (18), 54 (16), 39 (13), 126 (1), **127** (tr)	128.1070	128.1070
**7**	2-*sec*-butyl-3-oxazoline	968	1334	70, 71 (74), 72 (64), 43 (58), 41 (55), 42 (51), 98 (48), 29 (27), 39 (23), 126 (1), **127** (tr)	128.1070	128.1071
**8**	2-benzyl-3-oxazoline	1351	2026	91, 92 (62), 70 (41), 131 (17), 65 (16), 77 (8), 104 (8), 51 (8), 132 (8), 160 (2), **161** (5)	162.0913	162.0924
**9**	2-isopropyl-5-methyl-3-oxazoline	889, 891	1197, 1200	84, 56 (49), 57 (46), 112 (40), 83 (28), 41 (25), 70 (18), 68 (17), 43 (16), 126 (2), **127** (3)	128.1070	128.1070, 128.1070
**10**	2-isobutyl-5-methyl-3-oxazoline	982, 984	1306, 1316	84, 57 (35), 41 (22), 43 (21), 54 (20), 56 (17), 85 (13), 39 (12), 82 (11), 140 (1), **141** (tr)	142.1226	142.1223, 142.1225
**11**	2-*sec*-butyl-5-methyl-3-oxazoline	994, 998	1306, 1307	84, 56 (57), 57 (50), 112 (40), 70 (33), 41 (30), 85 (28), 29 (20), 68 (19), 140 (1), **141** (1)	142.1226	142.1228, 142.1225
**12**	2-benzyl-5-methyl-3-oxazoline	1370, 1380	1972, 1979	84, 91 (64), 92 (38), 57 (35), 65 (13), 131 (12), 104 (10), 77 (9), 103 (8), 174 (tr), **175** (3)	176.1070	176.1073, 176.1071
**13**	2-isopropyl-4-methyl-3-oxazoline	924	1241	84, 83 (32), 56 (19), 57 (18), 41 (10), 42 (10), 82 (8), 39 (7), 29 (7), 126 (tr), **127** (1)	128.1070	128.1072
**14**	2-isobutyl-4-methyl-3-oxazoline	1026	1375	84, 83 (17), 57 (15), 85 (15), 68 (10), 42 (10), 41 (8), 29 (8), 39 (5), 140 (tr), **141** (tr)	142.1226	142.1230
**15**	2-*sec*-butyl-4-methyl-3-oxazoline	1029	1352	84, 83 (32), 57 (18), 42 (10), 41 (9), 29 (8), 55 (8), 85 (7), 39 (6), 140 (tr), **141** (tr)	142.1226	142.1230
**16**	2-benzyl-4-methyl-3-oxazoline	1394	2001	84, 92 (28), 91 (26), 57 (16), 65 (7), 28 (6), 77 (6), 29 (5), 85 (5), 174 (tr), **175** (tr)	176.1070	176.1073
**17a**	2-isopropyl-5-ethyl-4-methyl-3-oxazoline	1031, 1044	1301, 1318	112, 57 (39), 85 (26), 111 (19), 41 (12), 42 (12), 56 (12), 43 (11), 84 (9), 154 (tr), **155** (tr)	156.1383	156.1386, 156.1387
**17b**	2-isopropyl-4-ethyl-5-methyl-3-oxazoline	1029, 1040	1284, 1291	112, 43 (53), 56 (39), 111 (18), 85 (14), 41 (14), 70 (12), 100 (10), 96 (9), 154 (tr), **155** (tr)	156.1383	156.1381, 156.1384
**18a**	2-isobutyl-5-ethyl-4-methyl-3-oxazoline	1133, 1141	1400, 1410	112, 57 (28), 85 (26), 113 (21), 43 (17), 41 (16), 71 (15), 42 (15), 68 (13), 168 (tr), **169** (tr)	170.1539	170.1541, 170.1543
**18b**	2-isobutyl-4-ethyl-5-methyl-3-oxazoline	1130, 1135	1382, 1384	112, 43 (58), 99 (30), 71 (17), 41 (15), 82 (14), 70 (13), 85 (12), 114 (10), 168 (tr), **169** (tr)	170.1539	170.1541, 170.1541,
**19a**	2-*sec*-butyl-5-ethyl-4-methyl-3-oxazoline	1129, 1131, 1142, 1143	1387, 1391, 1405	112, 57 (33), 85 (24), 111 (18), 41 (12), 70 (12), 42 (11), 43 (11), 84 (9), 168 (tr), **169** (tr)	170.1539	170.1541, 170.1543, 170.1544, 170.1545
**19b**	2-*sec*-butyl-4-ethyl-5-methyl-3-oxazoline	1139	1371, 1373, 1378, 1382	112, 43 (46), 70 (29), 111 (19), 85 (14), 41 (14), 96 (13), 56 (12), 55 (11), 168 (tr), **169** (tr)	170.1539	170.1543
**20a**	2-benzyl-5-ethyl-4-methyl-3-oxazoline	1497, 1532	2022, 2076	112, 57 (43), 91 (33), 85 (28), 111 (23), 43 (11), 65 (8), 77 (7), 103 (7), 202 (tr), **203** (tr)	204.1383	204.1387, 204.1385
**20b**	2-benzyl-4-ethyl-5-methyl-3-oxazoline	1495, 1523	1999, 2034	112, 43 (71), 91 (27), 111 (26), 70 (15), 85 (14), 77 (9), 103 (9), 65 (8), 202 (tr), **203** (tr)	204.1383	204.1385, 204.1385

a Where multiple stereoisomers are reported, just the fragmentation pattern of the largest isomer is provided; the first number is the base peak; the molecular ion **M** ^**+**^ is in bold type; tr = trace <0.5%.

b The protonated molecular mass, observed by QToF CI mass spectrometry, matched the theoretical values (±10 ppm) predicted from the chemical formula. Depending on the number of chiral centers in the compound, more than one stereoisomer was observed, supported by very similar, if not identical, mass spectra, LRI values, and protonated exact mass. For **17**–**20**, structural isomers were also observed, supported by pairs of mass spectra differing in the intensity of *m/z*43 and 57.

**Figure 3:**
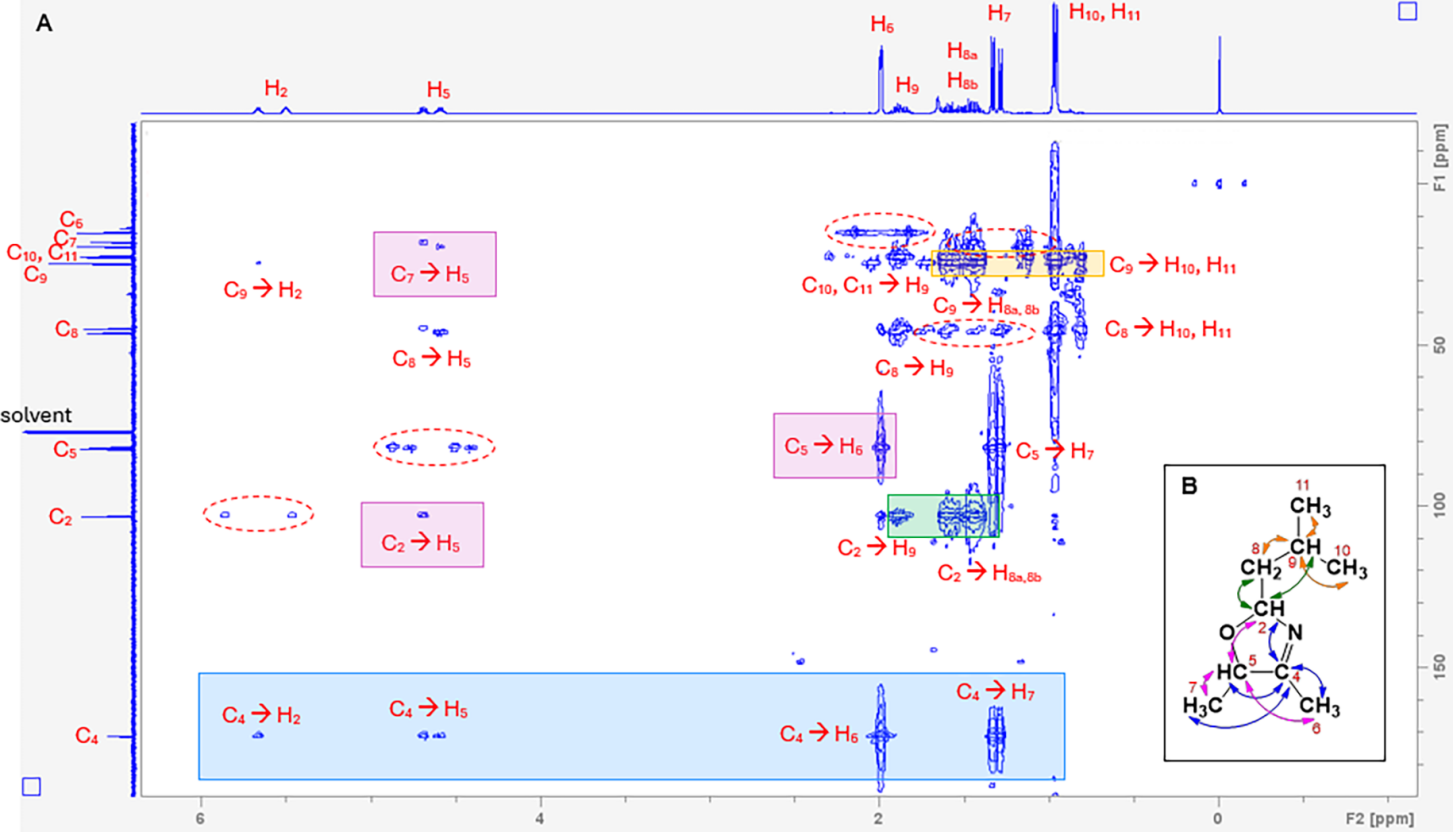
HMBC NMR spectrum (A) that confirms the annotated structure (B) of 2-isobutyl-4,5-dimethyl-3-oxazoline. The signals along the F1 and F2 axes (^13^C and ^1^H spectra, respectively) are assigned according to the atom numbers displayed in (B). The most important cross-peaks, showing the arrangement of atoms key to 3-oxazolines, have been highlighted in colored boxes, with the signals within pink boxes correlating to the pink arrows in (B), and likewise for blue, green, and orange. Each cross-peak indicates that the horizontally and vertically aligned carbon and hydrogen atoms (annotated in red, connected by an arrow) are separated by either two or three bonds within the molecule (H–C–C or H–C–C–C, respectively), confirming the structure in (B). To minimize cluttering of the figure, not all of the cross-peaks have been annotated. The dashed lines circling cross-peak pairs, aligned with a carbon signal but evenly spaced on either side of a hydrogen alignment, indicate that the aligned carbon and hydrogen atoms are directly connected, separated by just one bond (H–C). These signals are artifacts of an HMBC spectrum, therefore are not observed for all atoms; however, the relevant signals can be observed in the HSQC spectrum (Figure S2.9).

The odors of compounds **1**–**4** were characterized by five assessors using gas chromatography–olfactometry (Table 3). The words varied quite considerably, but they were described most frequently as green, cardboard, and musty, although the descriptors from assessor 3 were close to the corresponding Strecker aldehydes.

**Table 3:** GC–O Analysis of Synthesized 3-Oxazolines **1**–4^a^ ^b^

compound	assessor 1	assessor 2	assessor 3	assessor 4	assessor 5
**1a** ^a^	grassy, musty	cardboard	dark chocolate, cocoa powder	odd, vegetable, cardboard	green, drain-ish
**1b**	strange, vegetable	cardboard	floral, chocolate	herbal, medicinal	green, drain-ish
**2**	musty, cocoa	x^b^	chocolate	green, aldehyde-ish	green, minty
**3a**	grassy, musty	powder, makeup	green, waxy, earthy	cardboardy, musty	soil, pyrazine-like
**3b**	green, strange	floral	pink sweets, linalool-like	herbal	x
**4a**	x	x	x	x	x
**4b**	x	x	x	x	x

a a and b refer to two isomers of each compound, which were separable by GC-FID; however for compound **2**, the isomers could not be completely separated, and most assessors could only detect one odor.

b x indicates where an odor could not be detected.

### Identification of 4,5-Dimethyl-3-oxazolines (**1**–**4**) in Cocoa Products

The presence of these 4,5-dimethyl-3-oxazolines was then investigated in cocoa and chocolate. Ground, roasted cacao nibs were subjected to SAFE distillation, and the concentrated aroma extract was analyzed by GC–MS. Using the synthesized 4,5-dimethyl-3-oxazolines as standards, 2-isopropyl-, 2-isobutyl-, 2-*sec-*butyl-, and 2-benzyl-4,5-dimethyl-3-oxazoline (**1**–**4**) were identified in cocoa for the first time. This identification was supported by the matching of LRI values on both the nonpolar and polar columns, identical exact masses by CI ± 10 ppm, and the same EI mass spectrum (Figure 4). In the case of 2-benzyl-4,5-dimethyl-3-oxazoline (**4**), only a very low signal was found; therefore, selected key *m/z* were used for mass spectrum confirmation. However, in the case of **1**–**3**, the signals detected were larger, and more accurate mass spectra were obtained. Due to complexity of the 1D-GC-MS chromatogram, the SAFE extract was also analyzed by 2D-GC–MS for better separation of the compounds. Again, comparison with the synthesized standards allowed the identification of the same 4,5-dimethyl-3-oxazolines (**1**–**4**) on the basis of matching retention times and mass spectra (Figure 5).

**Figure 4:**
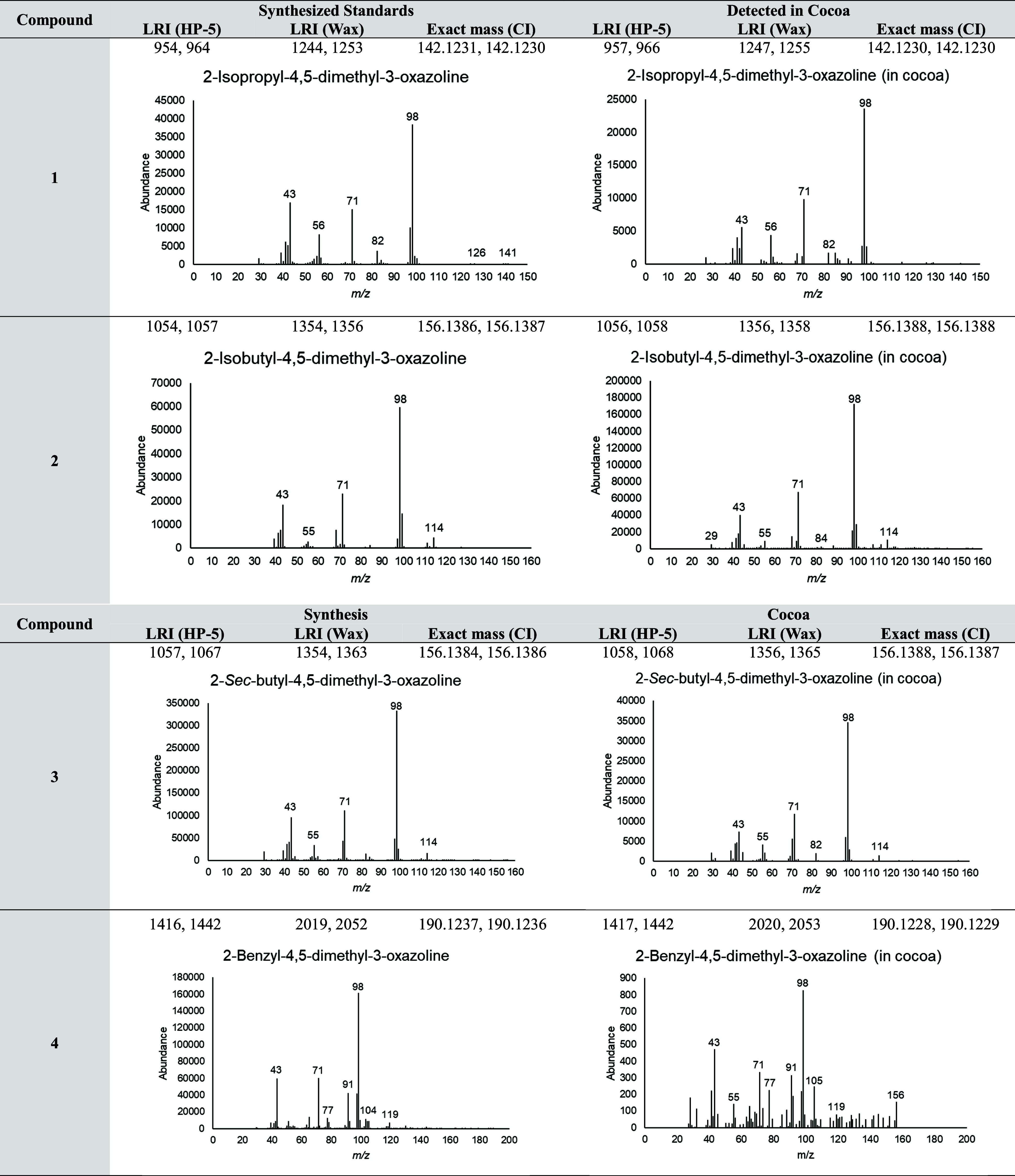
GC–MS data supporting the identification of synthesized 3-oxazolines in cocoa. Mass spectrum provided is from the larger of the two peaks, both of which have similar fragmentation patterns.

**Figure 5:**
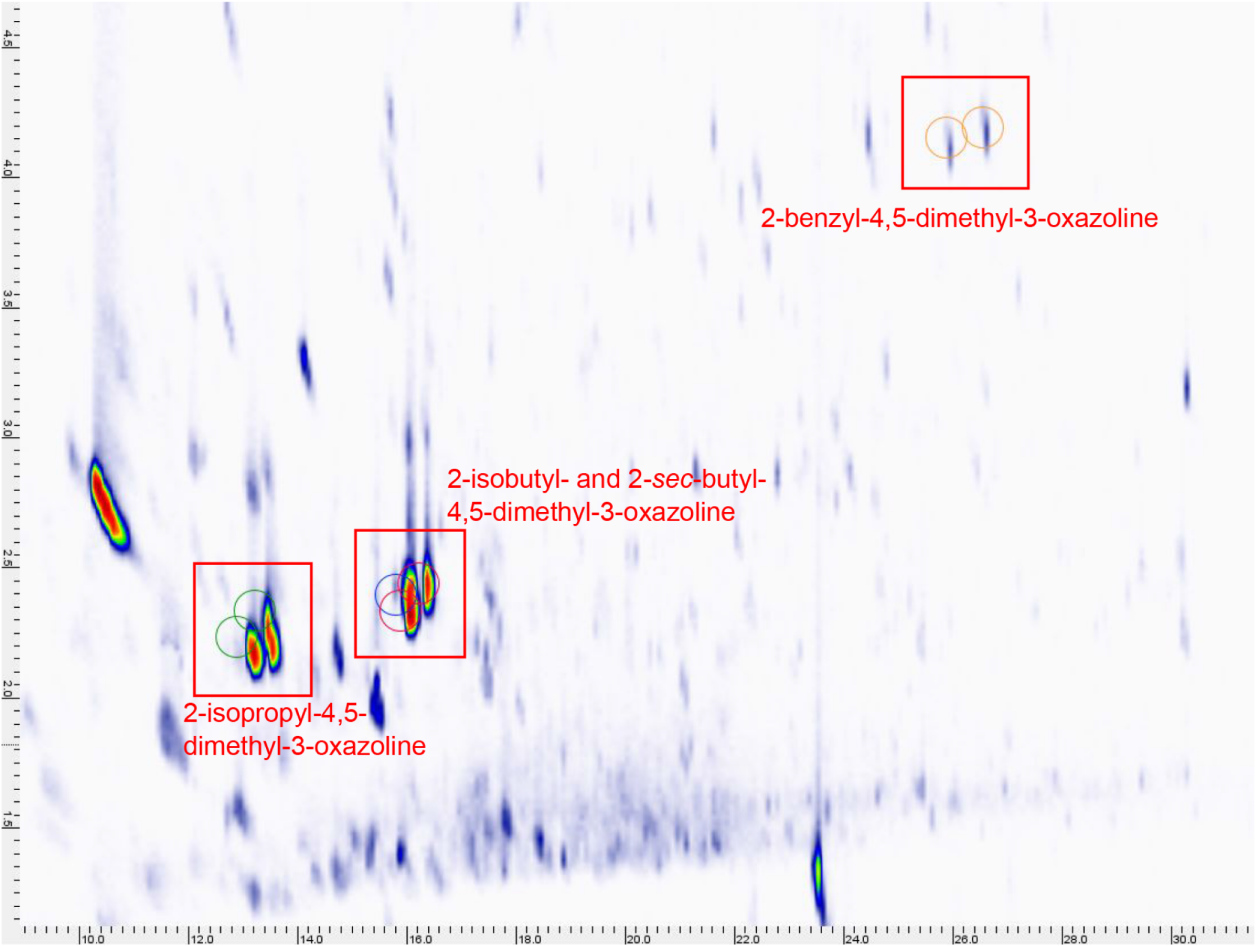
2D-GC–MS analysis of roasted cacao nibs, extracted by SAFE. The synthesized 4,5-dimethyl-3-oxazoline standards **1**–**4** were each run separately and used to create a template (indicated by the green, blue, red, and orange circles, respectively) to show the expected retention times of 4,5-dimethyl-3-oxazolines. This image was created by extracting ion 98 from the chromatogram of the SAFE extract and overlaying with the template. Compounds in cocoa, shown here as blobs, were found close to or within the template regions, and possessed the same mass spectrum as the synthesized standards, indicating the presence of these 4,5-dimethyl-3-oxazolines in cocoa for the first time.

For additional confirmation of the presence of these compounds in cocoa, the synthesized and purified 4,5-dimethyl-3-oxazolines were spiked into the cacao nib SAFE extract and analyzed by 1D-GC–MS. The peaks attributed to 3-oxazolines were observed to increase, confirming that **1**–**3** were indeed present. This also allowed the peaks for 2-isobutyl- and 2-*sec*-butyl-4,5-dimethyl-3-oxazoline to be separated, which are previously undifferentiable due to their similar LRI values (Figure S3).

To search for 3-oxazolines in other cocoa products, aroma extracts of cocoa liquor, dark chocolate, and milk chocolate were obtained by SAFE distillation. Compounds **2** and **3** were consistently identified in all of the samples by 1D-GC–MS; however, 2D-GC–MS analysis enabled confident identification of **1** (in milk chocolate) and **4** (in cacao nibs, cocoa liquor, and milk chocolate) (Figure S4). For dark chocolate, which was not analyzed by 2D-GC–MS, **1** and **4** were tentatively identified by 1D-GC–MS (Table 4). Further work may carry out quantification of these compounds to investigate the effects of cocoa processing.

**Table 4:**
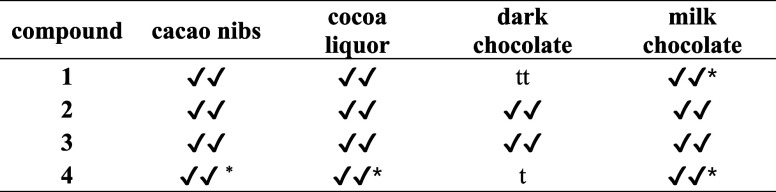
3-Oxazolines Identified in Different Cocoa Products

### Synthesis of Other 3-Oxazolines

In this study, we also speculated about the possibility of 3-oxazolines derived from other α-dicarbonyl compounds being present in cocoa. We synthesized 4,5-unsubstituted-3-oxazolines, 5-methyl-3-oxazolines, 4-methyl-3-oxazolines, and 4/5-ethyl-5/4-methyl-3-oxazolines, theoretically generated from glyoxal, methylglyoxal, and 2,3-pentanedione, respectively (Table 1). Methylglyoxal and 2,3-pentanedione are both asymmetric compounds, so they were proposed to react with amino acids in two orientations to form 3-oxazoline regioisomers, with methylglyoxal forming 5-methyl- and 4-methyl-3-oxazolines (Figure 6), and 2,3-pentanedione forming 5-ethyl-4-methyl- and 4-ethyl-5-methyl-3-oxazolines.

**Figure 6:**
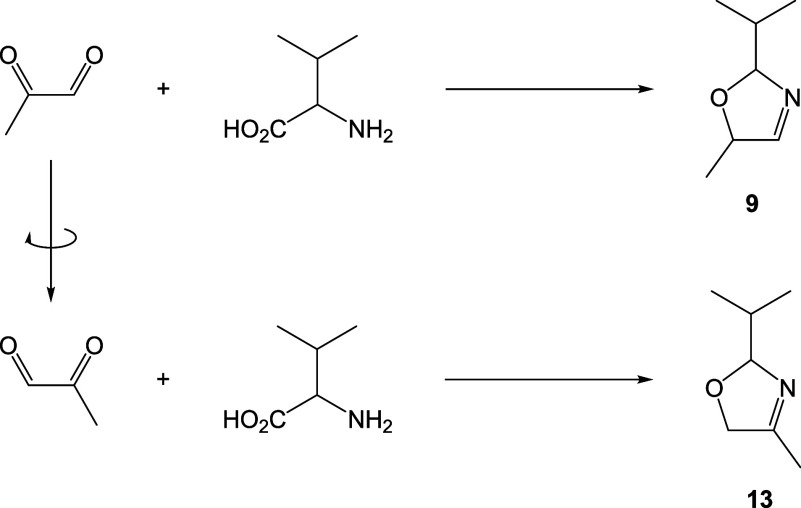
Methylglyoxal is an asymmetrical α-dicarbonyl compound and was proposed to react with amino acids, such as valine, in two conformations to generate 3-oxazoline regioisomers, 2-isopropyl-5-methyl-3-oxazoline and 2-isopropyl-4-methyl-3-oxazoline, in the case of valine (**9** and **13**, respectively). In the same way, 2,3-pentanedione can also react to form the regioisomers, 5-ethyl-4-methyl- and 4-ethyl-5-methyl-3-oxazolines.

Due to the limitations in the availability of reagents, alternative synthetic methods were used (Figure 1), and the purity of the compounds was significantly lower than that of the compounds **1**–**4**, making these compounds very difficult to isolate. The regioisomers arising from methylglyoxal (**9**–**16**) were able to be individually synthesized and identified due to the amino alcohol asymmetry and therefore were given different compound numbers. However, the regioisomers of **17**–**20** were both synthesized from 2,3-pentanedione where there was no regioselective control; therefore, these compounds were labeled with the same compound number but as “a” or “b”. More isomers of **19a/b** were observed due to the extra chiral center of the *sec*-butyl group, possibly leading to the stereoisomers being more spatially different and therefore able to be distinguished by the achiral GC–MS column.

Granvogl et al. previously demonstrated the adaptation of their 3-oxazoline synthetic method by varying the amino alcohol reagent, and this was structurally confirmed by NMR.^1^ Our synthesis was simply an extension of this; therefore, **5**–**16** were tentatively identified on the basis of their mass spectra matching the expected fragmentation pattern (Figure S5), their expected exact mass values being observed by CI mass spectrometry (Table 2), and the fact that analogues prepared by the same synthetic methods had previously been fully characterized by NMR. Compounds **17a/b**–**20a/b** were synthesized by modifying Rizzi’s method, substituting the α-dicarbonyl compound 2,3-butanedione for 2,3-pentanedione. The purification and NMR analysis of regioisomers **17a** and **17b** was attempted, although the isomers could not be separated by column chromatography. The NMR spectrum showed the presence of 2-isopropyl-5-ethyl-4-methyl-3-oxazoline as the major isomer, with the position of the ethyl group on C5 confirmed by the COSY and HMBC experiments. The integrals of the four multiplet signals of H2 and H5, indicative of 3-oxazolines, suggested the presence of the minor regioisomer, 2-isopropyl-4-ethyl-5-methyl-3-oxazoline; however, this could not be confirmed due to the presence of impurities (Figure S6). After ∼5 days of storage of the samples, 2-isopropyl-5-ethyl-4-methyloxazole was observed by GC–MS (confirmed with the NIST Chemistry WebBook^19^), likely caused by oxidation of the synthesized oxazoline, further supporting evidence that the 5-ethyl-4-methyl-3-oxazoline was the major isomer. Use of the same synthetic method, varying just the amino acid, to generate **18a/b**–**20a/b**, as well as the expected mass fragmentation and exact molecular mass being observed, provided enough evidence for us to conclude (albeit tentatively) that 5/4-ethyl-4/5-methyl-3-oxazolines had also been synthesized.

### Tentative Identification of Other 3-Oxazolines in Cocoa

Similarly to the 4,5-dimethyl-3-oxazolines, the retention time and mass spectra of the 4,5-unsubstituted-3-oxazolines, 5/4-methyl-3-oxazolines, and 5/4-ethyl-4/5-methyl-3-oxazolines were also used to search for their presence in cocoa. None of the 4,5-unsubstituted- or 5/4-methyl-3-oxazolines could be found; however, the 5/4-ethyl-4/5-methyl-3-oxazolines were tentatively identified in cacao nibs and cocoa liquor. This identification was tentative due to the low signal observed and coeluting compounds also affecting the mass spectra (Figure 7). Nevertheless, analysis by 2D-GC–MS and GC-QToF CI mass spectrometry was supportive of these identifications (Figure S7). Depending on the size of the signal, it was sometimes not possible to distinguish the regioisomers of each compound and the 2-isobutyl and 2-*sec*-butyl substituents, due to the similarity of the mass spectra and the variation in LRI values between isomers being close to the error margin of the GC–MS. Similarly to 2-benzyl-4,5-dimethyl-3-oxazoline, the signal of 2-benzyl-5/4-ethyl-4/5-methyl-3-oxazoline found was also very low, such that it was not detected by 1D-GC–MS but only by 2D. Since phenylacetaldehyde is a key component of chocolate aroma and was also found to increase significantly upon adding water,^14^ it is surprising that these benzyl 3-oxazolines were found at lower signals relative to the isopropyl and iso/*sec*-butyl substituents. A possible explanation could be that the higher lipophilicity of benzyl 3-oxazolines means that they form adducts in the fat phase of cocoa butter and are less able to be extracted by SAFE; however, this needs to be explored further.

**Figure 7:**
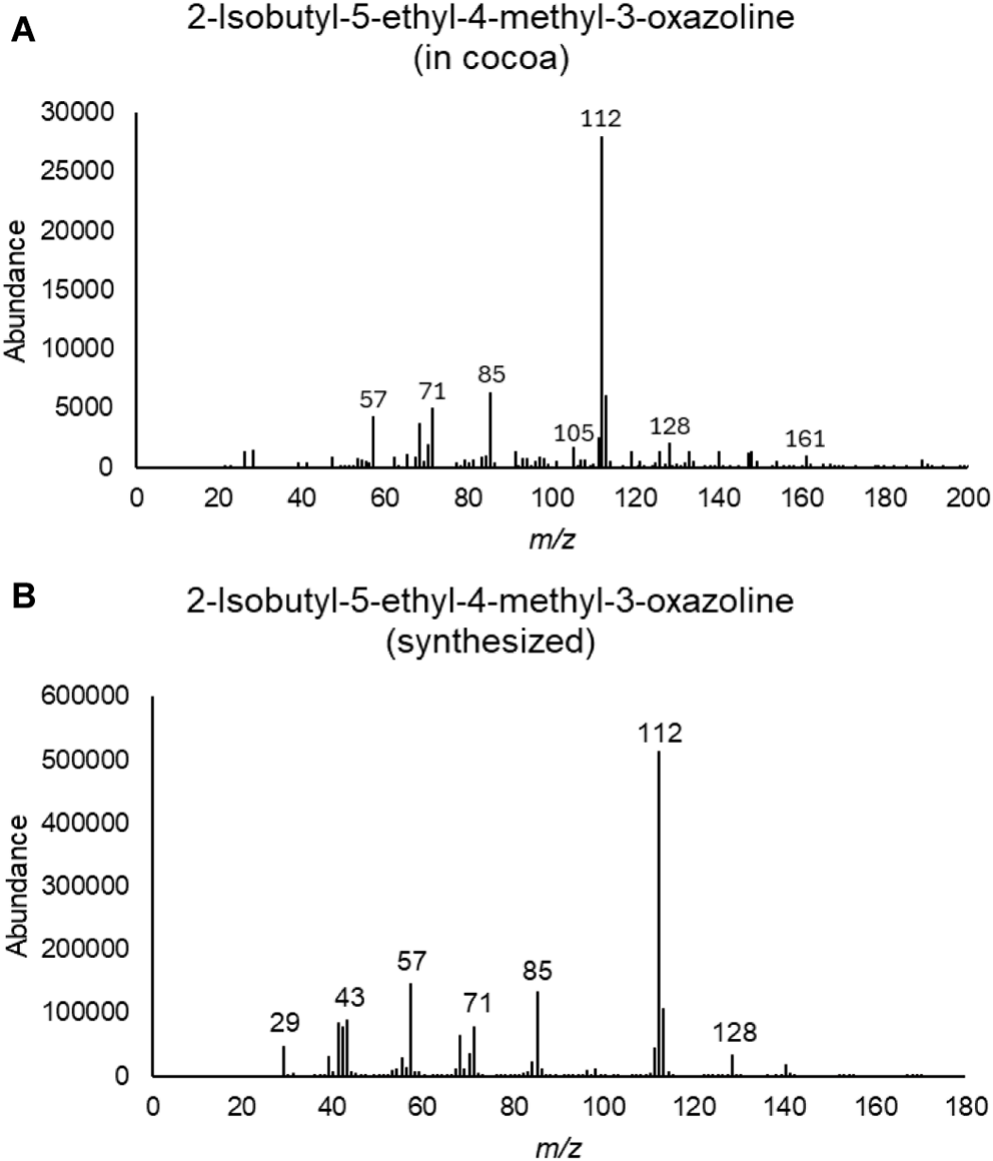
(A) Mass spectrum of the peak identified as 2-isobutyl-5-ethyl-4-methyl-3-oxazoline in cacao nibs. (B) Mass spectrum of synthesized 2-isobutyl-5-ethyl-4-methyl-3-oxazoline.

The identification of just 4,5-dimethyl-3-oxazolines and tentative identification of 5/4-ethyl-4/5-methyl-3-oxazolines in cocoa products suggest that 2,3-butanedione is a major α-dicarbonyl precursor of 3-oxazoline formation, with 2,3-pentanedione also contributing but to a much lesser extent. Not detecting the 4,5-unsubstituted- or 5/4-methyl-3-oxazolines was a surprising result as glyoxal and methylglyoxal are known to be reactive α-dicarbonyl compounds and present in high levels in chocolate,^20,21^ however it is possible that they are depleted by other reactive pathways and are therefore not available for 3-oxazoline formation. Nevertheless, there could still be other types of 3-oxazolines that exist and hydrolyze to form Strecker aldehydes, as only a limited range was synthesized in this study. Long-chain polyhydroxyl α-dicarbonyl compounds, such as glucosone, 1-deoxyglucosone or 3-deoxyglucosone, or even Amadori products, have been proposed as 3-oxazoline precursors, capable of Strecker aldehyde release.^1^

In conclusion, we have synthesized and fully characterized four 4,5-dimethyl-3-oxazolines (**1**–**4**) that we have identified in four cocoa products for the first time. Furthermore, we have synthesized an array of other 3-oxazolines using established methods. Most of these were not detected in the cocoa products, except for the tentative identification of 5/4-ethyl-4/5-methyl-3-oxazolines in cocoa. This is a key step in proving that 3-oxazolines are the precursors in low-moisture foods responsible for the significant release of Strecker aldehydes when water is added.

## Supplementary Material



## Notes

Figure S1: the Strecker degradation of amino acids, initiated by α-dicarbonyl compounds, to form the aroma compounds, Strecker aldehydes; Figure S2: NMR spectra (^1^H, ^13^C, ^1^H–^1^H COSY, ^1^H–^13^C HSQC, and ^1^H–^13^C HMBC) of synthesized 3-oxazolines **1**–**4**; Figure S3: GC–MS analysis of cacao nib SAFE extract, spiked with 2-isobutyl- and 2-*sec*-butyl-4,5-dimethyl-3-oxazoline (**2** and **3**); Figure S4: GC–MS identification of 4,5-dimethyl-3-oxazoline in different cocoa products; Figure S5: EI mass spectra of additionally synthesized 3-oxazolines **5**–**20**;Figure S6: NMR spectra (^1^H, ^13^C, ^1^H–^1^H COSY,^1^ H–^13^C HSQC, and ^1^H–^13^C HMBC) of 2-isopropyl-5-ethyl-4-methyl-3-oxazoline (**17a**); Figure S7: 2D-GC–MS analysis of roasted cacao nibs, extracted by SAFE (PDF)

## Abbreviations

1D-GC–MS: 1-dimensional gas chromatography coupled to mass spectrometry
2D-GC–MS: 2-dimensional gas chromatography coupled to mass spectrometry
AR: analytical reagent
COSY: correlation spectroscopy
GC-FID: gas chromatography coupled to flame ionization detection
GC–O: gas chromatography coupled to olfactometry
GC-QToF: gas chromatography coupled to a quadrupole time-of-flight detector
HMBC: heteronuclear multiple-bond correlation
HSQC: heteronuclear single quantum coherence
MCT: medium-chain triglycerides
NIST: National Institute of Standards and Technology
SAFE: solvent-assisted flavor evaporation
