# Supplementary material for: Synthesis and Identification of 3‑Oxazolines in Cocoa
Source: J Agric Food Chem. 2025 Jun 5;73(24):15259–69. doi: 10.1021/acs.jafc.5c00898 (PMC12186532; doi:10.1021/acs.jafc.5c00898)
Supplement: Supplementary file 1 [file jf5c00898_si_001.pdf]

Supporting Information

Synthesis and Identification of 3-Oxazolines in Cocoa

Heather G. Spooner[1], Dimitris P. Balagiannis[1], Andreas Czepa[2#], Barbara Suess[2], Martine Trotin[2], Paul O'Nion[3], Jane K. Parker[1*]

[1]Department of Food and Nutritional Sciences, University of Reading, Whiteknights, Reading, UK, RG6 6AP

[2]Mondelēz International, Whiteknights Campus, Pepper Lane, Reading, RG6 6LA

[3]Reading Scientific Services Ltd, Whiteknights Campus, Pepper Lane, Reading, RG6 6LA

*Corresponding author: Jane K Parker, j.k.parker@reading.ac.uk Tel + 44 118 378 7455

[#]Current affiliation: tba

Figure S1: The Strecker degradation of amino acids, initiated by α-dicarbonyl compounds, to form the aroma compounds, Strecker aldehydes.

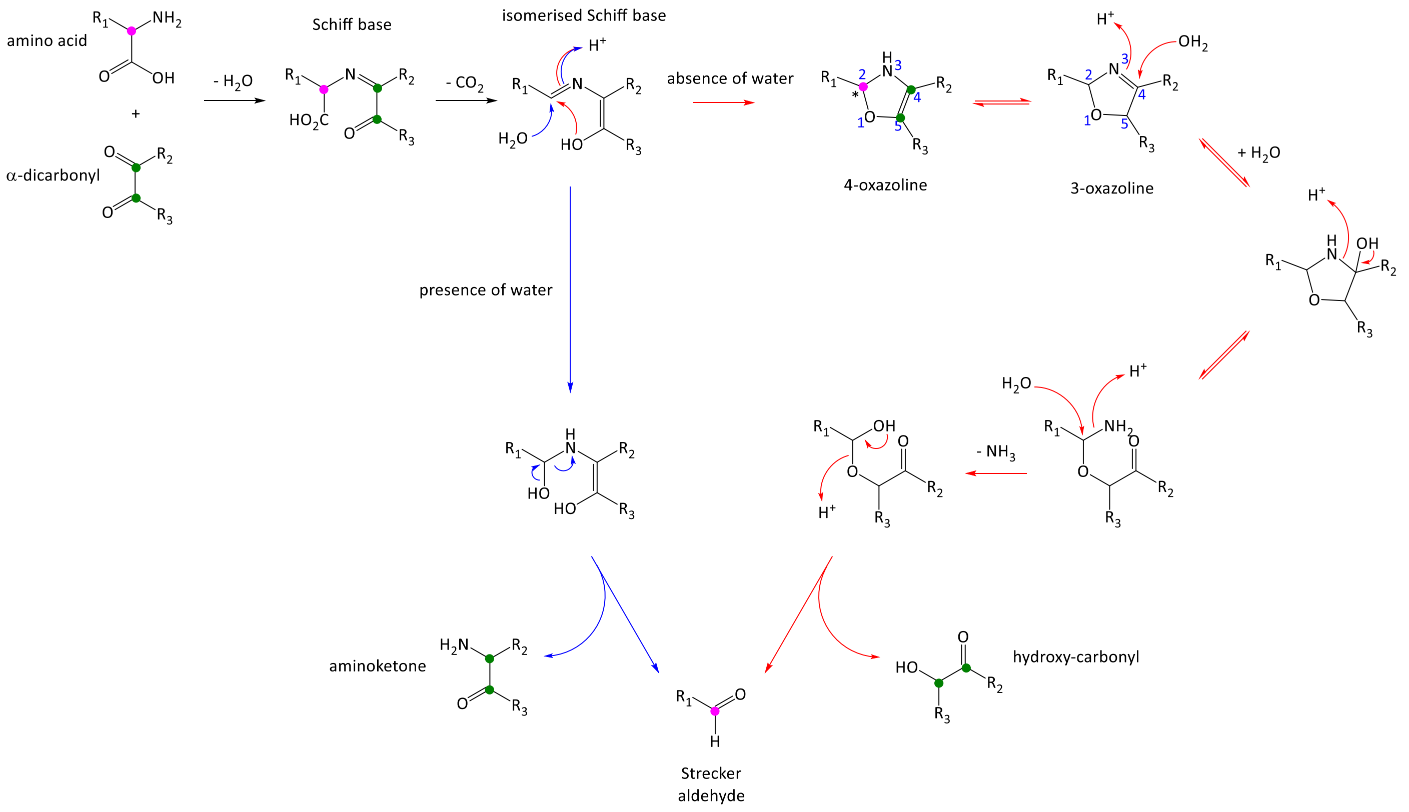

The R groups represent variable side chains, due to the six possible amino acids that can take part in this reaction, and the variety of α-dicarbonyl compounds that can arise via the Maillard reaction. The curly arrows represent movement of electron pairs, and the pink and green dots indicate which carbon atoms are retained throughout the reaction in order to follow molecular rearrangement. Adapted from Granvogl et al.[1]

Blue pathway: the 'conventional' route of Strecker degradation is well-known and occurs in the presence of water.

Red pathway: the route of Strecker degradation that occurs in the absence of water via the formation of oxazoline intermediates, proposed by Granvogl et al.[1] The numbered atoms are to explain the naming of 3- and 4-oxazolines, and the asterisk indicates a newly formed chiral center, which results in the formation of oxazoline stereoisomers.

Figure S2: NMR spectra of synthesized 3-oxazolines. Diastereomeric peaks were integrated together (indicated by ’). Only the key signals are annotated.

Figure S2.1: ^{1}H NMR spectrum of 2-isopropyl-4,5-dimethyl-3-oxazoline.

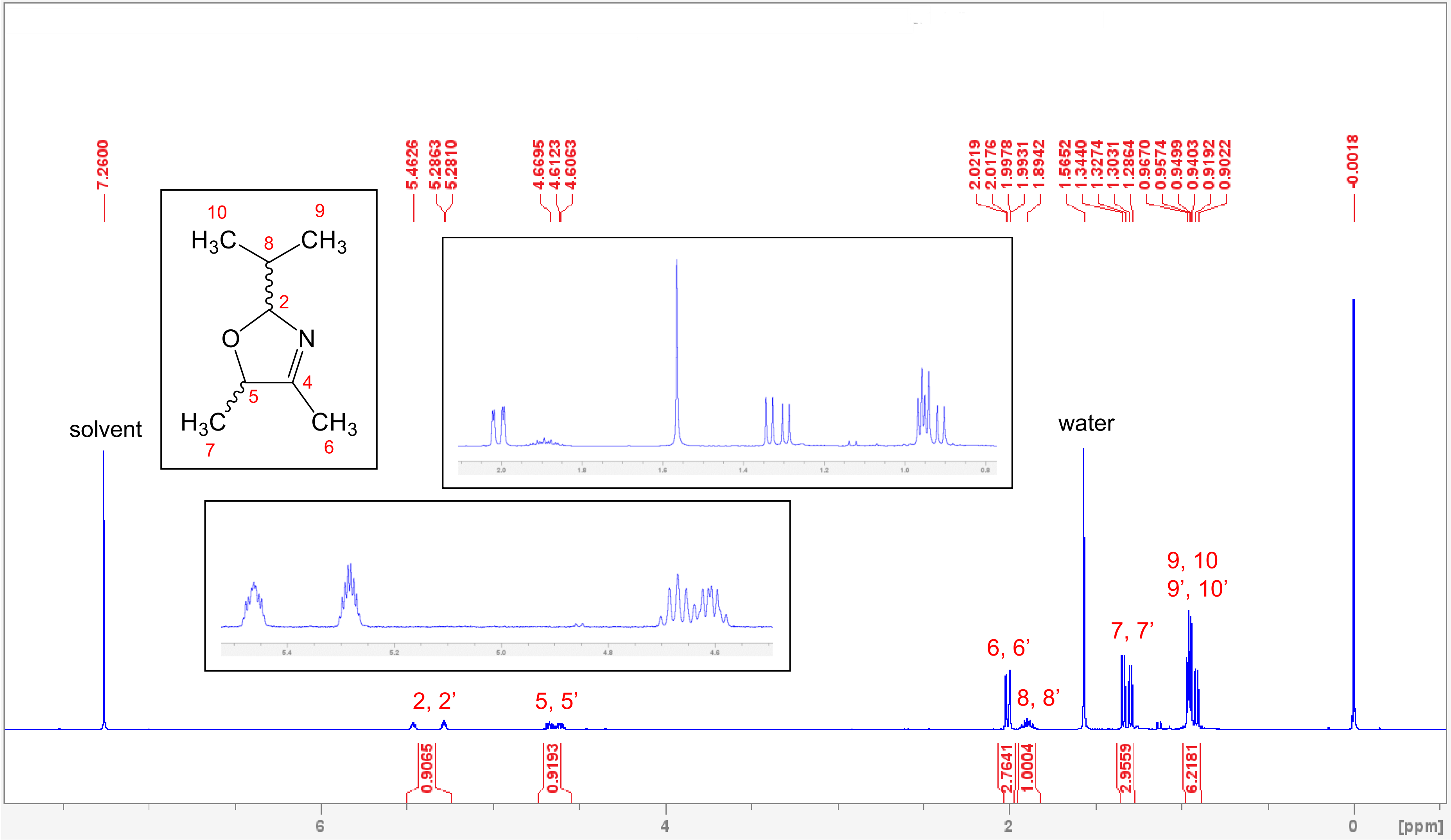

Version 01Jan

Figure S2.2: ^{13}C NMR spectrum of 2-isopropyl-4,5-dimethyl-3-oxazoline.

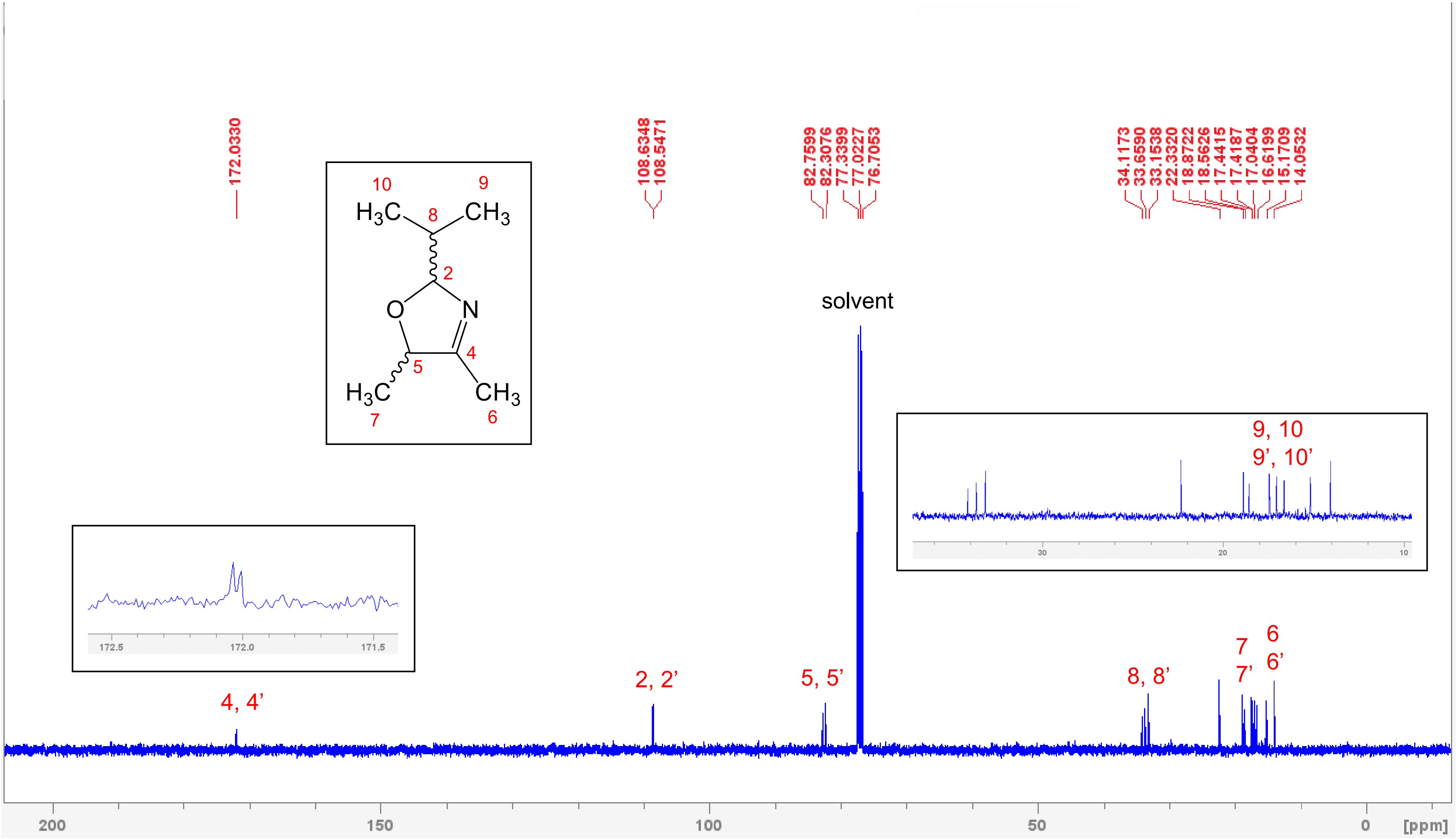

Figure S2.3: $^1H - {^1H}$ COSY NMR spectrum of 2-isopropyl-4,5-dimethyl-3-oxazoline.

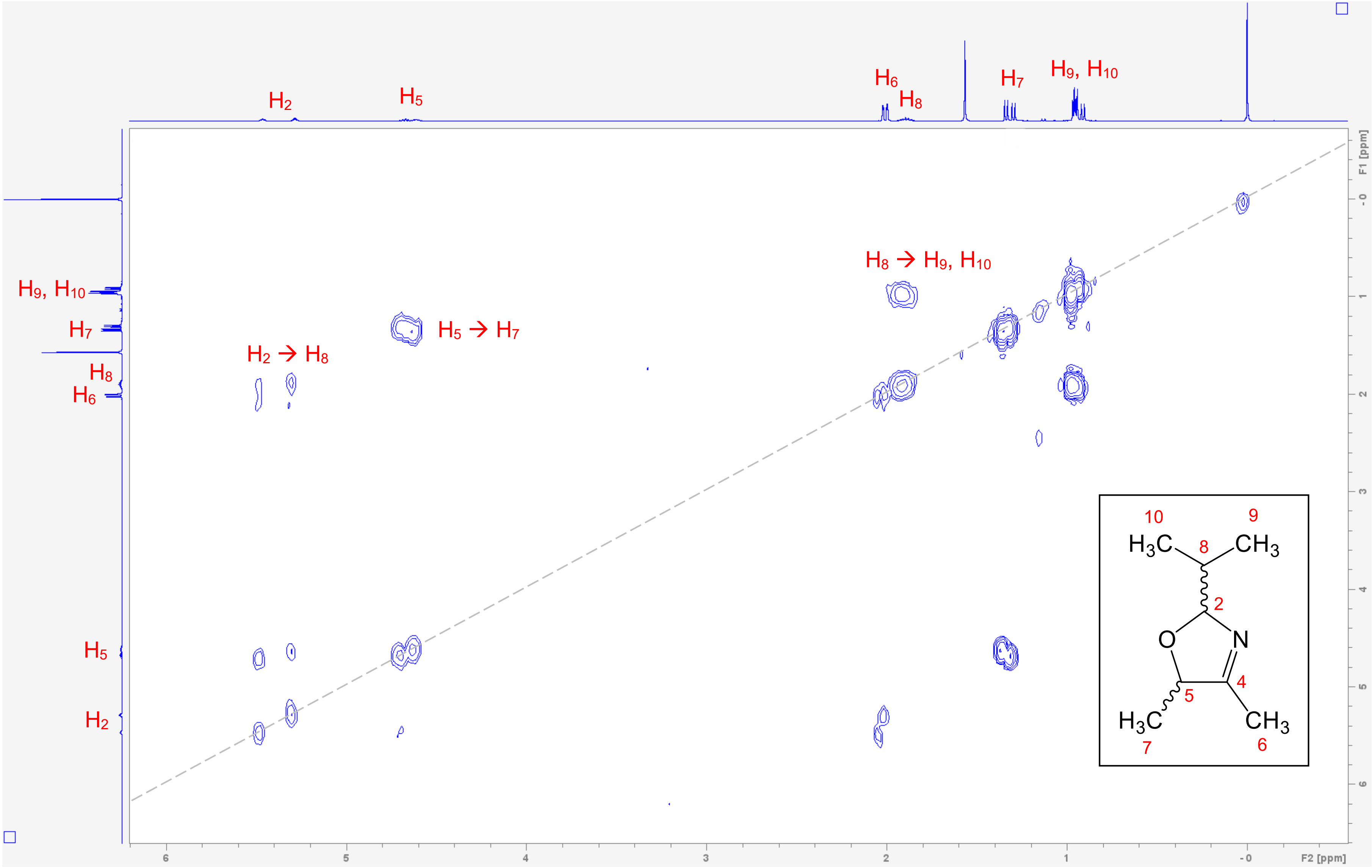

Figure S2.4: ^{1}H – ^{13}C HSQC NMR spectrum of 2-isopropyl-4,5-dimethyl-3-oxazoline.

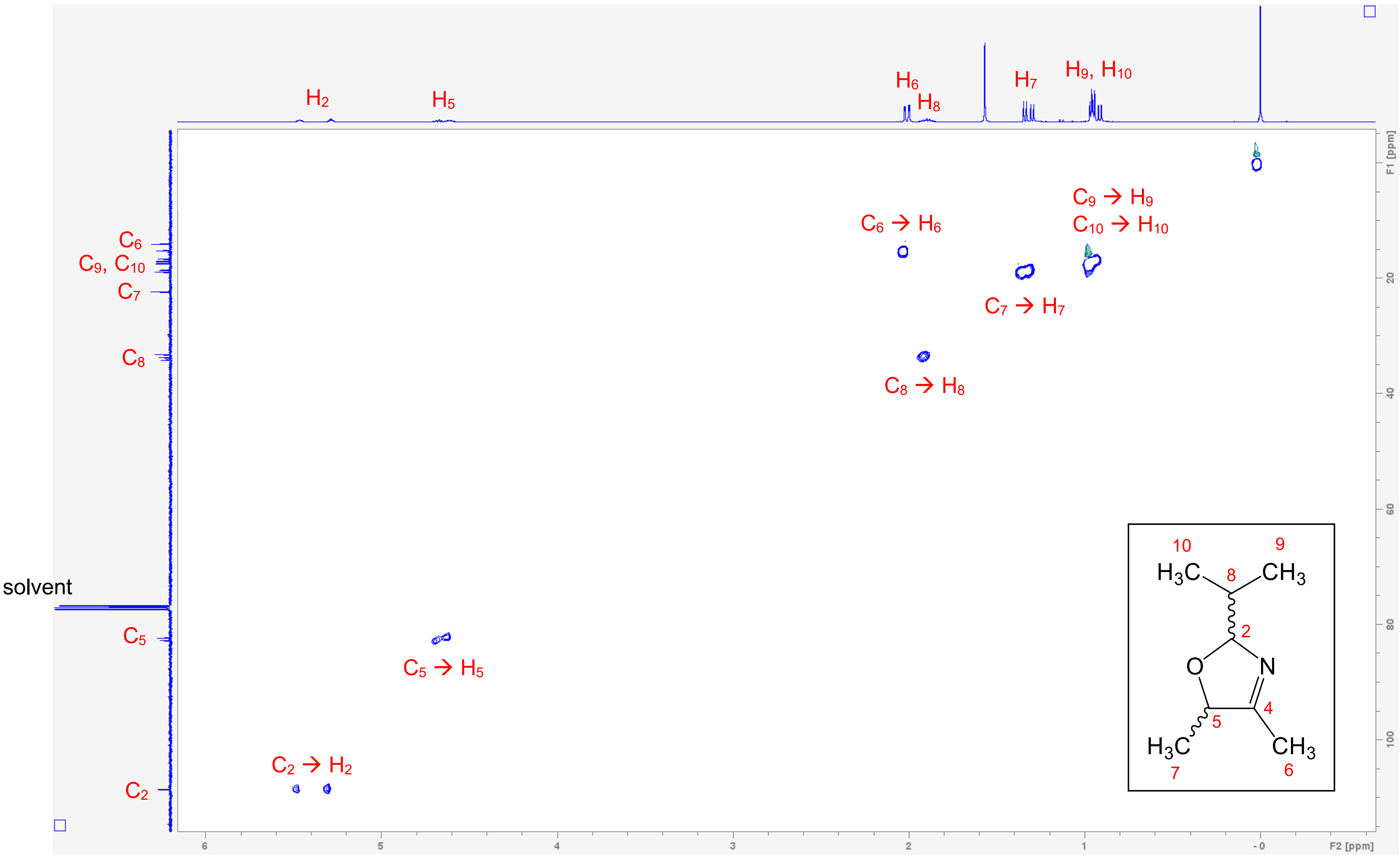

Figure S2.5: ¹H – ¹³C HMBC NMR spectrum of 2-isopropyl-4,5-dimethyl-3-oxazoline.

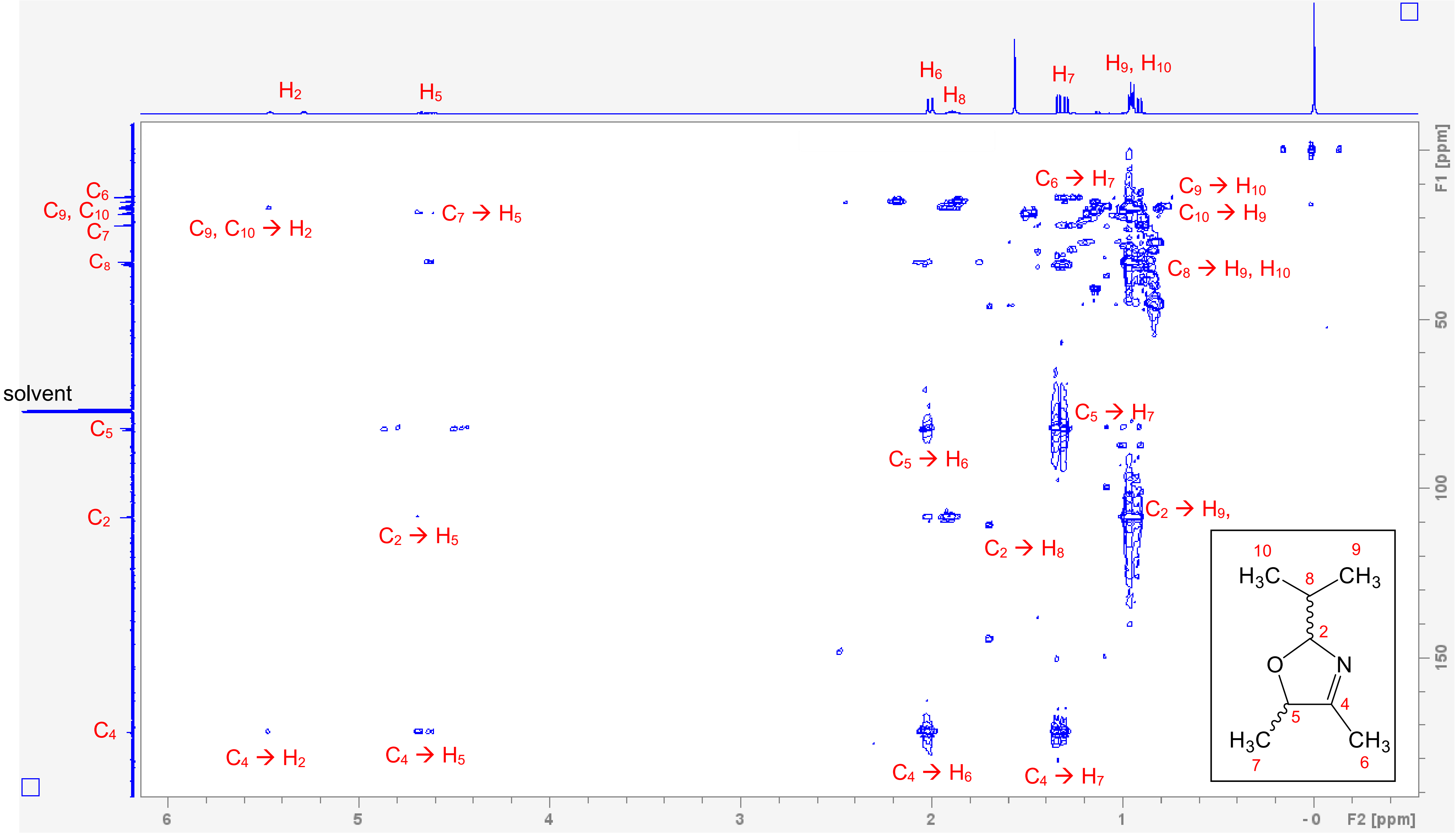

Figure S2.6: ^{1}H NMR spectrum of 2-isobutyl-4,5-dimethyl-3-oxazoline.

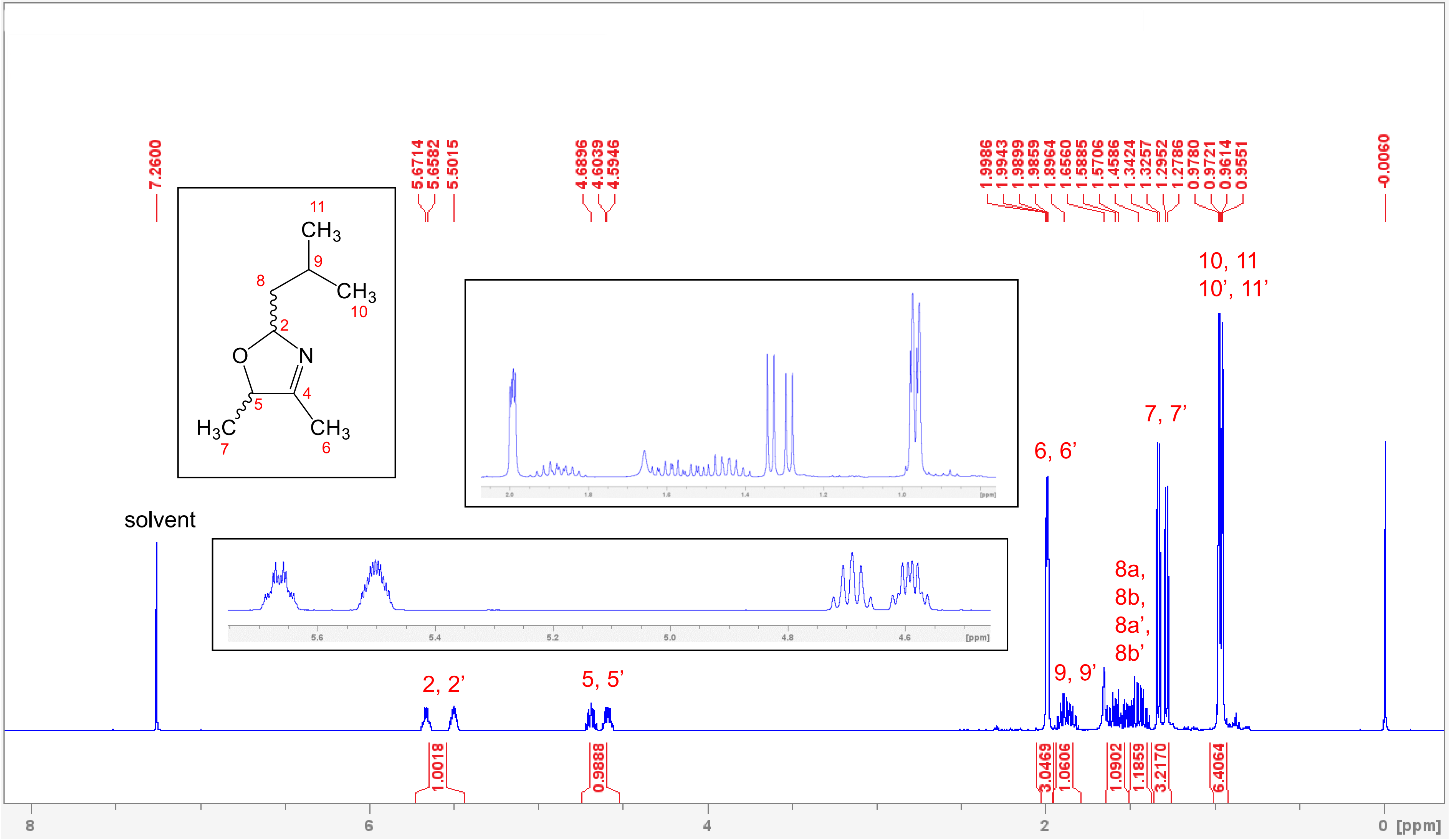

Version 01Jan

Figure S2.7: ^{13}C NMR spectrum of 2-isobutyl-4,5-dimethyl-3-oxazoline.

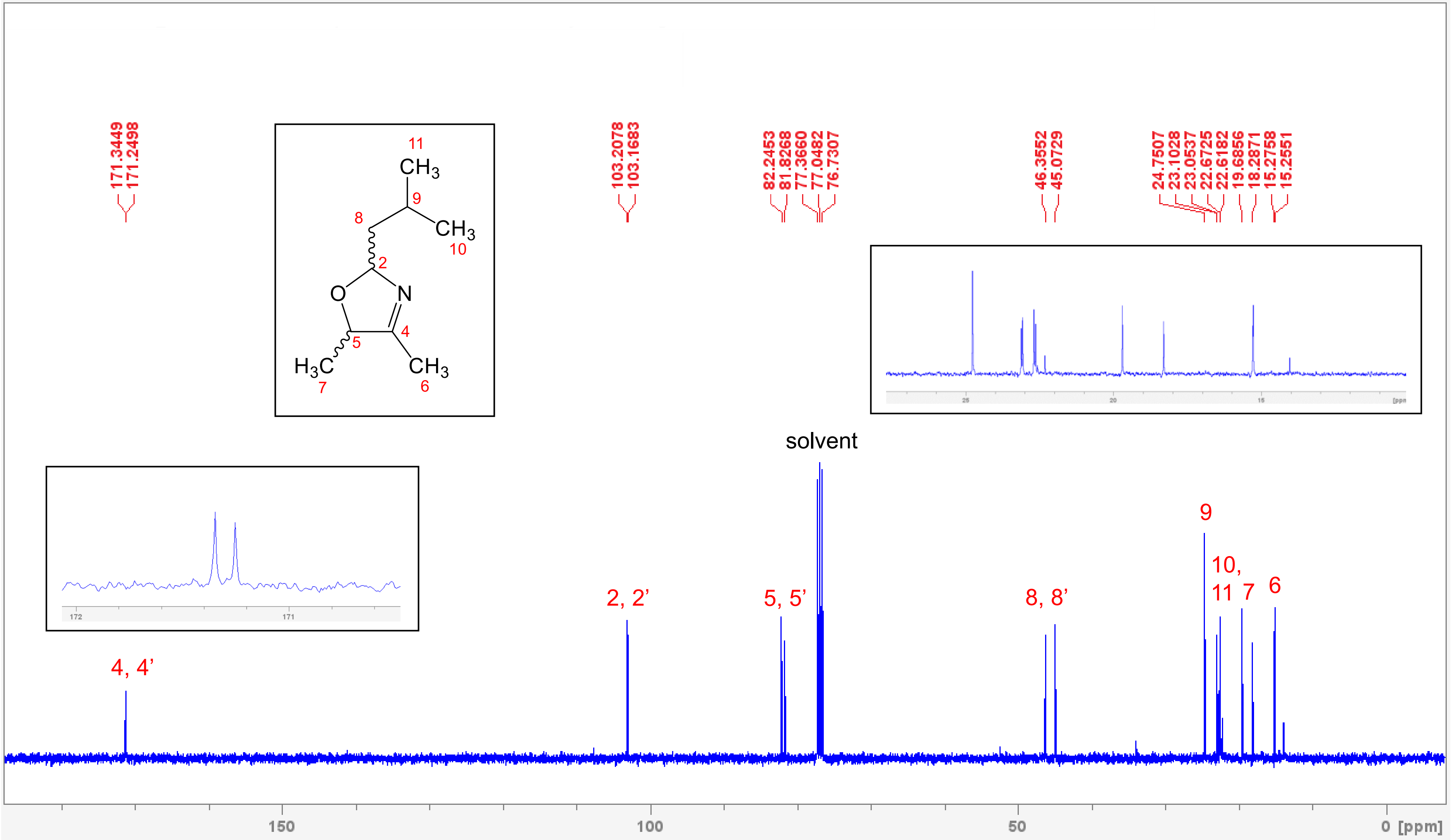

Figure S2.8: ^1H – ^1H COSY NMR spectrum of 2-isobutyl-4,5-dimethyl-3-oxazoline.

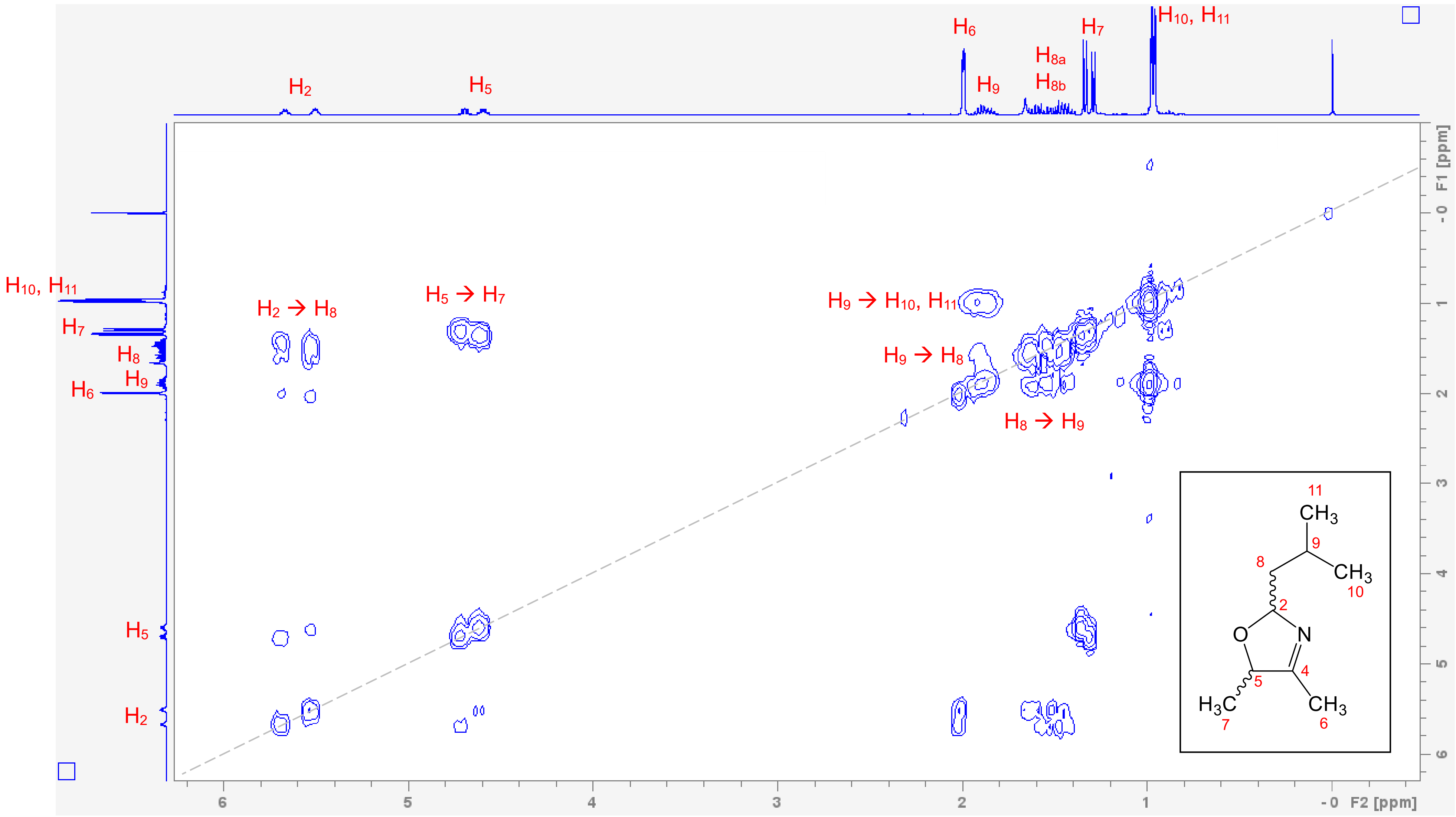

Figure S2.9: $^1H - ^{13}C$ HSQC NMR spectrum of 2-isobutyl-4,5-dimethyl-3-oxazoline.

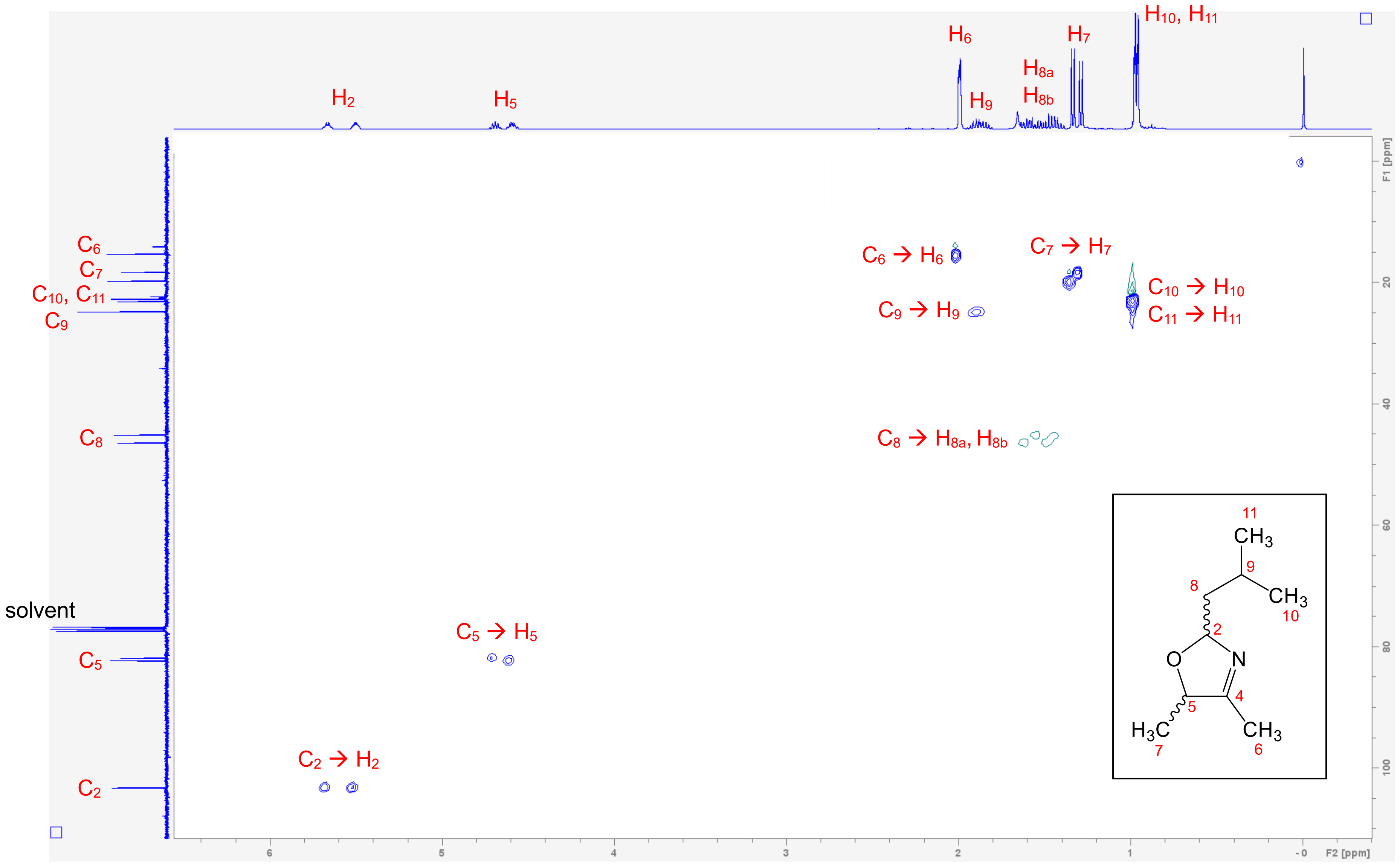

Figure S2.10: ^{1}H – ^{13}C HMBC NMR spectrum of 2-isobutyl-4,5-dimethyl-3-oxazoline.

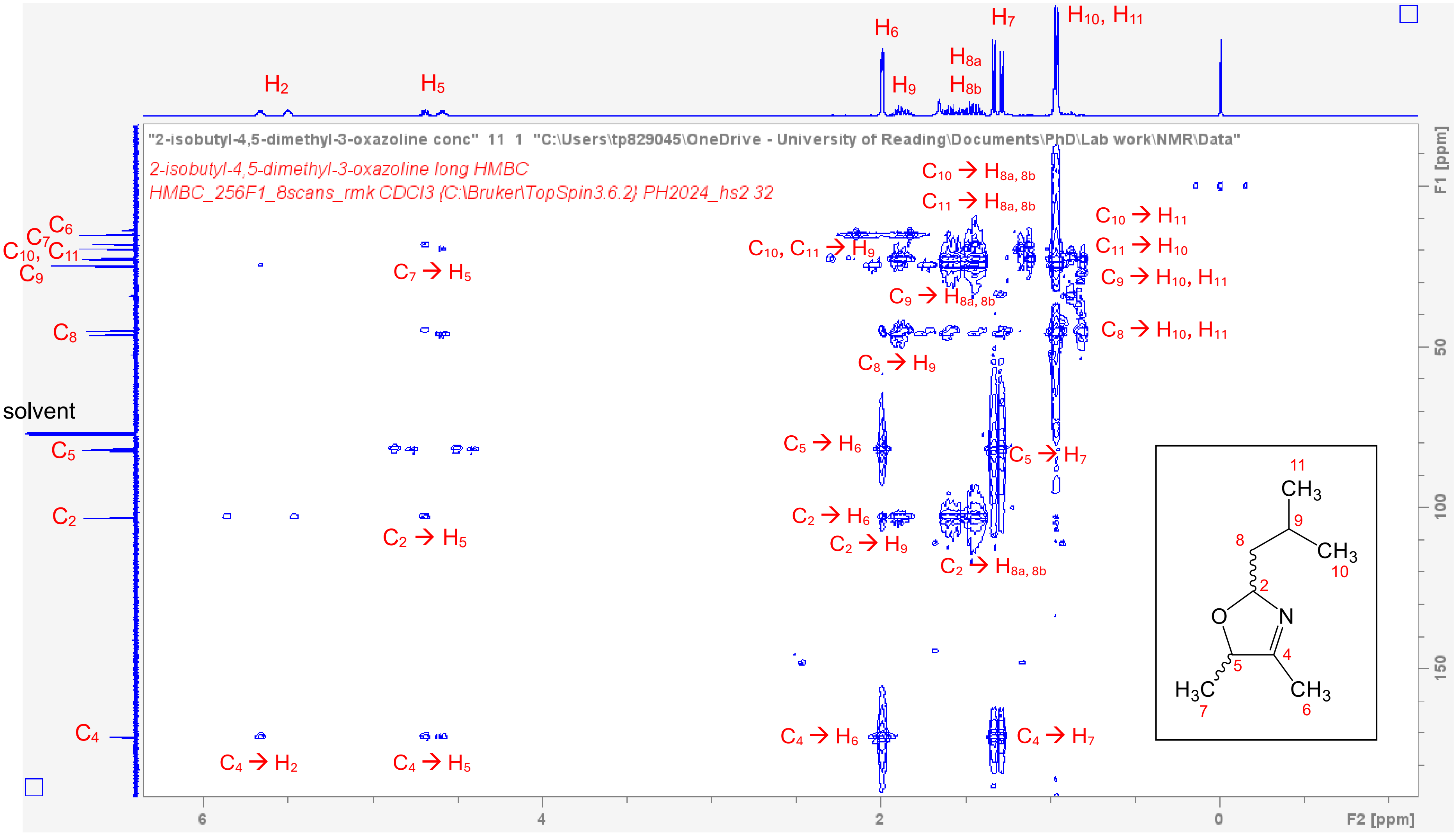

Version 01Jan

Figure S2.11: ^{1}H NMR spectrum of 2-*sec*-butyl-4,5-dimethyl-3-oxazoline. In the close-up images, diastereomeric signals of H2, H5, H6 and H7 were integrated separately to reveal a diastereomeric ratio of 3:2.

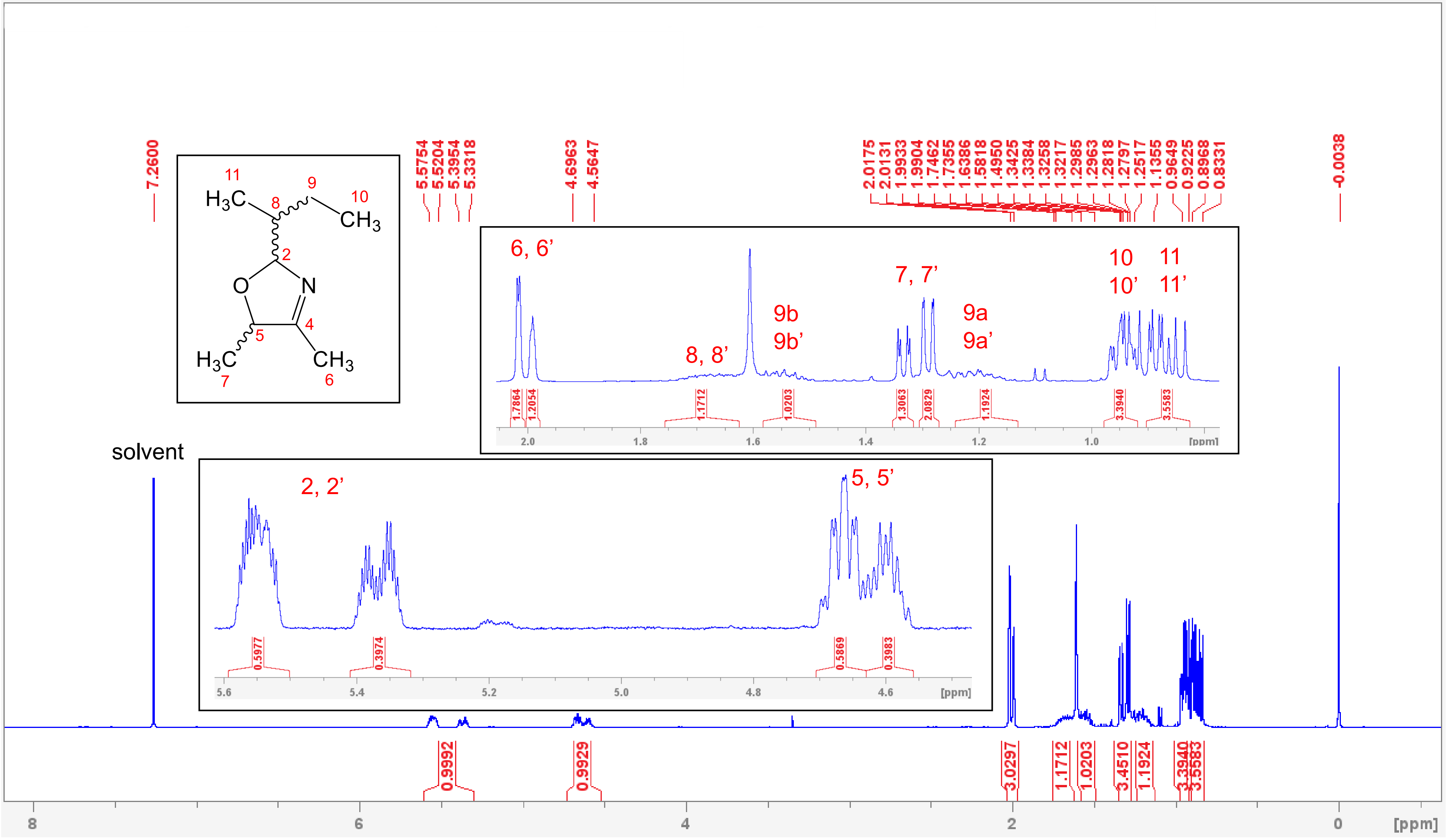

Figure S2.12: ^{13}C NMR spectrum of 2-*sec*-butyl-4,5-dimethyl-3-oxazoline.

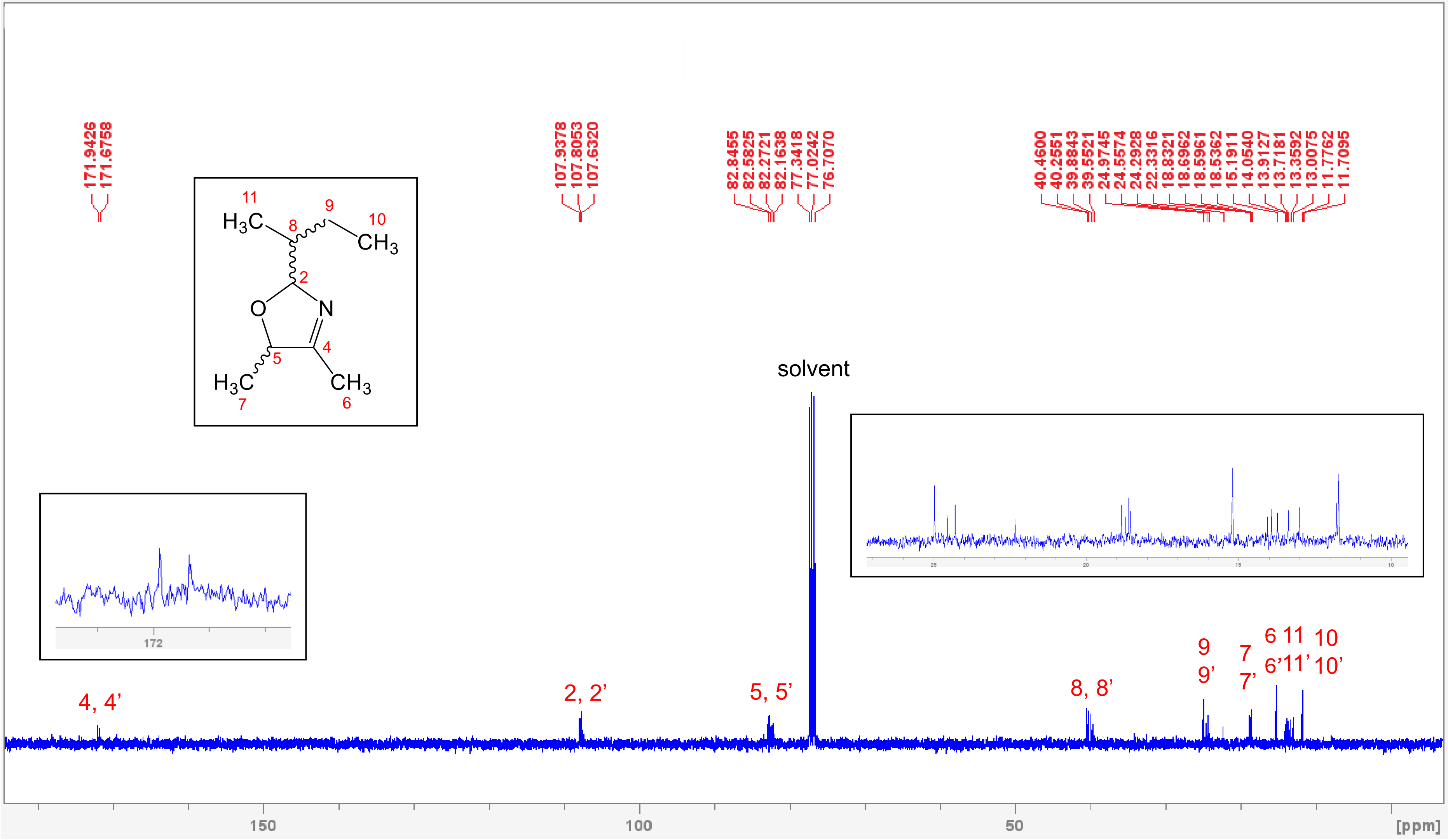

Figure S2.13: ^{1}H – ^{1}H COSY NMR spectrum of 2-*sec*-butyl-4,5-dimethyl-3-oxazoline.

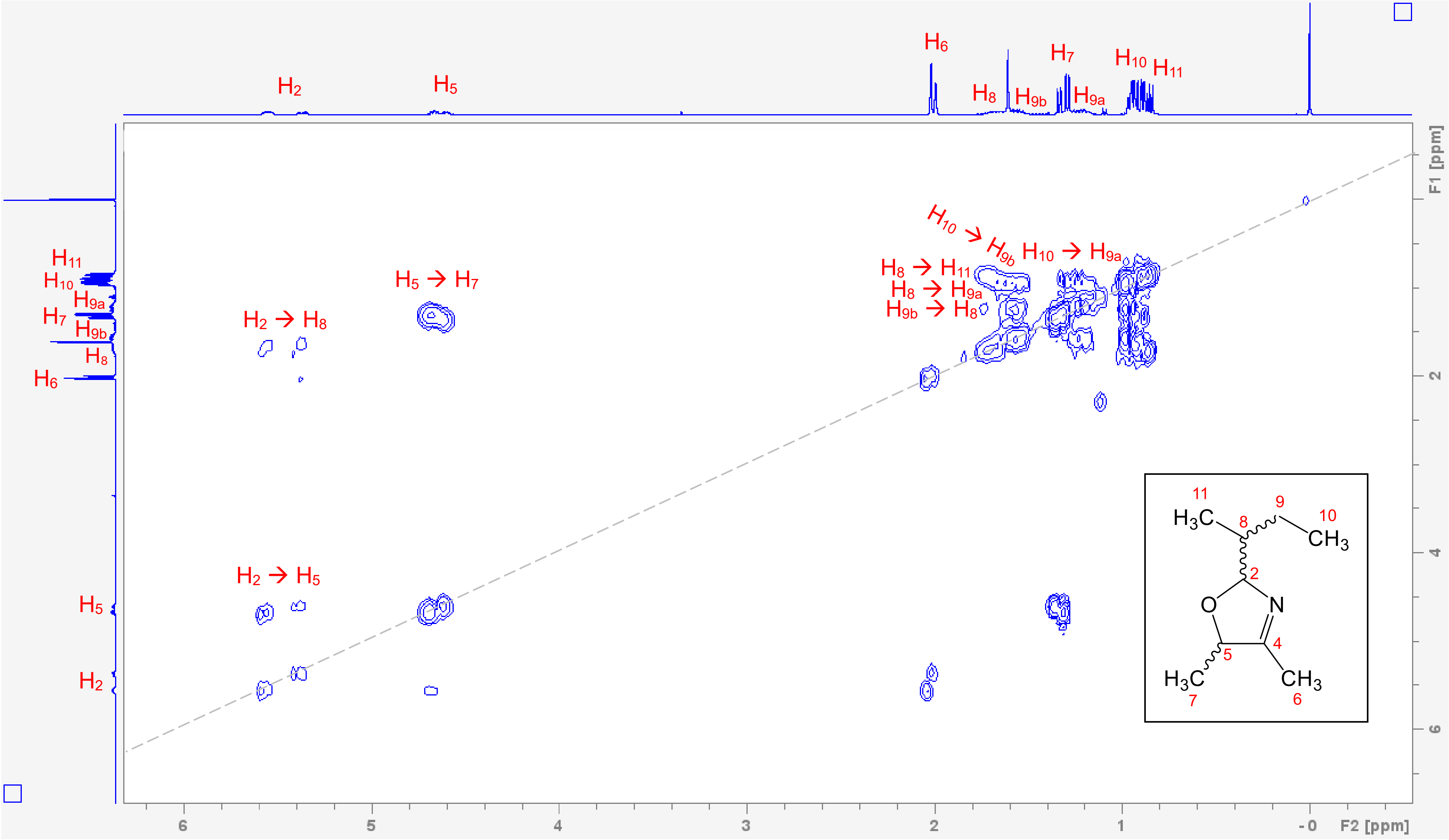

Figure S2.14: ^{1}H – ^{13}C HSQC NMR spectrum of 2-*sec*-butyl-4,5-dimethyl-3-oxazoline.

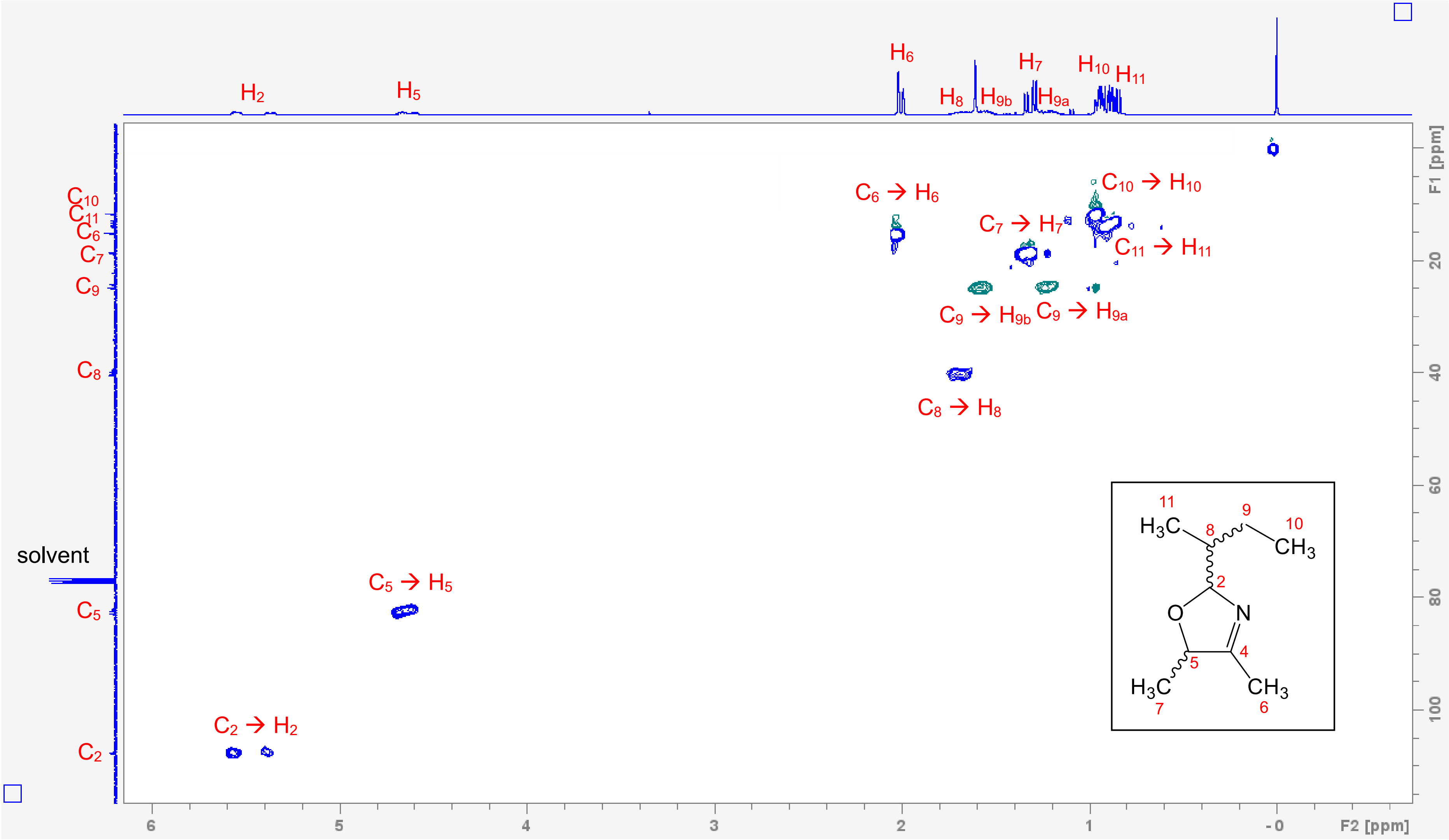

Figure S2.15: ^{1}H – ^{13}C HMBC NMR spectrum of 2-*sec*-butyl-4,5-dimethyl-3-oxazoline.

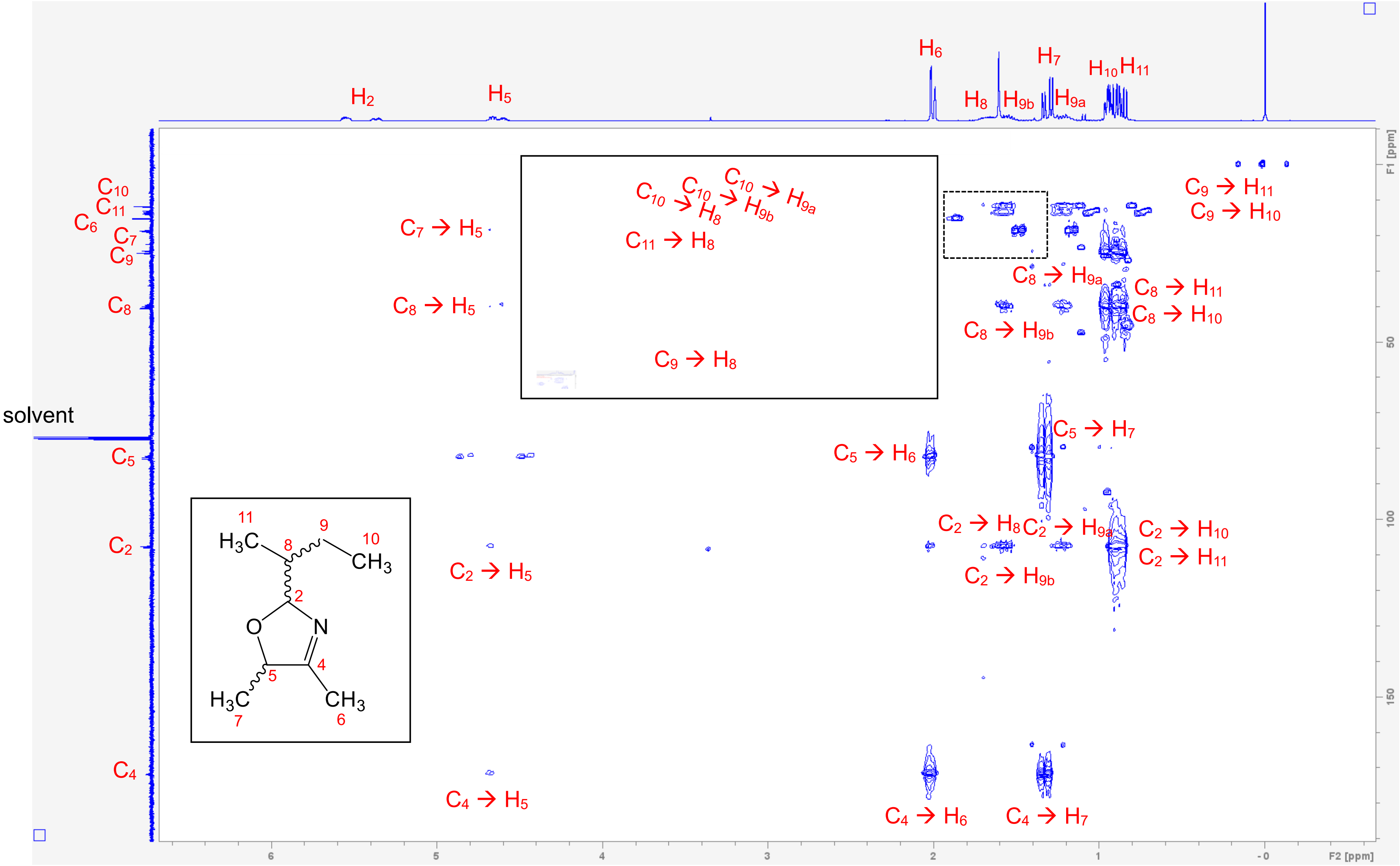

Figure S2.16: ^{1}H NMR spectrum of 2-benzyl-4,5-dimethyl-3-oxazoline. TMP = tetramethylpyrazine (impurity).

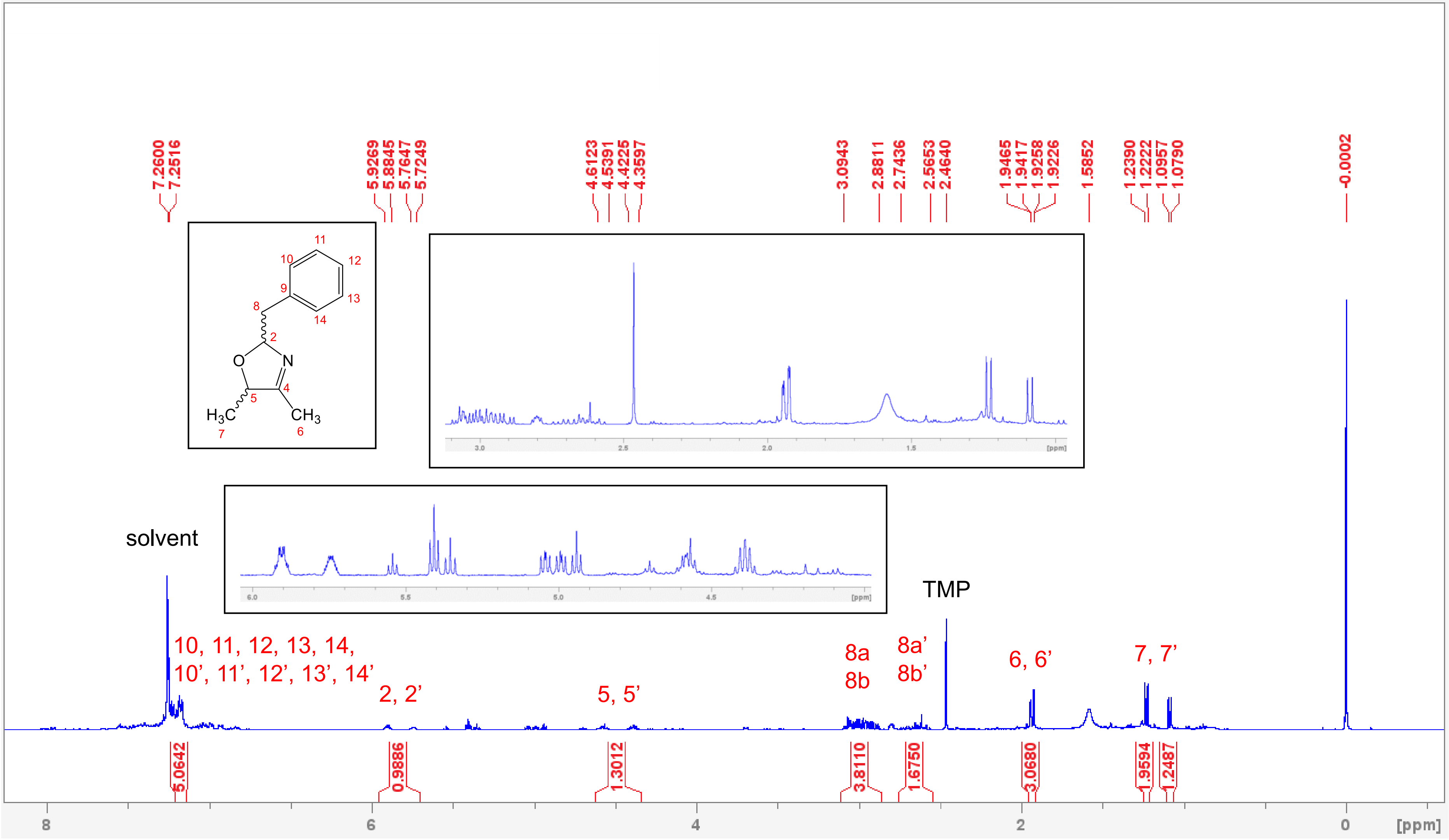

Version 01Jan

Figure S2.17: ^{13}C NMR spectrum of 2-benzyl-4,5-dimethyl-3-oxazoline.

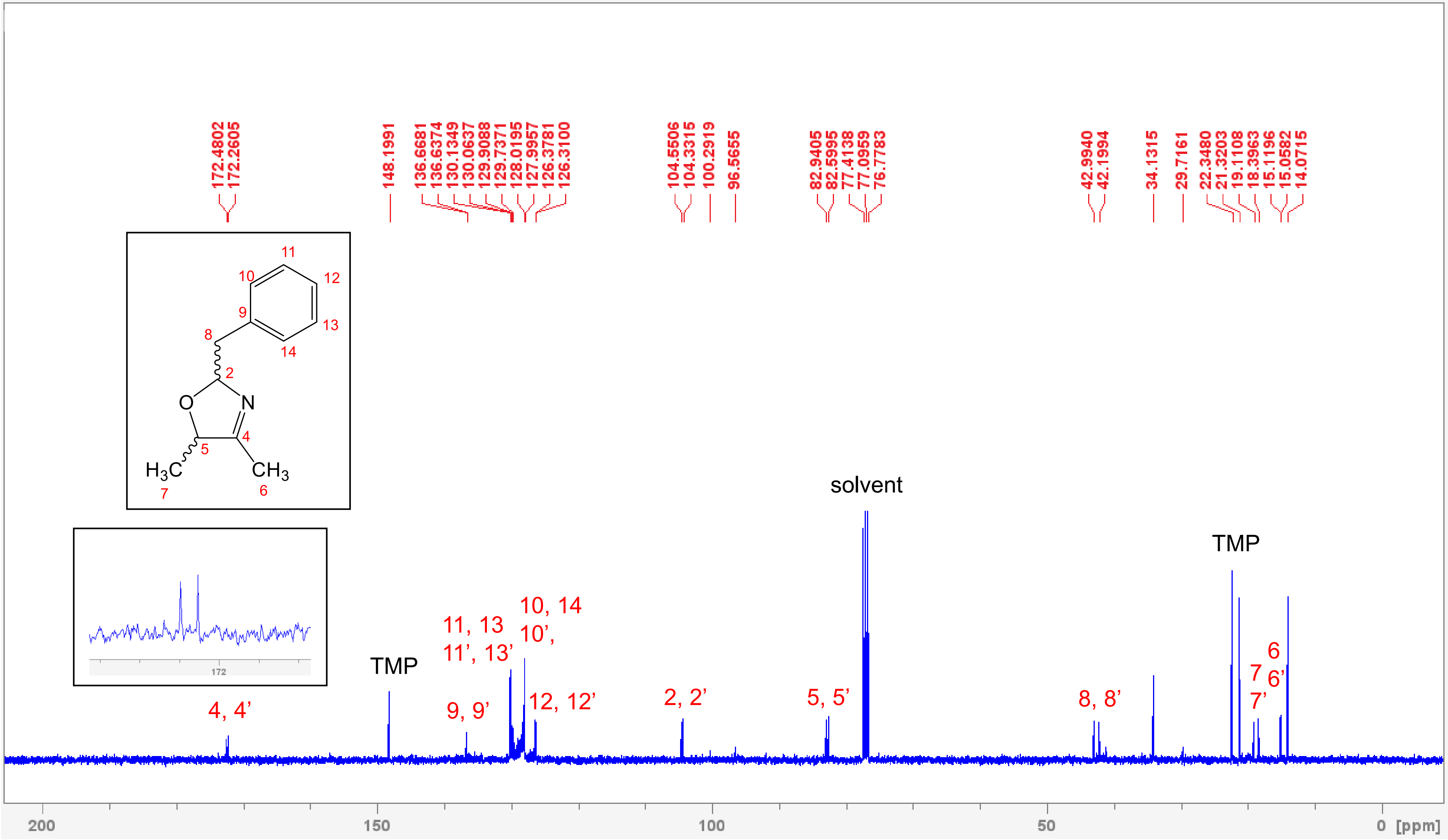

Figure S2.18: ^{1}H – ^{1}H COSY NMR spectrum of 2-benzyl-4,5-dimethyl-3-oxazoline.

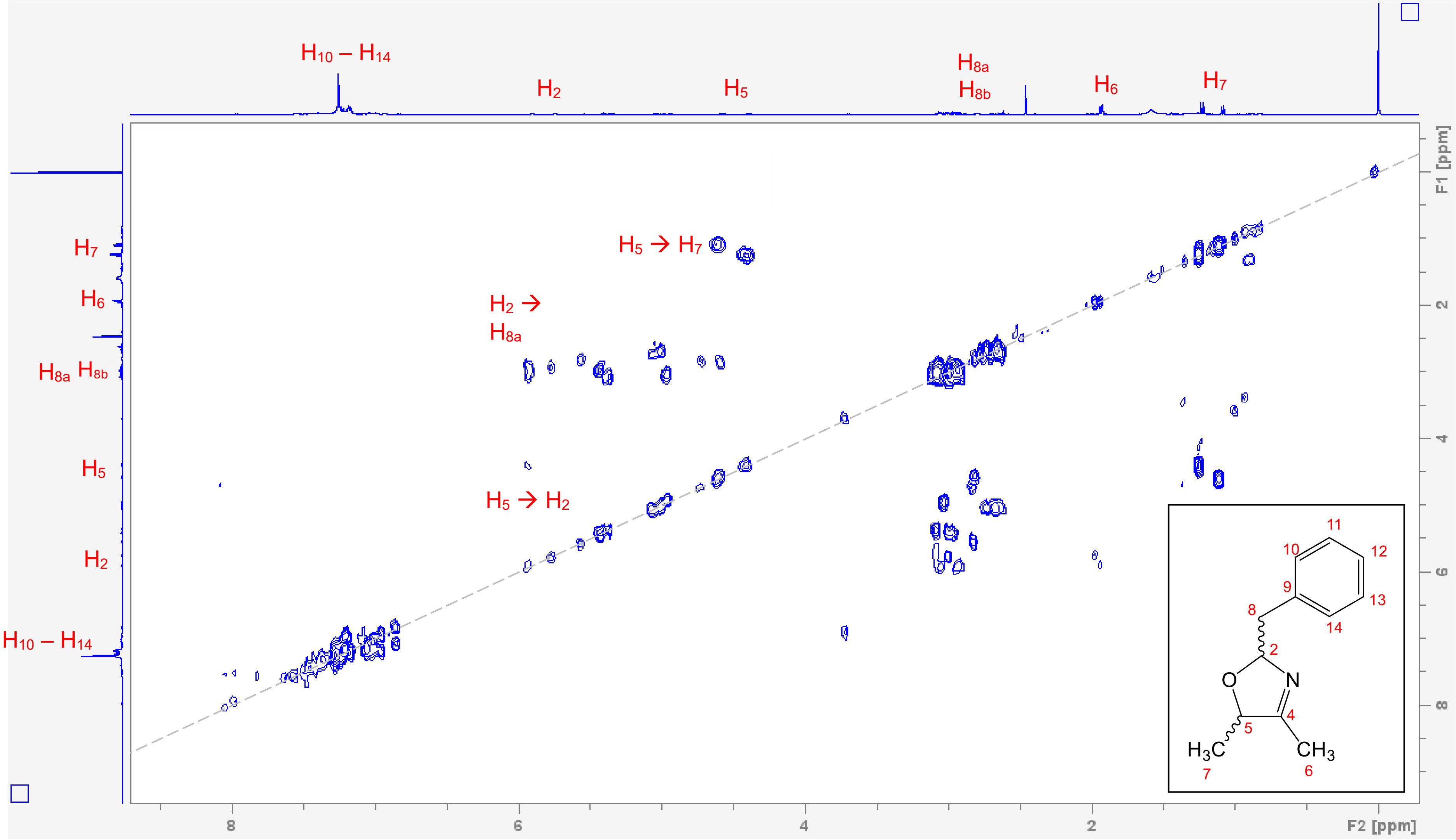

Figure S2.19: ^{1}H – ^{13}C HSQC NMR spectrum of 2-benzyl-4,5-dimethyl-3-oxazoline.

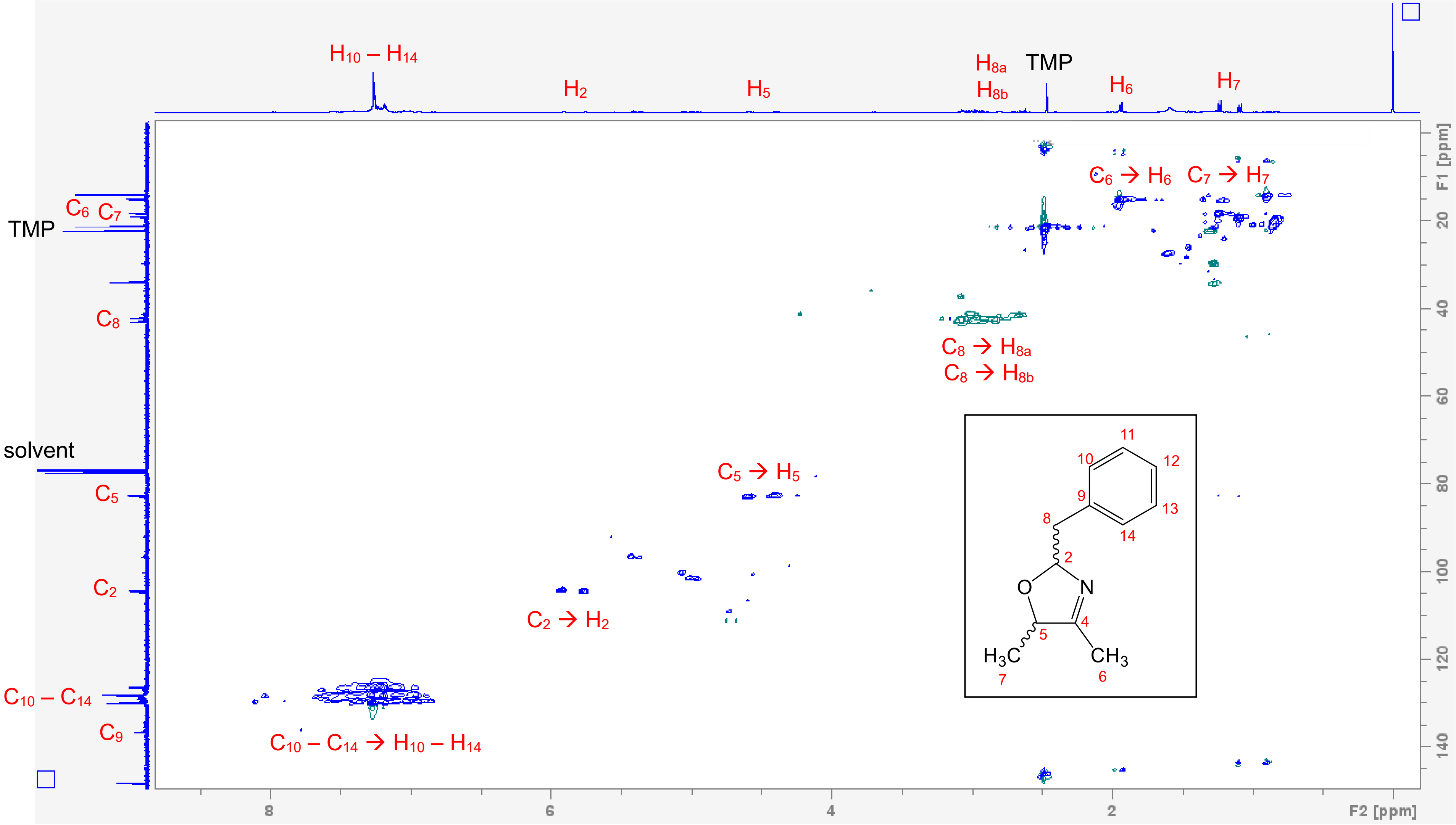

Version 01Jan

Figure S2.20: ^{1}H – ^{13}C HMBC NMR spectrum of 2-benzyl-4,5-dimethyl-3-oxazoline.

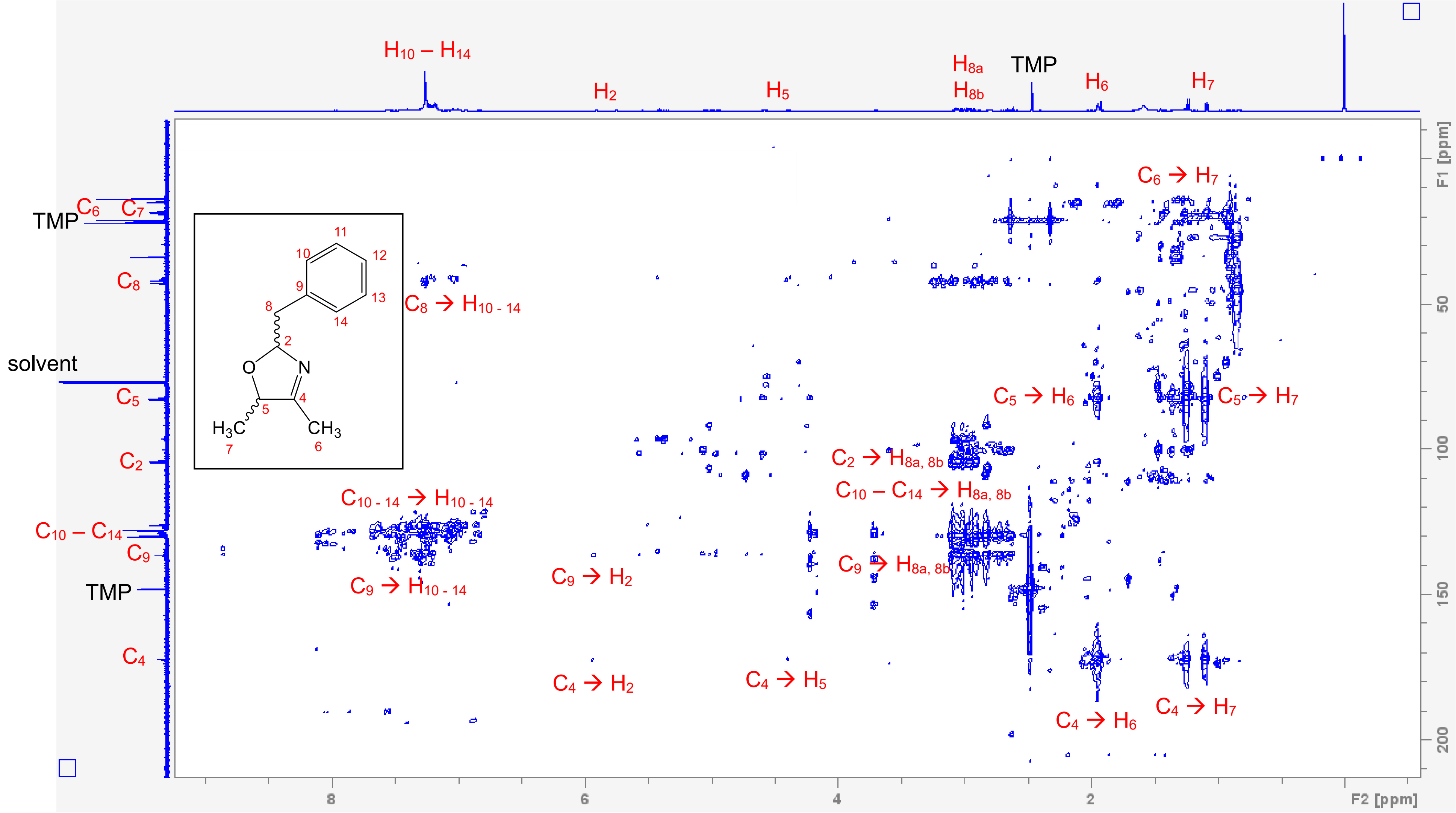

Figure S3: GC-MS analysis of the cacao nib SAFE extract spiked with either synthesized 2-isobutyl- or 2-*sec*-butyl-4,5-dimethyl-3-oxazoline. The red arrows indicate the peaks that increased due to spiking. Due to inconsistency between GC-MS runs, relative, not absolute concentrations, were compared.

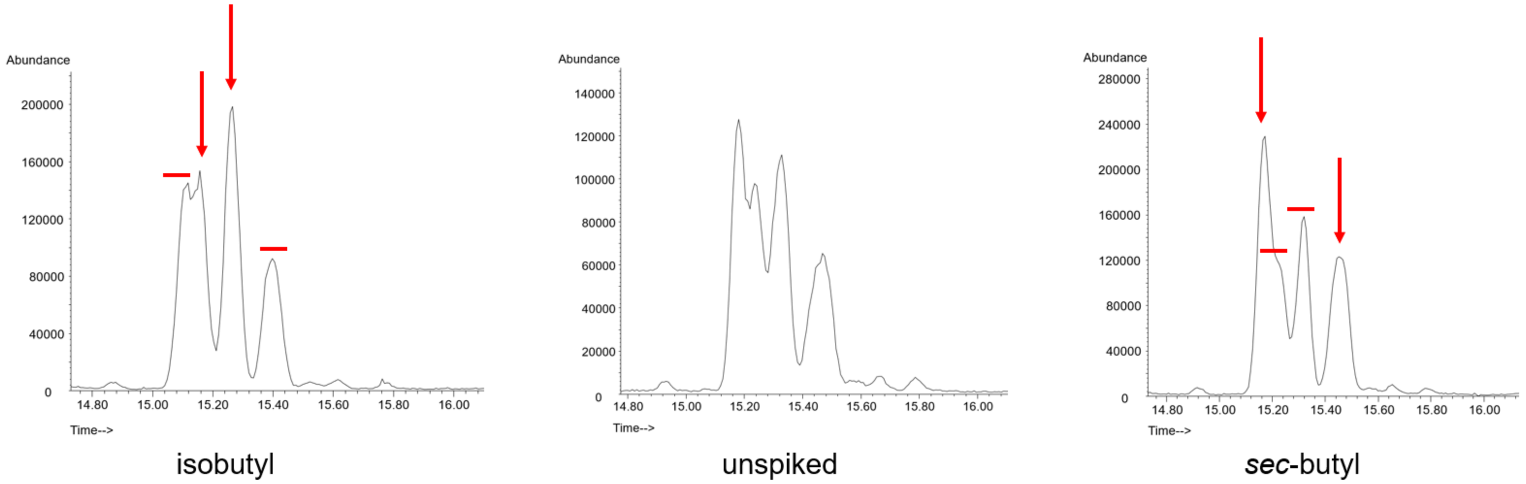

Figure S4.1: GC-MS identification of 2-isobutyl- and 2-*sec*-butyl-4,5-dimethyl-3-oxazoline in different cocoa products.

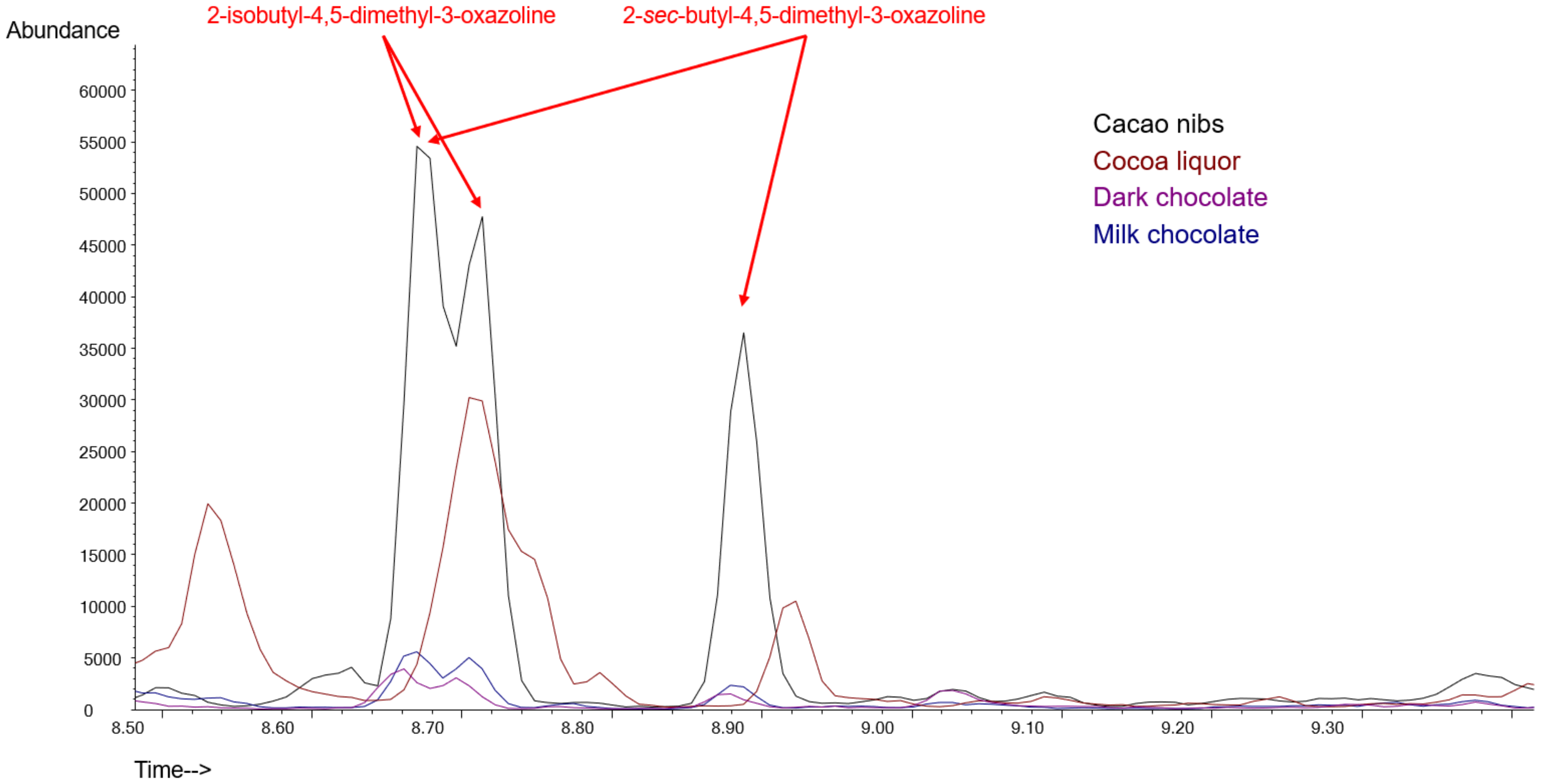

Figure S4.2: 2D-GC-MS identification of 4,5-dimethyl-3-oxazolines, **1** – **4** in cocoa liquor.

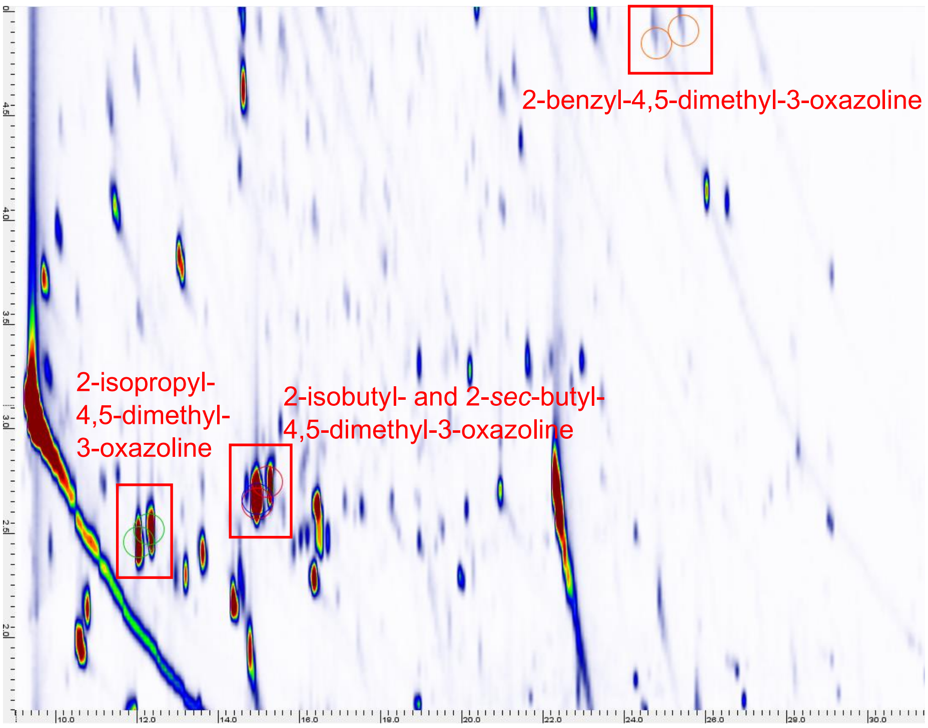

Figure S4.3: 2D-GC-MS identification of 4,5-dimethyl-3-oxazolines, **1** – **4** in milk chocolate.

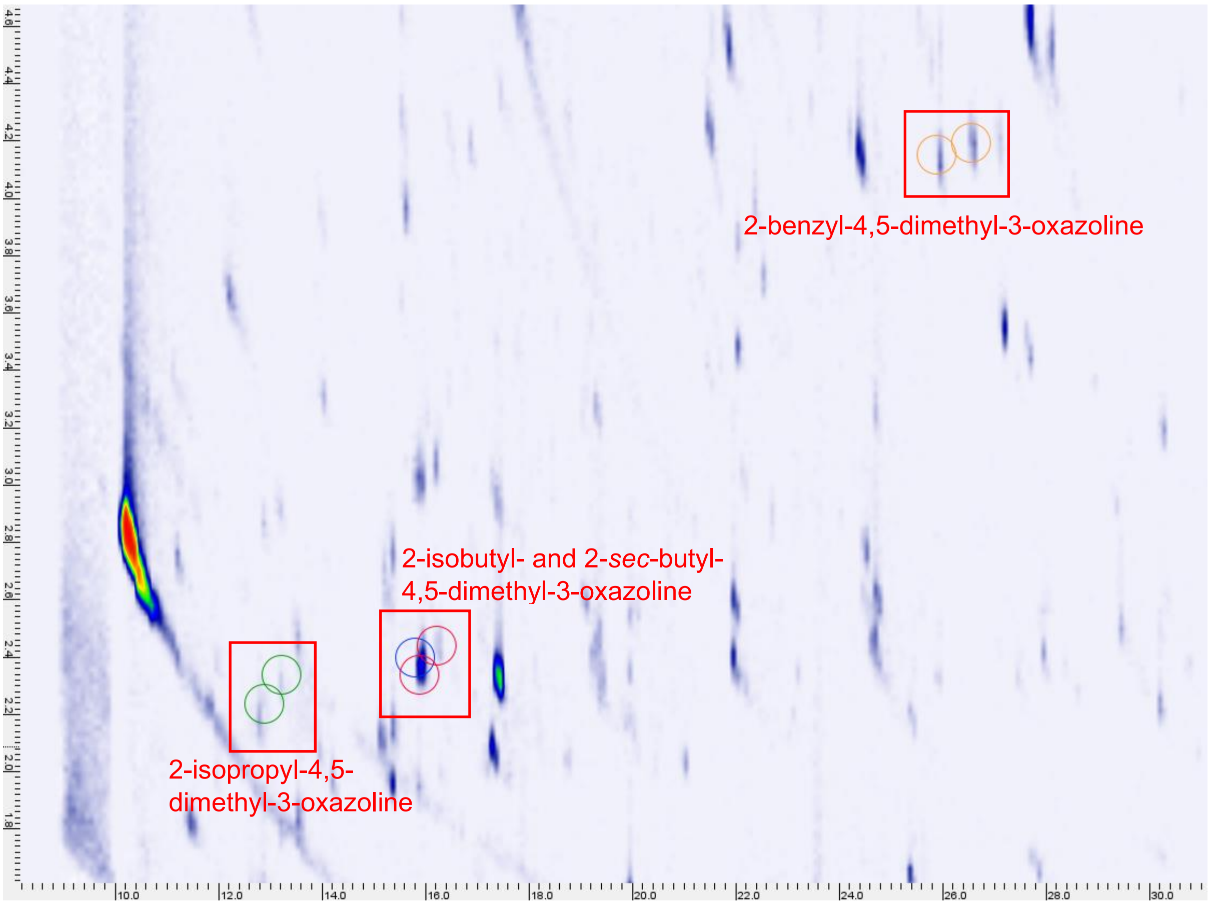

Figure S5: EI mass spectra of additionally synthesized 3-oxazolines, compounds 5 – 20.

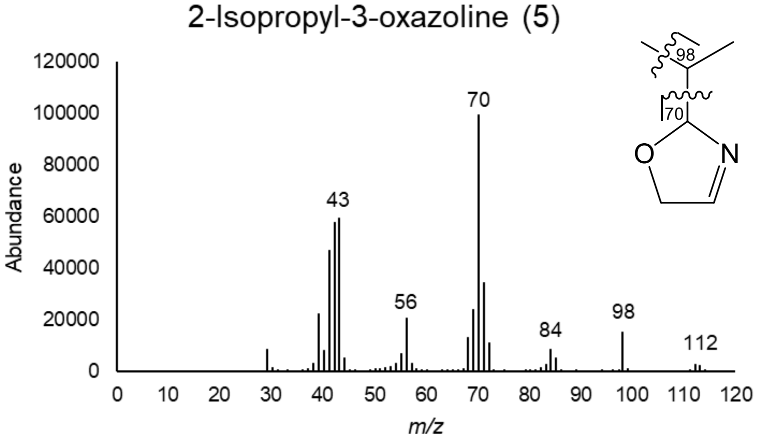

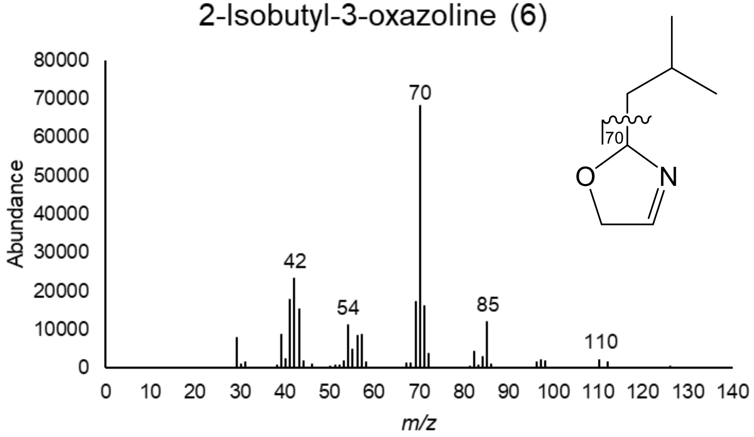

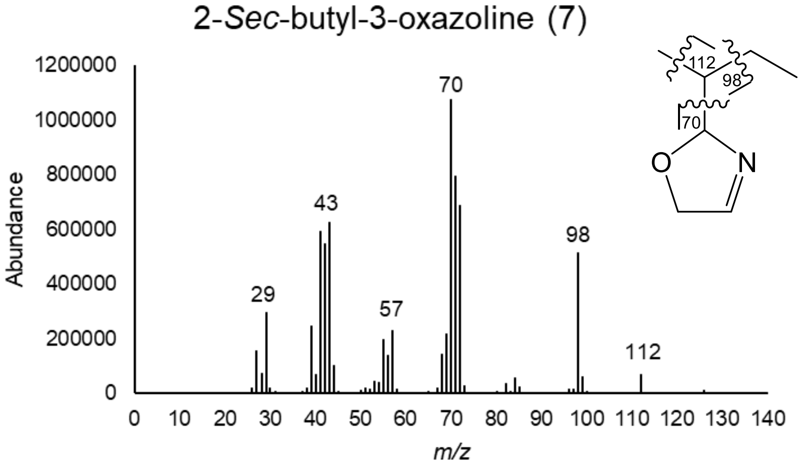

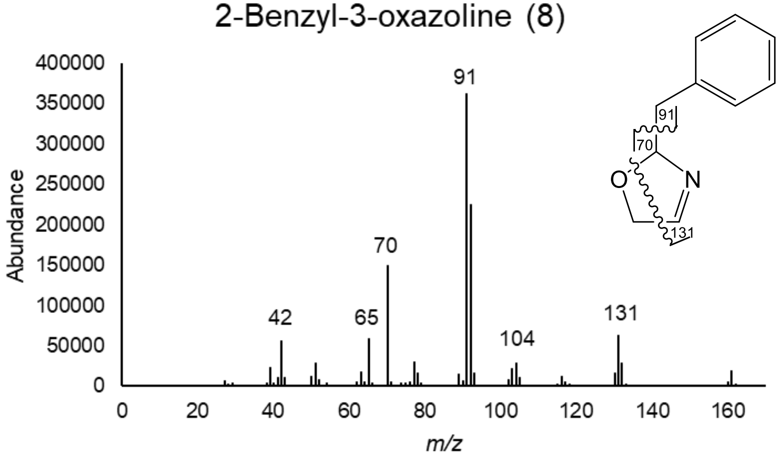

2-Isopropyl-5-methyl-3-oxazoline (9)

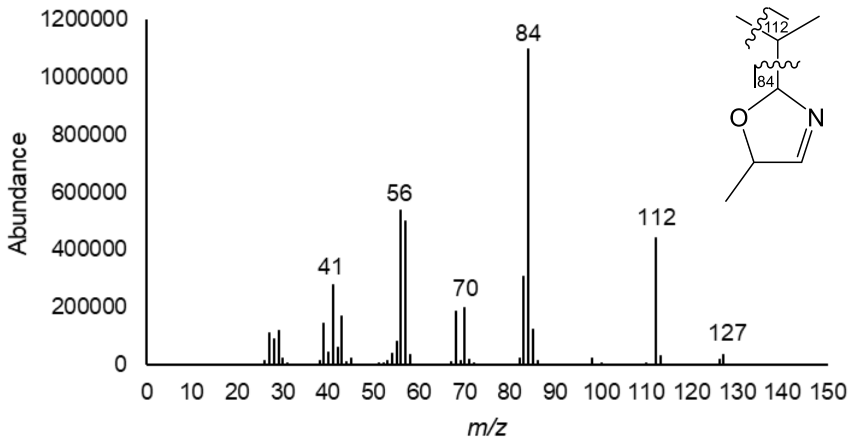

2-Isobutyl-5-methyl-3-oxazoline (10)

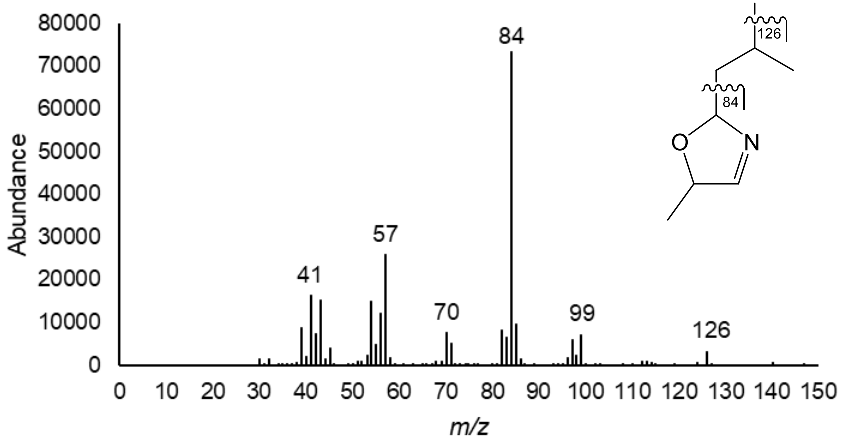

2-*Sec*-butyl-5-methyl-3-oxazoline (11)

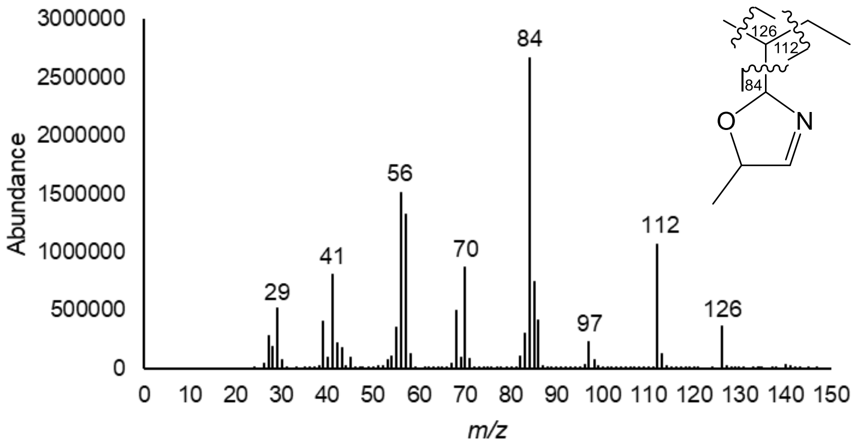

2-Benzyl-5-methyl-3-oxazoline (12)

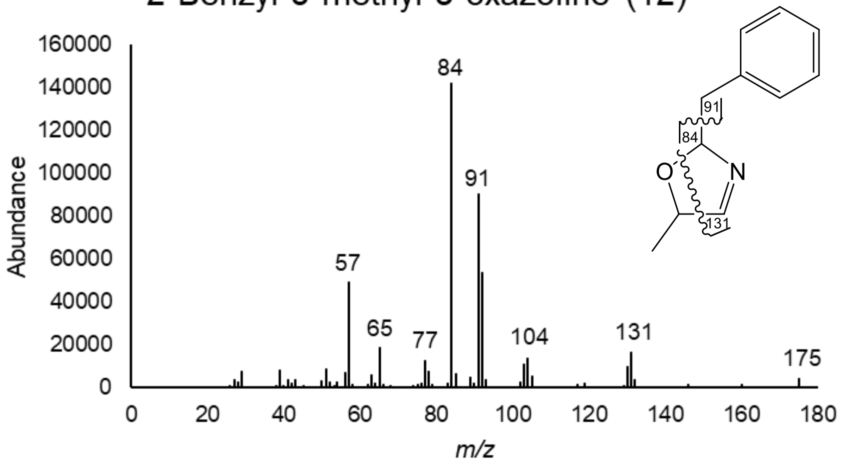

2-Isopropyl-4-methyl-3-oxazoline (13)

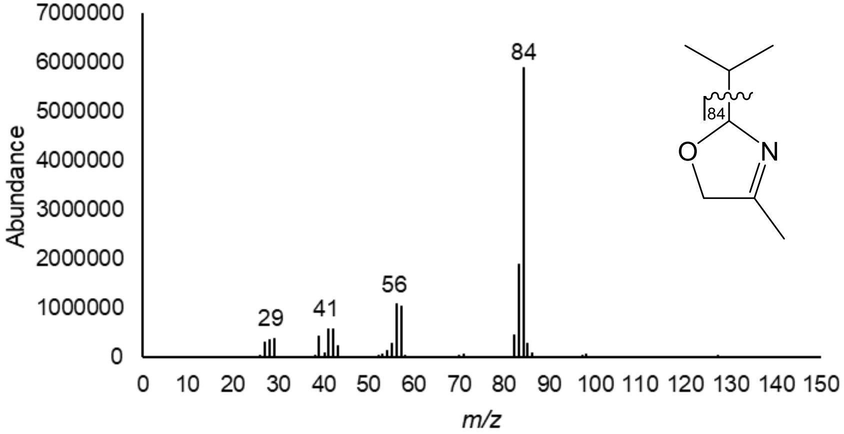

2-Isobutyl-4-methyl-3-oxazoline (14)

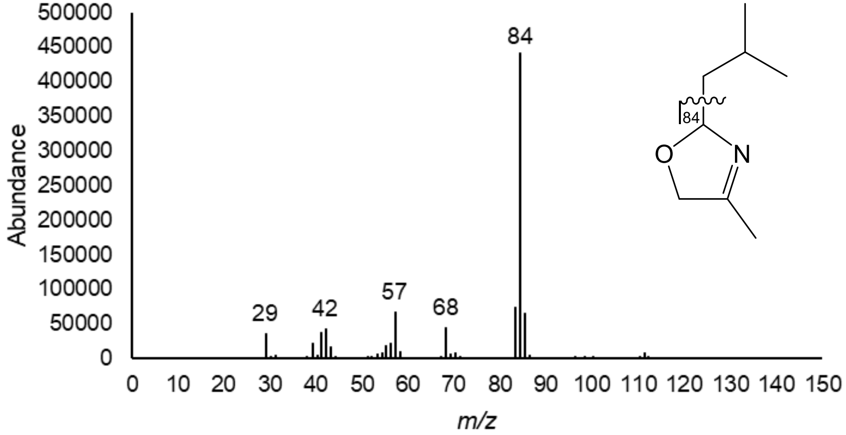

2-*Sec*-butyl-4-methyl-3-oxazoline (15)

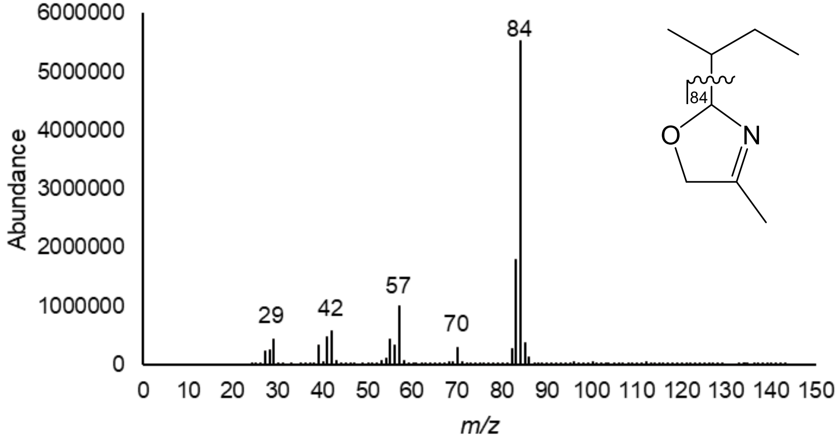

2-Benzyl-4-methyl-3-oxazoline (16)

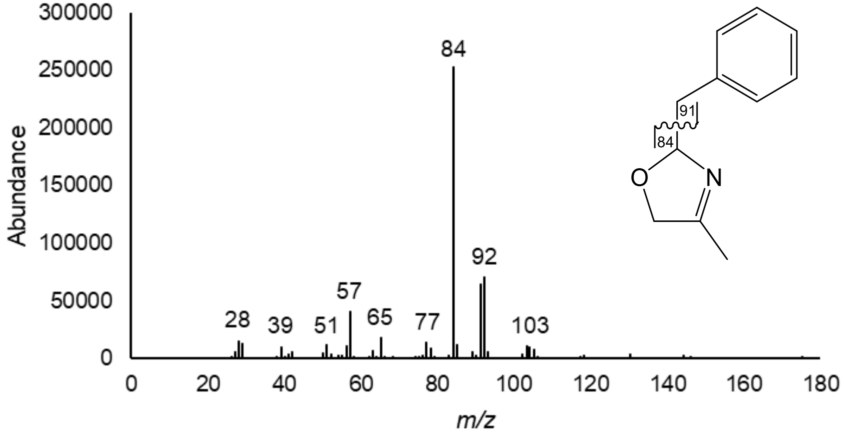

2-Isopropyl-5-ethyl-4-methyl-3-oxazoline (17a)

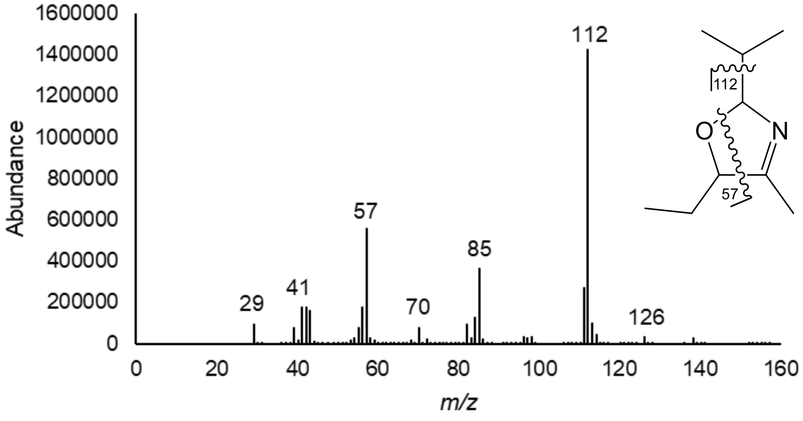

2-Isopropyl-4-ethyl-5-methyl-3-oxazoline (17b)

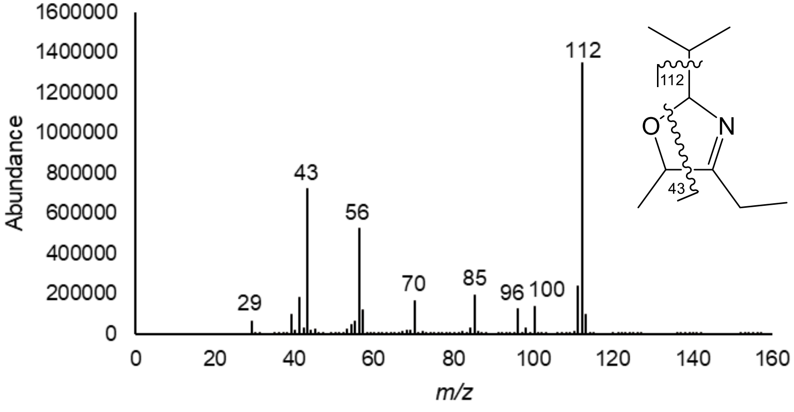

2-Isobutyl-5-ethyl-4-methyl-3-oxazoline (18a)

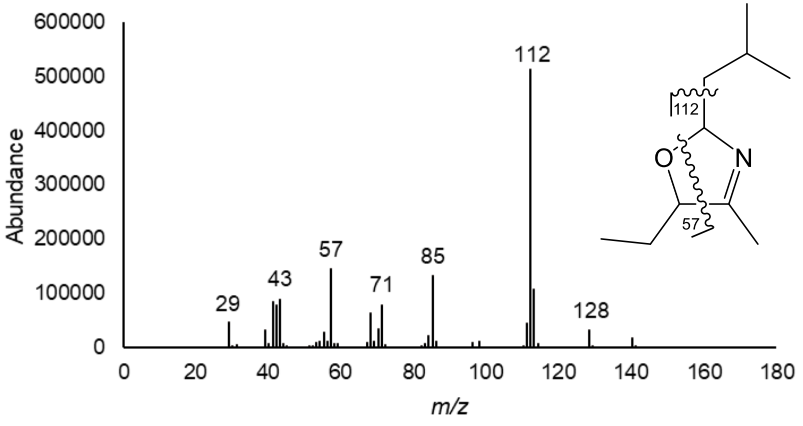

2-Isobutyl-4-ethyl-5-methyl-3-oxazoline (18b)

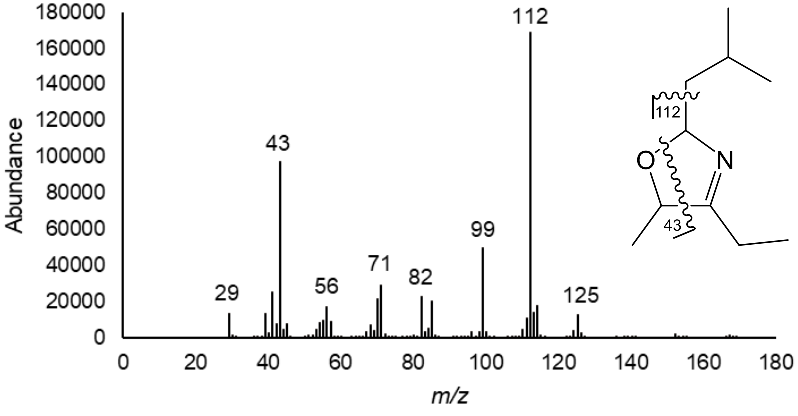

2-*Sec*-butyl-5-ethyl-4-methyl-3-oxazoline (19a)

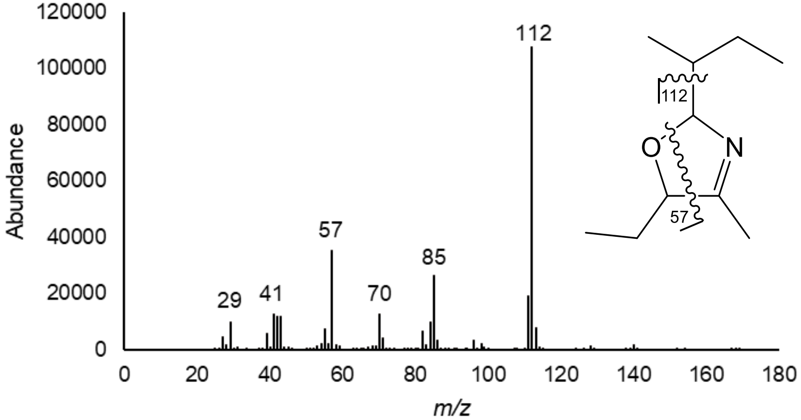

2-*Sec*-butyl-4-ethyl-5-methyl-3-oxazoline (19b)

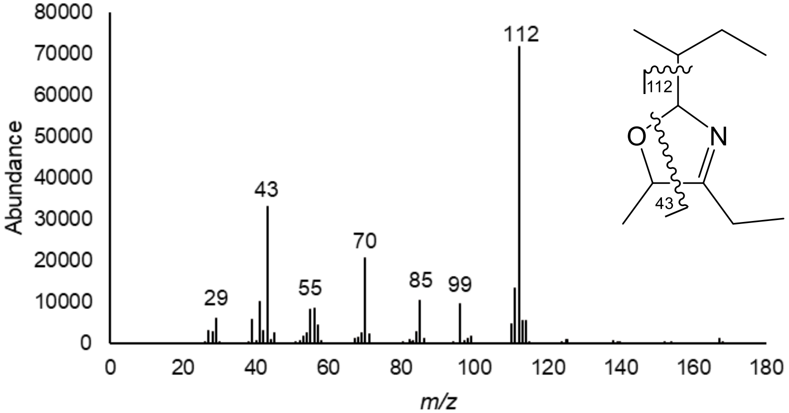

2-Benzyl-5-ethyl-4-methyl-3-oxazoline (20a)

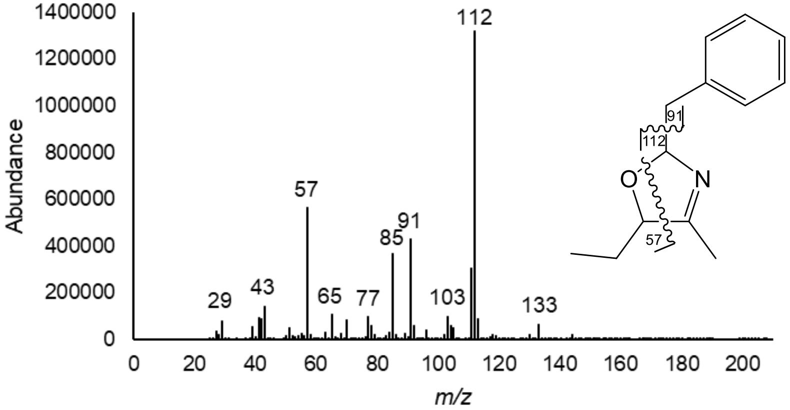

2-Benzyl-4-ethyl-5-methyl-3-oxazoline (20b)

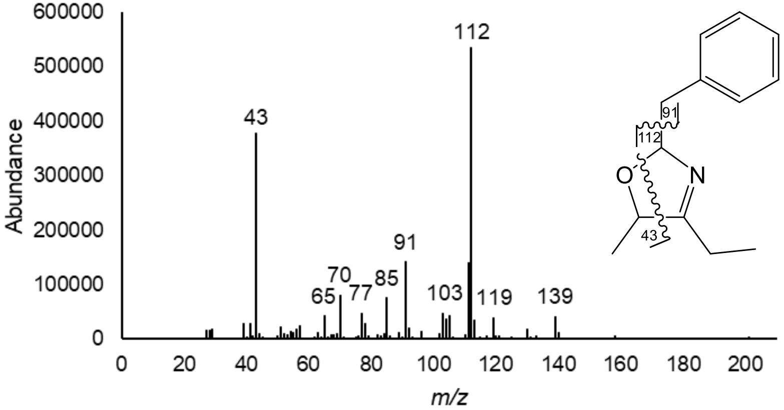

Figure S6.1: ^{1}H NMR spectrum of 2-isopropyl-5-ethyl-4-methyl-3-oxazoline (17a). 2-MP = 2-methylpropanal; 2,3-PD = 2,3-pentanedione. Four multiplet signals of H2 and H5 suggested the presence of 2-isopropyl-4-ethyl-5-methyl-3-oxazoline (17b), however this could not be confirmed, due to impurities.

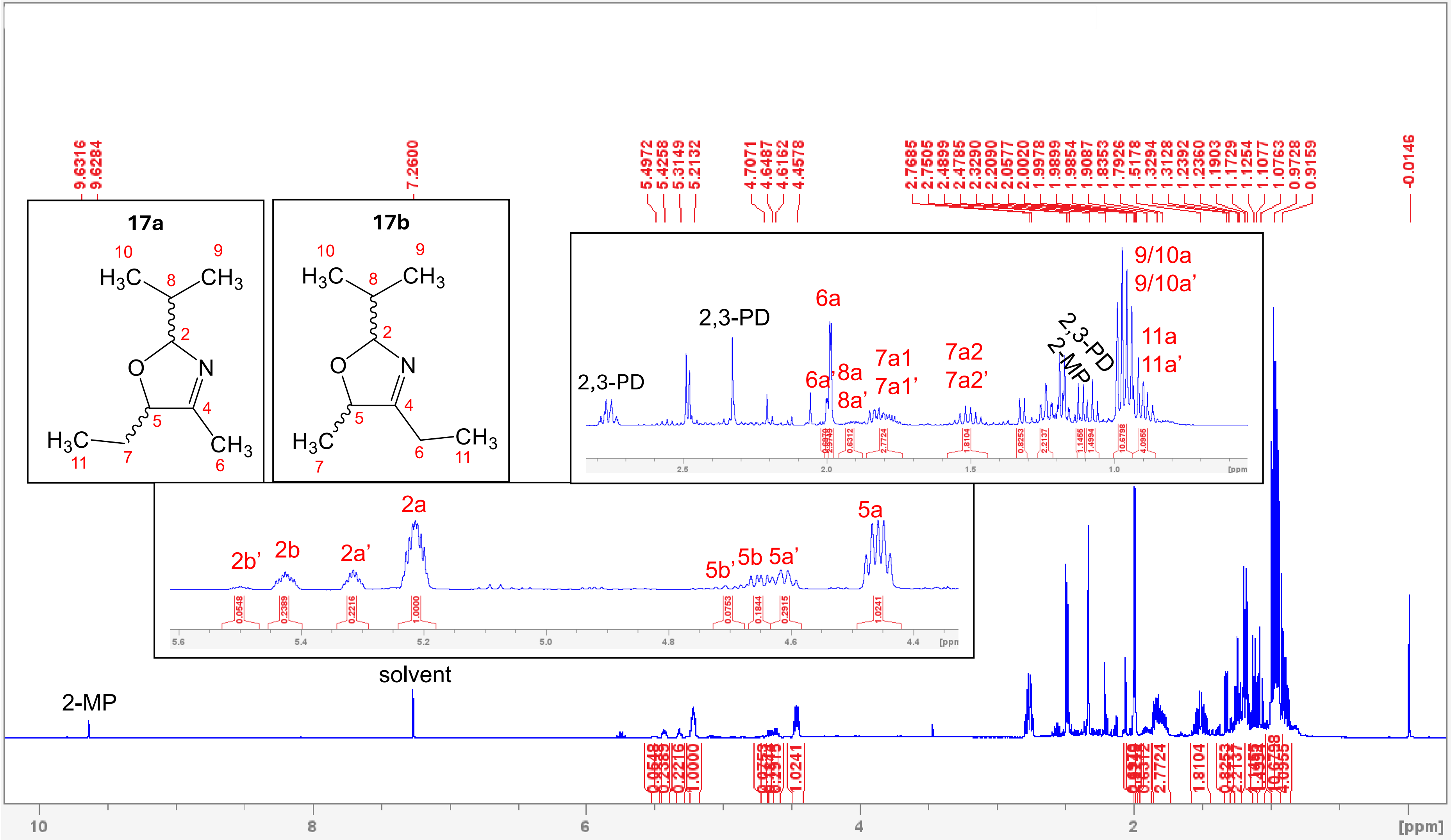

Figure S6.2: ^{13}C NMR spectrum of 2-isopropyl-5-ethyl-4-methyl-3-oxazoline.

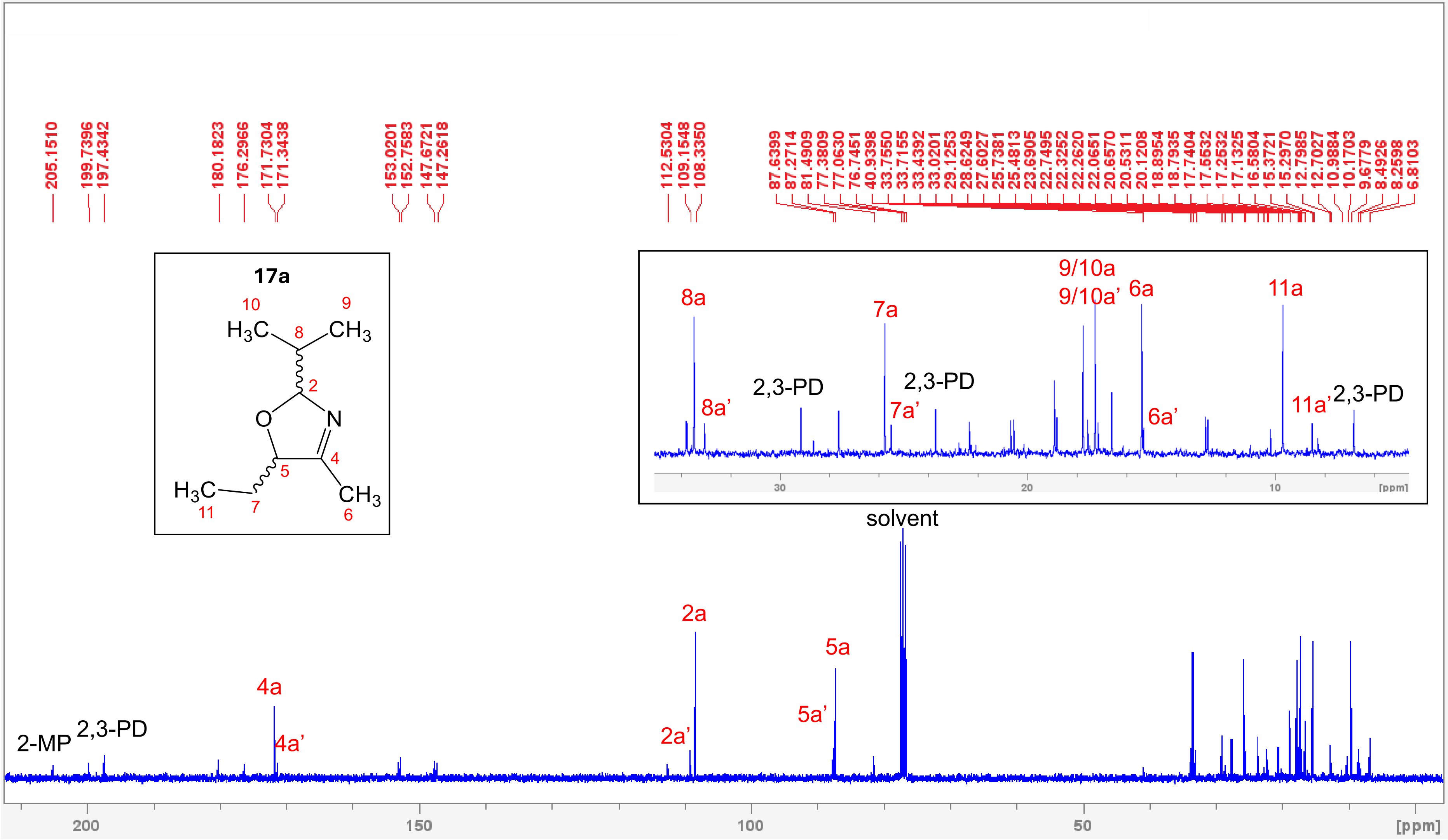

Figure S6.3: ^{1}H – ^{1}H COSY NMR spectrum of 2-isopropyl-5-ethyl-4-methyl-3-oxazoline.

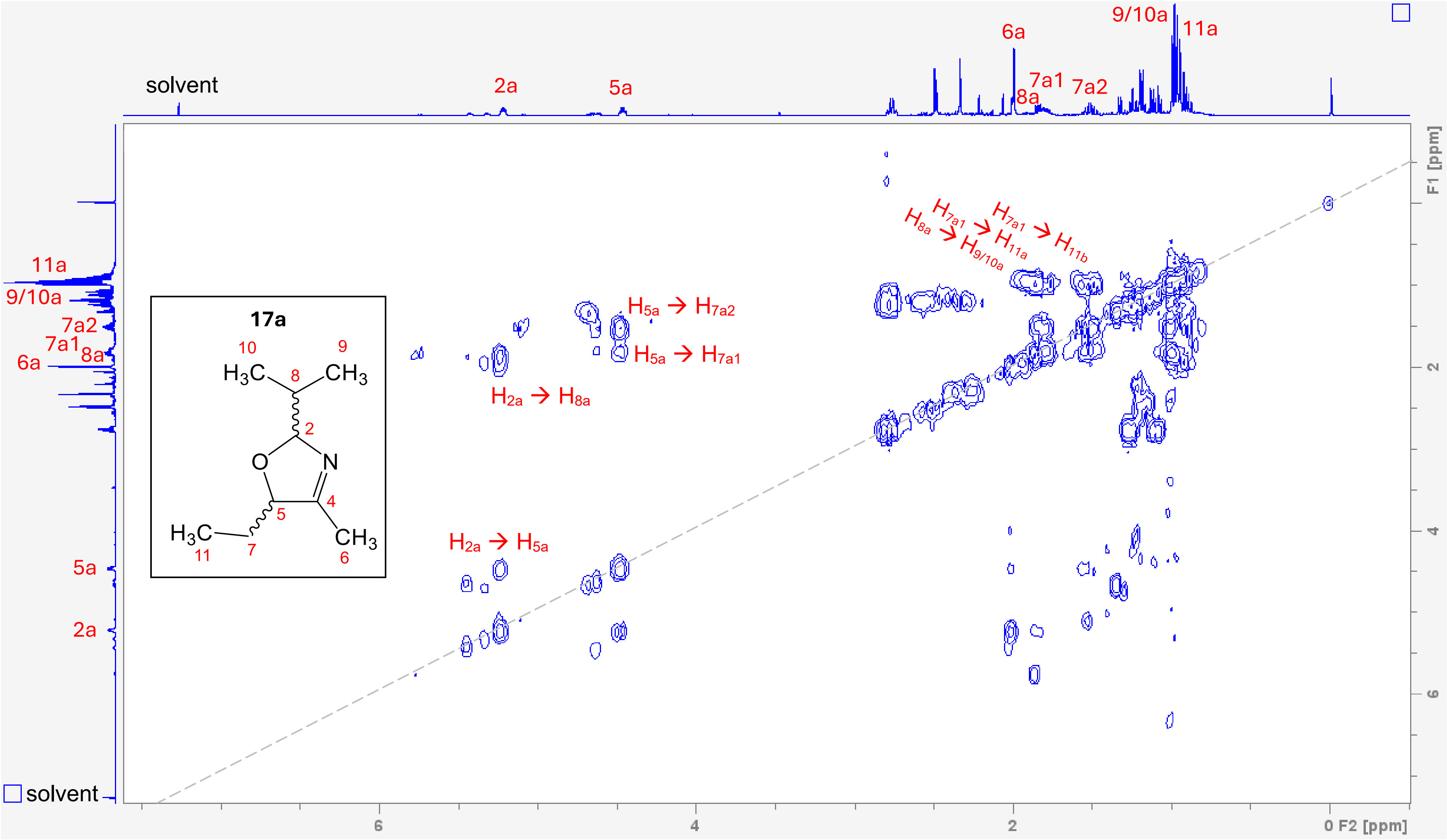

Figure S6.4: $^{1}H - ^{13}C$ HSQC NMR spectrum of 2-isopropyl-5-ethyl-4-methyl-3-oxazoline.

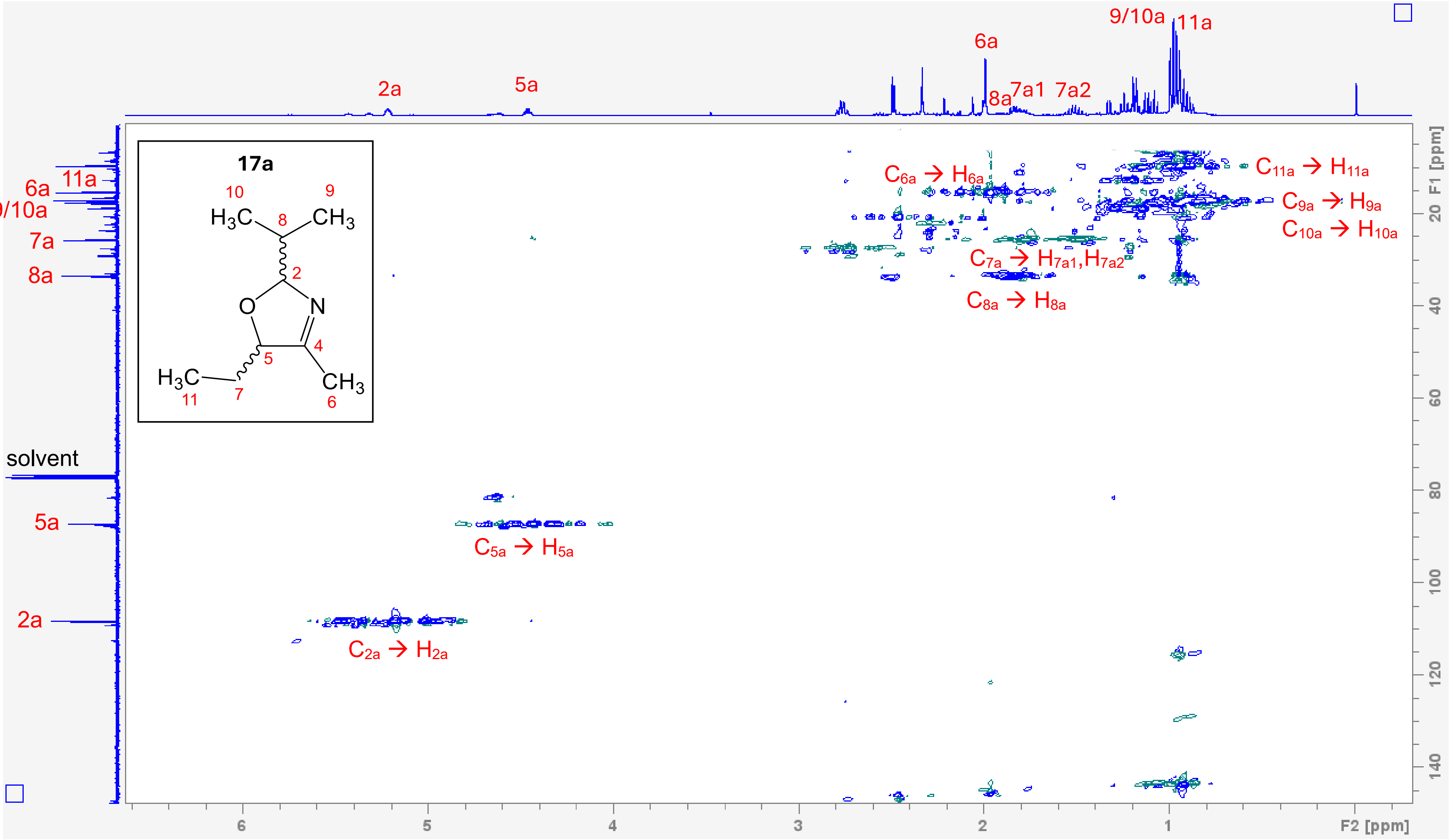

Figure S6.5: ^{1}H – ^{13}C HMBC NMR spectrum of 2-isopropyl-5-ethyl-4-methyl-3-oxazoline.

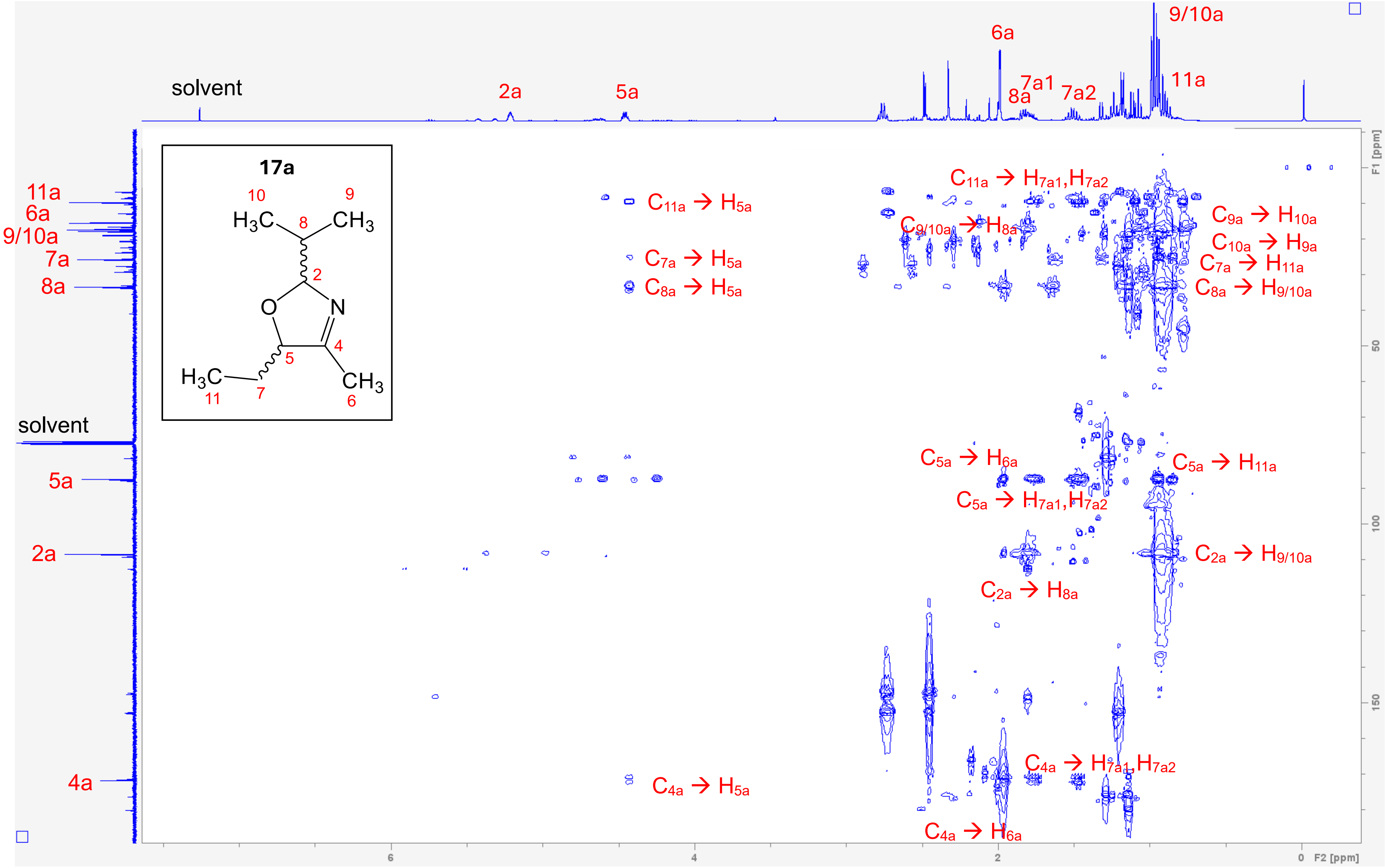

Figure S7.1: 2D-GC-MS analysis of roasted cacao nibs, extracted by SAFE, overlayed with a template created from the synthesized standards of 5/4-ethyl-4/5-methyl-3-oxazolines.

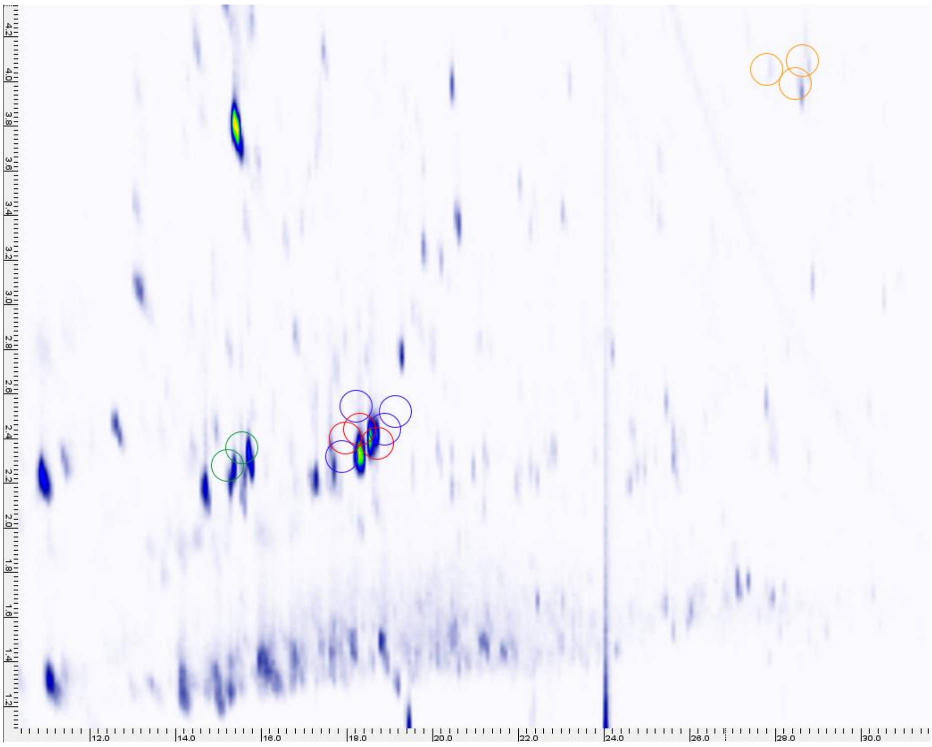

Figure S7.2: GC-MS evidence supporting the tentative identification of 5/4-ethyl-4/5-methyl-3-oxazolines in cacao nibs.

compound	LRI (HP-5)		LRI (Wax)		[M+H] exact mass	
	synthesis	cocoa	synthesis	cocoa	synthesis	cocoa
**17a/b**	1029, 1031, 1040, 1044	1031, 1034, 1042, 1046	1284, 1291, 1301, 1318	1295, 1309	156.1383	156.1385
**18a/b**	1130, 1133, 1135, 1141	1129, 1133, 1137, 1142, 1144	1382, 1384, 1400, 1410	1405, 1413	170.1539	170.1539, 170.1543
**19a/b**	1129, 1130, 1131, 1135, 1142, 1143		1371, 1373, 1378, 1387, 1382, 1391, 1405		170.1539	
**20a/b**	1495, 1497, 1523, 1532	-	1999, 2022, 2034, 2076	-	204.1383	-

End of Supporting Information.
